# Nonionotropic action of an acid-sensing ion channel inhibits leukemogenesis in the acidic bone marrow niche

**DOI:** 10.1172/JCI189051

**Published:** 2025-12-15

**Authors:** Hao Gu, Lietao Weng, Chiqi Chen, Xiaoxin Hao, Rongkun Tao, Xin Qi, Xiaoyun Lai, Ligen Liu, Tinghua Zhang, Yiming Jiang, Jin Wang, Wei-Guang Li, Zhuo Yu, Li Xie, Yaping Zhang, Xiaoxiao He, Ye Yu, Yi Yang, Dehua Wu, Yuzheng Zhao, Tian-Le Xu, Guo-Qiang Chen, Junke Zheng

**Affiliations:** 1 Key Laboratory of Tropical Translational Medicine of Ministry of Education, School of Basic Medicine, Hainan Academy of Medical Sciences, Hainan Medical University, Haikou, Hainan, China.; 2 Institute for Translational Medicine on Cell Fate and Disease, Shanghai Ninth People’s Hospital, Key Laboratory of Cell Differentiation and Apoptosis of National Ministry of Education, Department of Pathophysiology, Shanghai Jiao Tong University School of Medicine, Shanghai, China.; 3 Clinical Research Institute, The First Affiliated Hospital of Xiamen University, School of Medicine, Xiamen University, Fujian, China.; 4 Optogenetics & Synthetic Biology Interdisciplinary Research Center, State Key Laboratory of Bioreactor Engineering and; 5 Shanghai Frontiers Science Center of Optogenetic Techniques for Cell Metabolism, School of Pharmacy, East China University of Science and Technology, Shanghai, China.; 6 Research Unit of New Techniques for Live–cell Metabolic Imaging, Chinese Academy of Medical Sciences, Beijing, China.; 7 Department of Anesthesiology, Songjiang Research Institute, Shanghai Key Laboratory of Emotions and Affective Disorders, Songjiang Hospital Affiliated to Shanghai Jiao Tong University School of Medicine, Shanghai, China.; 8 Department of Anatomy and Physiology, Shanghai Jiao Tong University School of Medicine, Shanghai, China.; 9 School of Basic Medicine and Clinical Pharmacy, China Pharmaceutical University, Nanjing, China.; 10 Department of Rehabilitation Medicine, Huashan Hospital, Institute for Translational Brain Research, State Key Laboratory of Brain Function and Disorders, and Ministry of Education Frontiers Center for Brain Science, Fudan University, Shanghai, China.; 11 Hongqiao International Institute of Medicine, Shanghai Tongren Hospital, Shanghai Jiao Tong University School of Medicine, Shanghai, China.

**Keywords:** Hematology, Metabolism, Oncology, Leukemias, Stem Cells

## Abstract

The metabolic microenvironment plays important roles in tumorigenesis, but how leukemia-initiating cells (LICs) response to the acidic BM niche remains largely unknown. Here, we show that acid-sensing ion channel 3 (ASIC3) dramatically delays leukemogenesis. *Asic3* deletion results in a remarkably enhanced self-renewal, reduced differentiation, and 9-fold greater number of murine acute myeloid LICs. We developed an ultrasensitive, ratiometric, genetically encoded fluorescent pH sensor (pHluorin3) and demonstrated that LICs prefer localizing in the endosteal niche with a neutral pH range of 7.34–7.42, but not in the vascular niche with a lower pH range of 6.89–7.22. Unexpectedly, acid-ASIC3 signaling inhibits both murine and human LIC activities in a noncanonical manner by interacting with the N-terminal of STIM1 to reduce calcium-mediated CAMK1-CREB-MEIS1-LDHA levels, without inducing cation currents. This study reveals a pathway in suppression of leukemogenesis in the acidic BM niche and provides insight into targeting LICs or other cancer stem cells through pH-dependent ASICs.

## Abstract 2

An acid-sensing switch in bone marrow suppresses leukemia stem cells and reduces leukemia progression.

## Graphical abstract

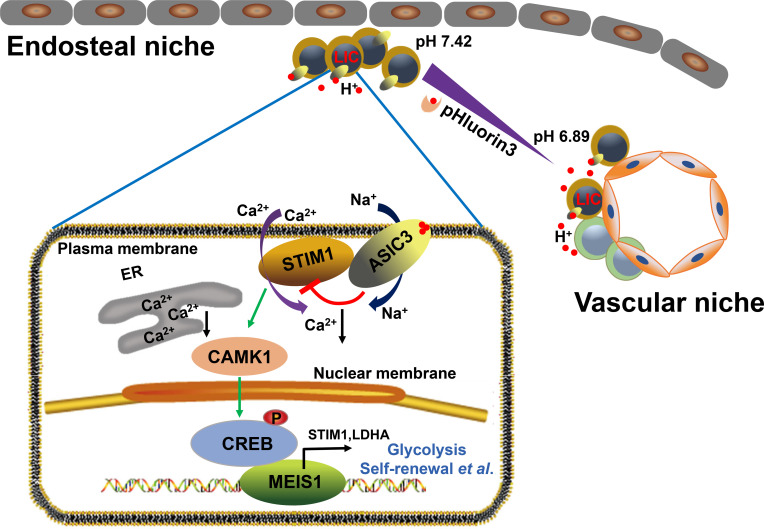

## Introduction

Leukemia-initiating cells (LICs) are considered responsible for the initiation, development, and relapse of leukemia (1, 2). Increasing evidence indicates that LICs, similar to other cancer cells, may reside in an acidic niche, due to the unique anatomic structures, disorganized cancer vasculature, and increased glycolytic levels in cancer cells (i.e., the Warburg effect) (3–7). A variety of niche components involving the maintenance LIC pool have been identified, including CCL3, GDF1, IL-6, selectins, and hyaluronic acid (8–11). We recently also revealed several niche components, including ANGPTL2, APOE, JAM3, or ATP, that can serve as critical mediators to promote leukemogenesis through their receptors of LILRB2, LILRB4, LPR5, or P2X1 and P2X7 (12–17). Identification of novel components of a (acidic) leukemic niche in the BM may be critical for targeting LICs efficiently.

It has been reported that the acidic microenvironment may contribute to enhanced tumor progression, increased mutation rate, metastasis, and resistance to chemotherapy and radiotherapy through the activation of several key oncogenic pathways (18–24). However, the low pH pressure may be lethal for tumor cells if there is lack of adaptive responses, including up-regulation of extrusion of intracellular H^+^ through cell surface transporters or reduced production of lactate (25–27). It also may be that low pH levels can reduce glucose consumption and lactate production and enhance oxidative respiration, due to the complementary effect or other unknown reasons (28, 29). However, whether LICs reside in an acidic niche and how they communicate with the acidic BM niche are not fully understood.

Currently, 2 major types of acid sensors have been identified as involved in mediating acidic signaling: acid-sensing ion channels (ASICs) and proton-sensing G-protein coupled receptors (30). Seven subunits of ASICs encoded by 5 genes have been found: ASIC1a, ASIC1b, ASIC2a, ASIC2b, ASIC3, ASIC4, and ASIC5 (31, 32). ASICs are reported to play important roles in many physiological and pathological activities in the nervous system, such as ischemia, epilepsy, and pain perception (33, 34). Interestingly, recent studies have indicated that ASICs have multifaceted functions in cancer development or other dysfunctions of multiple organs. For example, ASIC1 and ASIC2 levels are dramatically increased in glioma, and blockage of cation currents significantly delays the glioma growth metastasis (35, 36). ASIC3 is required for serotonin-mediated peripheral pain sensitivity (37), itch sensation upon coincident stimulation by acid (38), or neurogenic pathway–mediated psoriatic inflammation (39). Although most studies show that extracellular acidosis regulates neuronal cells or other types of cells through the induction of calcium channels or other signaling (40, 41), increasing evidence also indicates that calcium channels or NMDA receptor signaling may control cell fates through noncanonical conformational changes (42–47). Whether ASICs are functionally required for LICs through noncanonical manners remains largely unknown, as do the detailed connections between the BM niche and their metabolic characteristics.

Scholars have developed various approaches to determine intracellular pH. For example, several synthetic dyes, such as BCECF and SNARF, are widely used to measure cellular pH (48); however, using these dyes might result in poor control over subcellular targeting (48, 49). In addition, the leakage of these invasive tools leads to transient retention in cells (48). Genetically encoded pH indicators have been developed using GFP and its variants with different pK_A_ and spectral properties; these indicators overcome the limitations of traditional measurements (50–52). Unfortunately, most of the GFP-based pH indicators have substantial drawbacks, such as nonratiometric measurement (53)and unsuitable pK values for physiological conditions (54). In addition, some pH indicators are almost nonfluorescent at low pH (53, 55). As a pioneer pH biosensor, pHluorin exhibits admirable fluorescence property, a fine-tuned pK_A_, and ratiometric optical spectra. This tool also avoids detection errors related to protein expression level, photo bleaching, and sample thickness (50). However, the dim fluorescence caused by poor folding ability and relatively small dynamic change hinders its wide utilization in routine experimental conditions.

Here, we report on a genetically encoded pH sensor (pHluorin3) we developed that can precisely indicate the pH level in different BM niches, and we reveal that LICs tended to localize in an endosteal niche with a neutral pH range of 7.34–7.42 but not in the vascular niche with a lower pH range of 6.89–7.22. We also systematically examined the expression levels of ASICs in LICs and unraveled an unexpected role for ASIC3 in leukemogenesis. ASIC3 acts as a potent tumor suppressor to dramatically delay the leukemogenesis in a noncanonical manner, indicating that activation of ASIC3 may be an attractive way to target LICs.

## Results

### ASIC3 efficiently suppresses leukemogenesis.

In the BM microenvironment, localized acidic regions (termed “acidic niches”) have been observed under physiological and pathological conditions (56, 57). These acidic regions can influence cell fate, metabolism, and therapeutic response. However, their impact on LICs remains poorly understood. To unravel the potential connections between acidic niches and LIC activities, we established an MLL-AF9–induced murine acute myeloid leukemia (AML) model in which leukemia cells only express markers for myeloid lineages (Mac-1 and Gr-1) but not for lymphoid lineages (CD3 and B220), as described previously (12). To ask whether LICs can sense the acidic BM niche through pH-dependent ASICs, we then examined the mRNA levels of all members of ASICs (ASIC1–ASIC5), which are the main receptors for the proton, in the immunophenotypic Mac-1^+^c-Kit^+^ LICs or Lin^–^IL7R^-^Sca-1^–^c-Kit^+^CD34^+^CD16/32^+^ leukemia-associated granulocyte-monocyte progenitor cell (L-GMP) cells (a population more enriched in LICs than Mac-1^+^c-Kit^+^ cells; ref. 58) by quantitative RT-PCR (qRT-PCR).

**Figure 1:**
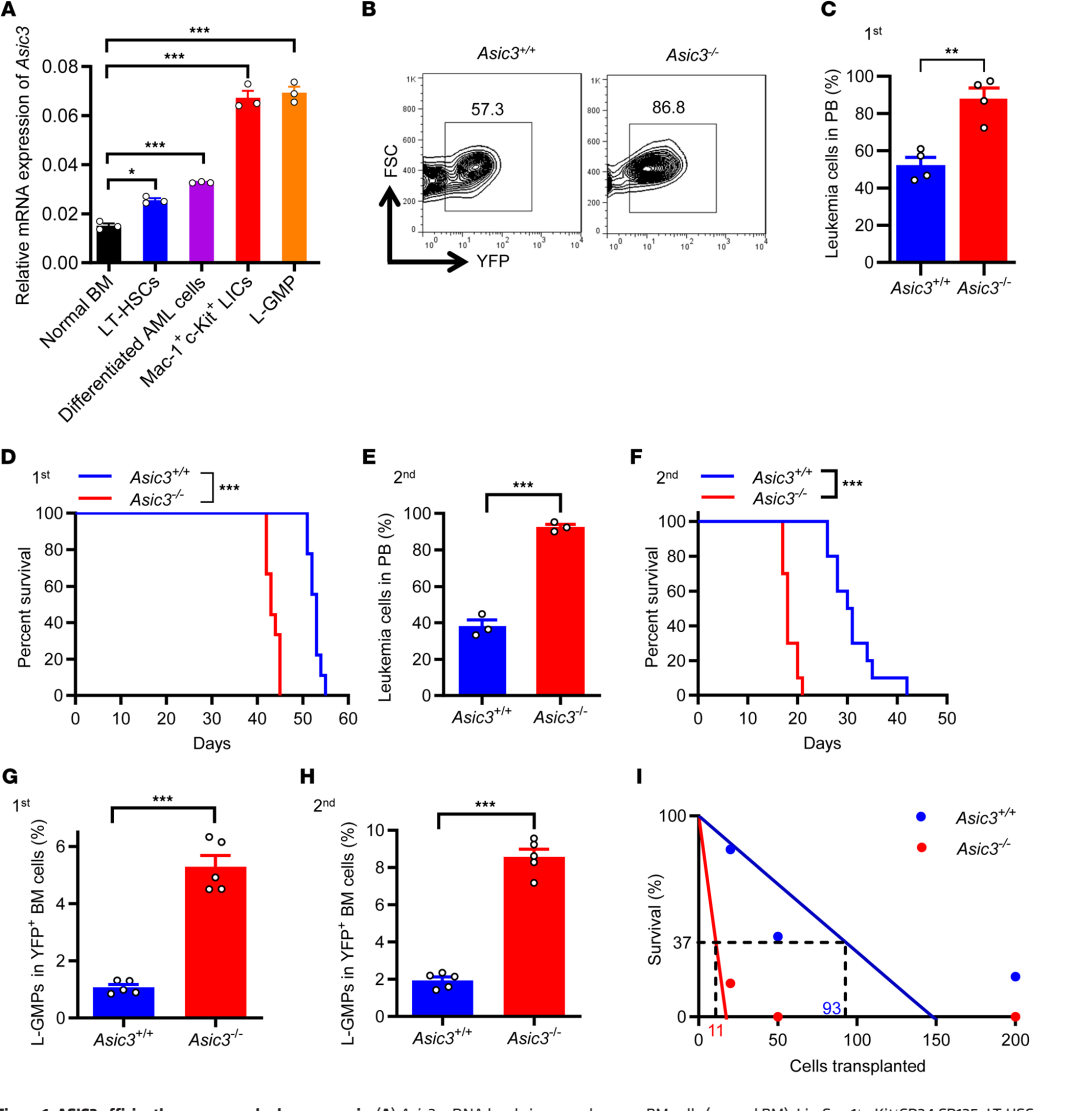
ASIC3 efficiently suppresses leukemogenesis. (**A**) *Asic3* mRNA levels in normal mouse BM cells (normal BM), Lin^–^Sca-1^+^c-Kit^+^CD34^-^CD135^–^ LT-HSCs, YFP^+^Mac-1^+^Gr-1^+^ differentiated AML cells, YFP^+^Mac-1^+^c-Kit^+^ LICs, and YFP^+^Lin^–^CD127^-^Sca-1^–^c-Kit^+^CD34^+^CD16/32^+^ L-GMPs by qRT-PCR (*n* = 3). (**B** and **C**) Flow cytometry of YFP^+^ leukemia cells in PB 5 weeks after primary transplantation (*n* = 4). FSC, forward scatter. (**D**) Survival of mice transplanted with WT or *Asic3*-KO MLL-AF9^+^ leukemia cells after primary transplantation (*n* = 9). (**E**) YFP^+^ leukemia cells in PB 2 weeks after secondary transplantation (*n* = 3). (**F**) Survival of recipients after secondary transplantation (*n* = 10). (**G**) Quantification of the percentages of L-GMP cells in the YFP^+^ BM cells from recipients 5 weeks after primary transplantation (*n* = 5). (**H**) Quantification of the frequency of L-GMPs in the YFP^+^ BM cells from recipients 2 weeks after secondary transplantation (*n* = 5). (**I**) Limiting dilution assay of LIC frequencies in WT and *Asic3*-null YFP^+^ BM leukemia cells; competitive repopulating units were calculated using L-Calc software. Data are presented as mean ± SEM. Statistical analyses were conducted with 1-way ANOVA with Tukey’s multiple comparison test (**A**), Student’s 2-tailed unpaired *t* test (**C**, **E**, **G**, and **H**), and log-rank test (**D** and **F**). *P < 0.05, **P < 0.01, ***P < 0.001.

Only *Asic1a*, *Asic1b*, and *Asic3* mRNA levels were detected in Mac-1^+^c-Kit^+^ LICs or L-GMP cells, and *Asic3* was expressed at the highest level among all the *Asic* members (Supplemental Figure 1, A and B; supplemental material available online with this article; https://doi.org/10.1172/JCI189051DS1). More surprisingly, LICs and L-GMP had the highest mRNA level of *Asic3* compared with normal BM, long-term hematopoietic stem cells (LT-HSCs), and differentiated AML cells (Figure 1A). Therefore, we examined the potential roles of both *Asic1* and *Asic3* in leukemogenesis.

Intriguingly, by using an MLL-AF9–induced AML model with *Asic1a*^–/–^ and *Asic3*^–/–^ mice, we observed that the frequencies of *Asic3*-null, but not *Asic1a*-null, leukemia cells in the peripheral blood (PB) were significantly higher than those in control mice upon primary transplantation (Figure 1, B and C, and Supplemental Figure 1, C and D). The subsequent analysis of leukemic lineages further revealed a remarkable blockage of differentiation of LICs, as indicated by the increased number of undifferentiated Mac-1^+^Gr-1^–^ myeloid cells (Supplemental Figure 1, E, F, and H), but not lymphoid cells (Supplemental Figure 1G). The accelerated leukemia development also resulted in a more severe infiltration in spleens and livers upon *Asic3* (but not *Asic1a*) deletion (Supplemental Figure 1, I and J; other data not shown). We performed Ki-67/Hoechst 33342 staining to assess the impact of *Asic3* KO on AML cell cycle progression. The results showed a decreased proportion of G0-phase cells and an increased proportion of cells in G1 and S-G2-M phases upon *Asic3* deletion (Supplemental Figure 1K), indicating enhanced cell proliferation. In addition, annexin V/PI staining revealed that *Asic3* KO reduced apoptosis in AML cells (Supplemental Figure 1L). More importantly, the overall survival of primary recipients receiving *Asic3*-null leukemia cells, but not *Asic1a*-null cells, was significantly reduced (Figure 1D and Supplemental Figure 1M), indicating that ASIC3 may suppress leukemogenesis. Much higher frequencies of *Asic3*-null YFP^+^ donor cells, undifferentiated YFP^+^Mac-1^+^Gr-1^–^ cells in both PB and BM (Figure 1E and Supplemental Figure 1, N and O), and enhanced infiltration of spleen and liver (Supplemental Figure 1P) were found in the secondary recipients. Overall survival was also significantly reduced in recipients of *Asic3*-null donor cells after secondary transplantation, compared with those receiving WT control cells (Figure 1F).

To further examine whether ASIC3 plays a specific role in AML, we used an alternative myeloid leukemia model driven by AML1-ETO9a, a splice variant of AML1-ETO. Mice transplanted with *Asic3*-deficient AML1-ETO9a^+^ cells had a significantly increased leukemia burden in PB and shorter overall survival compared with control mice (Supplemental Figure 1, Q and R).To determine how ASIC3 may influence the cell fates of LICs, we measured the frequencies of YFP^+^Mac-1^+^c-Kit^+^ LICs in BM of *Asic3*-null recipients upon serial transplantations and found there was a significant increase of *Asic3*-null LIC percentage from either primary or secondary recipients (Supplemental Figure 2, A–C). Alternatively, significantly increased frequency was observed in *Asic3*-null recipients, as indicated by the percentage of immunophenotypic YFP^+^Lin^–^CD127^-^Sca-1^–^c-Kit^+^CD34^+^CD16/32^+^ L-GMP cells (58), during both primary and secondary transplantations (Figure 1, G and H, and Supplemental Figure 2D). Consistently, an in vitro surrogate functional analysis with methylcellulose medium revealed a significant increase in colony size, colony number, and total cell number of *Asic3*-null leukemia cells during both first and second platings (Supplemental Figure 2, E–G). Subsequent limiting dilution assays with YFP^+^ leukemia cells from the primary transplant revealed that the LIC frequency was 1 in 11 of *Asic3*-null leukemia cells, which was approximately 9-fold higher than that in WT control cells (*n* = 1 in 93 cells) (Figure 1I and Supplemental Table 1). Nevertheless, the homing ability remained unchanged upon *Asic3* deletion (Supplemental Figure 2H).

To determine whether ASIC3 could influence normal hematopoiesis, we transplanted *Asic3*-null BM along with WT BM into recipients as donors in a competitive assay. Surprisingly, we found unchanged repopulation ability or multilineage potential upon *Asic3* deletion in primary and secondary competitive BM transplantation (Supplemental Figure 2, I–L). Moreover, no difference in latency and survival was detected when WT leukemia cells were injected into WT or *Asic3*-null recipients (Supplemental Figure 2M). ASIC3 was also not required for the development of B-cell acute lymphoid leukemia (B-ALL) as evaluated in an N-myc–induced murine B-ALL model during both first and second transplantations (Supplemental Figure 2, N and O). These results clearly suggest ASIC3 may be an essential tumor suppressor specifically for MLL-AF9–induced AML, but not B-ALL, through a cell-autonomous manner.

### Establishment of a highly responsive, genetically encoded pH sensor for real-time imaging of the dynamics of acidity in leukemic BM niches.

**Figure 2:**
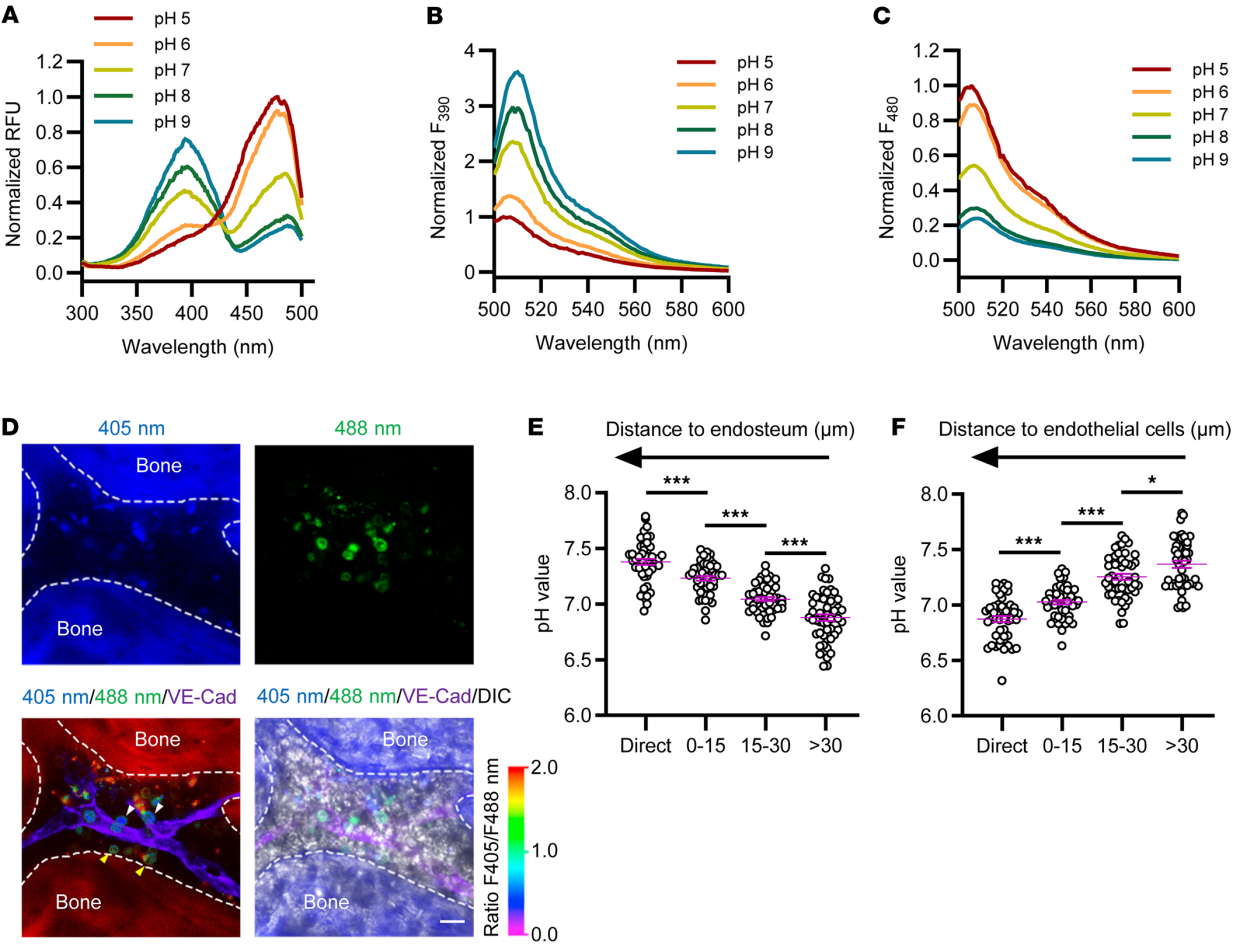
Establishment of a highly responsive, genetically encoded pH sensor for the real-time imaging of the dynamics of acidity in leukemic BM niches. (**A**–**C**) Excitation and emission spectra of purified pHluorin3 at indicated pH. RFU, relative fluorescence unit. (**D**) Imaging in calvarium focused on leukemia-colonized regions. pH levels in the endosteal niche (cells contacting endosteum >10 μm from the vascular endothelial–cadherin^+^ [VE-Cad]sinusoid) and vascular niche (cells contacting vascular endothelium >10 μm from the endosteal surface) assessed by pHluorin3 fluorescence ratio (405/488 nm). Yellow arrows indicate THP-1 cells located in the endosteal niche, and white arrows indicate those in the vascular niche. Purple regions indicate VE-cadherin^+^ vasculature, and white dashed lines denote the bone surface. Scale bar, 20 μm. (**E** and **F**) Quantification of pH levels grouped by distance to endosteum (**E**) or endothelium (**F**) (*n* = 50). Data are reported as mean ± SEM in **E** and **F**. Statistical analyses were conducted with 1-way ANOVA with Tukey’s multiple comparison test in **E** and **F**. *P < 0.05, ***P < 0.001.

Although many studies have indicated leukemic niches provide various components to regulate leukemia development (8–11), the detailed regulatory networks of such factors and the exact localization of LICs in the BM niches remain largely unknown. We recently reported that AML LICs tend to reside in the endosteal niche (59) (comprising mainly osteoblasts; ref. 60), but not the vascular niche (mainly composed of endothelial cells and their surrounding stromal cells, such as mesenchymal stromal cells and CXCL12-abundant reticular cells; ref. 61). To further precisely determine the pH level in different leukemic BM niches and their potential connections to LIC activities, we constructed a pH indicator with excellent performance. We introduced several known mutations in GFP-based pHluorin sensors (namely, F46L, F64L, S72A, Q204H, and L220F) (Supplemental Figure 3A), which accelerate protein maturation (62). The brightest clone, F46L/F64L/S72A, was further mutated proximal to the chromophore of GFP, such as at sites 69, 146, 147, 148, 203, 204, and 220, to expand the dynamic range of pH sensing. By conducting several cycles of saturated mutagenesis, we found the mutant Q204H/L220F (named pHluorin3) increases the response to pH compared with pHluorin (50) and pHluorin2 (63). The pHluorin3 sensor displayed a fluorescent spectrum similar to that of pHluorin (50), with 2 excitation peaks at 390 and 475 nm and 1 emission peak at around 510 nm (Figure 2, A–C). The sensor chromophore was protonated along with alkalization from pH 5 to 9, which led to a 3.4-fold increase in the violet peak (390 nm) and about a 5.5-fold decrease in the 475 nm peak intensity (Figure 2A). Thus, pHluorin3 has 18-fold maximum excitation ratio changes (Figure 2A), which is overwhelmingly superior to that of pHluorin and pHluorin2 (50, 63–65). Similar to pHluorin, the pK_A_ of the pHluorin3 fluorescence ratio (*R*_400/485_) is approximately 7.3, according to the titration curve in vitro (Supplemental Figure 3B). We also detected the response dynamics of pHluorin3 through the successive addition of acidic or basic buffer in different concentrations; the immediate change in the fluorescence ratio of the indicator revealed its responsive rapidity and reversibility (Supplemental Figure 3C). The ratio response of pHluorin3 is insensitive to other environmental perturbances, such as different ion compositions (eg, Na^+^, K^+^, Ca^2+^, Mg^2+^, Cl^–^, HCO_3_^–^, SO_4_^2–^, PO_4_^3–^,NO_3_^–^), reductants (reduced glutathione and dithiothreitol), and oxidants (H_2_O_2_ and diamide) (Supplemental Table 2). The quantum yields and extinction coefficients of pHluorin3 were measured using EGFP (Supplemental Table 3).

To test the performance of this indicator in mammalian cells, pHluorin3 was subcloned into the mammalian expression vector pcDNA3.1 with different targeting signals and was transiently expressed in HeLa cells. All these specific, targeted pH biosensors (i.e., nucleus-excluded, nucleus, cytosol and nucleus, and mitochondria) displayed marked green fluorescence when excited at 387 or 482 nm (Supplemental Figure 3D). The correct location of pHluorin3 was revealed by co-staining the mitochondria with Mito-tracker Red 580 and the nucleus with DAPI (Supplemental Figure 3E).

To quantify the local pH of different organelles, we stably expressed pHluorin3 sensor in the cytosol of HeLa cells and performed a ratiometric calibration of the indicator. The intracellular pH was manipulated in situ by ionophores (nigericin and monensin) in bath solutions of defined pH and high potassium ions (66) (Supplemental Figure 3F). The cells expressing pHluorin3 were equilibrated for 5 minutes after the replacement of external solution. The maximum pH response of pHluorin3 in live cells was similar to that of purified protein in the solution (Supplemental Figure 3, B and G). Based on the in situ calibration curve, we found only a subtle pH difference in the cytosol (~7.35) and nucleus (~7.44) in HeLa cells. However, the mitochondrial matrix presented quite strong alkalinity (~7.80). These pH assessments based on pHluorin3 are consistent with previous reports (53, 67).

Furthermore, we compared pHluorin3 with the current best pHluorin version, iR-pHluorin (65). When expressed in HeLa cells, the fluorescent intensities of pHluorin3 in cytosol and mitochondria were approximately 3.7-fold and approximately 2.4-fold higher than those of iR-pHluorin, respectively (Supplemental Figure 3H). It has been suggested that BM microenvironment is an acidic niche due to its low oxygen tension or unique anatomic structure (3, 68); however, due to the limitations of probes, the measured pH values are still far from satisfying (56), and the precise pH levels in different BM niches (e.g., endosteal niche, vascular niche) remain to be elucidated.

We then constructed cell surface–anchored pHluorin3 and examined its sensitivity to changes of acidic environment both in vitro and in vivo. Cell surface–anchored pHluorin3, indeed, specifically responded to the extracellular pH change but not to intracellular pH alterations, as compared with a cytosolic pHluorin3, by exposing 293T cells to solutions containing weak acids or bases (Supplemental Figure 3I). Consistently, pHluorin3-expressing THP-1 cells (an AML cell line) sensitively responded to the pH changes in a wide range in vitro, as indicated by the ratios of fluorescence (Supplemental Figure 4, A and B). pHluorin3^+^ THP-1 cells also could precisely indicate different pH levels in the BM of leukemic mice upon acidification or alkalization (Supplemental Figures 4, A and C). Strikingly, we then transplanted these cells into NOD-SCID mice and observed that the endosteal niche (in direct contact with the endosteum) has relatively neutral pH level (mean ± SEM pH 7.38 ± 0.03), whereas vascular niche (in direct contact with endothelium) tended to be acidic (mean ± SEM pH 6.87 ± 0.03) 2 weeks after transplantation (Figure 2, D–F, and Supplemental Figure 4, D and E).

To evaluate changes in the acidic BM environment throughout AML progression, we transplanted mouse MLL-AF9^+^ AML cells expressing pHluorin3 into recipient mice and monitored pH levels in distinct BM niches at different disease stages. The pH± SEM in the endosteal niche decreased from 7.42 ± 0.03 to 7.34 ± 0.04, whereas the pH in the vascular niche dropped from 7.22 ± 0.03 to 6.89 ± 0.03 during the progression from early to late stages of AML (Supplemental Figure 4, F and G). Based on our previous study showing that LICs preferentially reside in the endosteal niche (59), we hypothesized, therefore, that LICs tend to inhabit the endosteal niche with a pH range of 7.34 to 7.42. To investigate the pH distribution in a healthy (nonleukemic) BM microenvironment, we transplanted lineage-negative cells expressing pHluorin3 into recipient mice. This allowed us to assess the pH in the normal BM niche. We found that, in healthy mice, the mean ± SEM pH in the endosteal niche was 7.40 ± 0.02, and the vascular niche had a slightly lower mean ± SEM pH of 7.32 ± 0.02 (Supplemental Figure 4, H and I).

To verify whether the observations made in the calvarium also apply to other bones, we performed intravital imaging using 2-photon microscopy in mice transplanted with mouse MLL-AF9^+^ AML cells expressing pHluorin3 (in the AML acceleration phase). We analyzed the spatial distribution of AML cells and BM pH in both the cortical and trabecular regions of the femur. Consistent with our findings in the calvarium (Supplemental Figure 4, F and G), we observed that BM pH was higher in regions closer to the endosteum, with direct endosteal areas having pH values around 7.40 (Supplemental Figure 4, J and K). These findings indicate the BM pH is already spatially heterogeneous under homeostatic conditions and this acidity progressively increases during AML progression.

### Noncanonical acid-ASIC3 signaling inhibits leukemogenesis.

**Figure 3:**
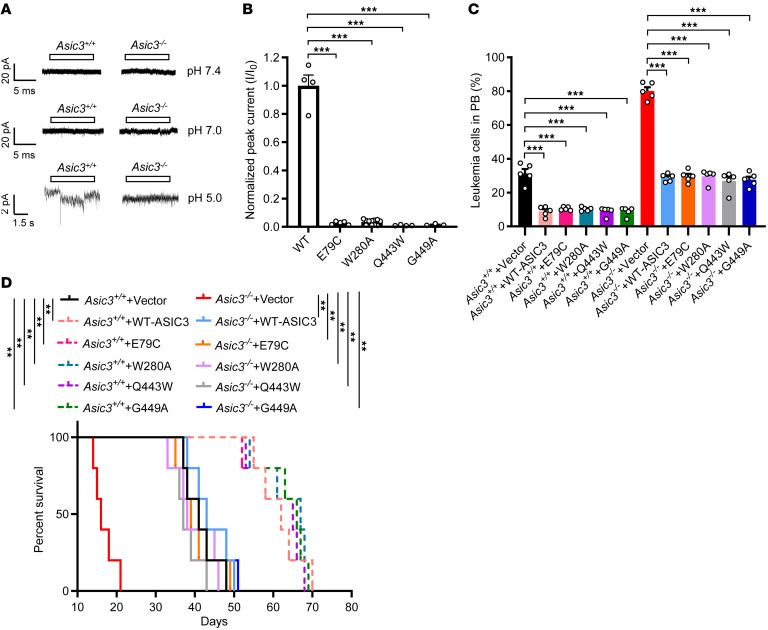
Noncanonical acid-ASIC3 signaling inhibits leukemogenesis. (**A**) Whole-cell currents in WT or *Asic3*-null LICs evoked by pH 7.4, 7.0, or 5.0, measured by patch-clamp (data are representative of 3 independent experiments). (**B**) Quantification of peak current at pH 5.0 (*n* = 3–11 CHO cells). I, peak current; I_0_, average WT current. (**C** and **D**) WT and *Asic3*-null leukemia cells overexpressing ASIC3 or its mutants were transplanted; GFP^+^ leukemia cells in PB at 2 weeks (**C**) and survival (**D**) (*n* = 5 in both) were measured. Data are reported as mean ± SEM. Statistical analyses were conducted with 1-way ANOVA with Tukey’s multiple comparison test (**B** and **C**) and log-rank test (**D**). **P < 0.01, ***P < 0.001.

Because the pH in the BM leukemic niches ranged from 6.89 to 7.42, we decided to test whether the proton from an acidic niche could induce ASIC3-mediated inward currents to affect the self-renewal and differentiation of LICs. Surprisingly, no inward currents were elicited in LICs, using whole-cell patch clamping both at pH 7.4 and pH 7.0 (Figure 3A). Although inward currents could be detected at pH 5.0 in WT LICs but not *Asic3*-null LICs, the amplitude of the currents was very small (Figure 3A). The existence of tiny or no inward currents in LICs led us to speculate that acid-Asic3 signaling inhibits leukemogenesis in a noncanonical manner. Based on prior structural and functional studies of ASIC3 channel gating (69–71); other data not shown), we selected 4 well-characterized mutants—E79C (Thumb domain), W280A (Wrist β9 loop), Q443W, and G449A (transmembrane domain 2)—that are unable to trigger inward currents. These mutants were used in rescue experiments to dissect the ion-conducting function of ASIC3 in the *Asic3* KO context. All the mutants could be efficiently expressed on the CHO cell surface membrane (Supplemental Figure 5A) and were not able to induce meaningful inward current (Figure 3B and Supplemental Figure 5B). More importantly, overexpression of these *Asic3* mutants in WT AML cells resulted in a notable extended survival of leukemic mice, whereas ectopic activation of *Asic3* mutants in *Asic3*-null AML cells could completely reverse the phenotypes caused by the *Asic3* deletion (Figure 3, C and D), indicating *Asic3*, indeed, exerts its dominant tumor-suppressing effect in a noncanonical manner without inducing a sufficient inward current.

Consistently, we also observed that the in vitro cultured medium of LICs gradually became acidic, and its pH value dropped from 7.41 to 7.00, as indicated by the pHluorin3 sensor (Supplemental Figure 5, C and D). When LICs were cultured in solution medium with neutral (pH 7.4) or lower (pH 7.0) pH conditions for 6 days and we evaluated their growth rate, we found that low-pH treatment led to a significant decrease in cell number of WT LICs but not *Asic3*-null cells (Supplemental Figure 5E). Functional analysis with methylcellulose medium with lower pH (7.0) also revealed that WT LICs had a marked reduction in CFUs or total cell number than those cultured at neutral pH (7.4) (Supplemental Figure 5, F and G). These data indicate that an acidic condition efficiently suppressed the LIC proliferation in vitro noncanonically.

### Acid-ASIC3 signaling downregulates the MEIS1 level to inhibit leukemogenesis.

**Figure 4:**
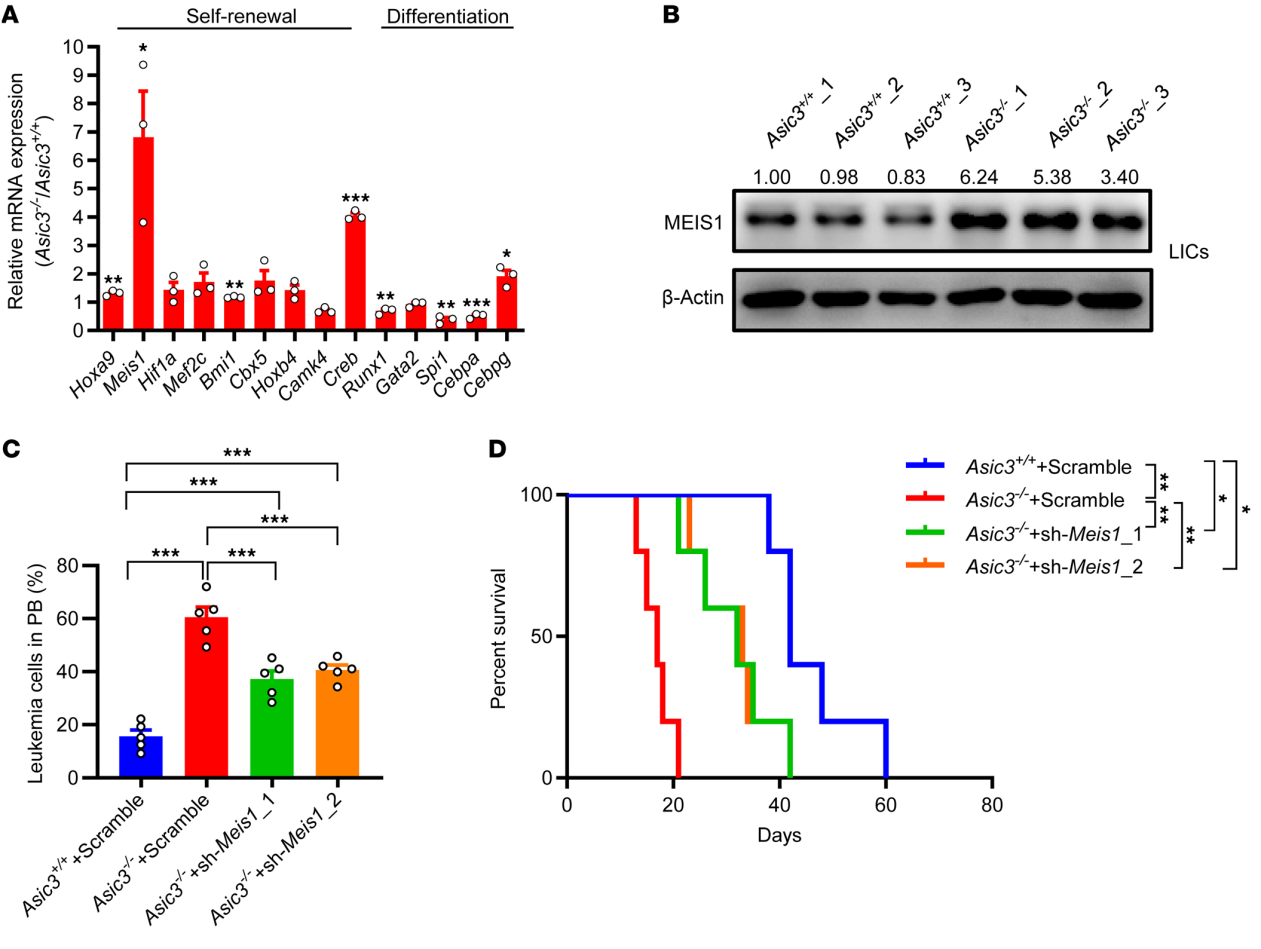
Acid-ASIC3 signaling downregulates MEIS1 level to inhibit leukemogenesis. (**A**) qRT-PCR of self-renewal genes and differentiation genes in WT and *Asic3*-null LICs (*n* = 3). (**B**) Immunoblot of MEIS1 in WT and *Asic3*-null LICs; MEIS1/β-actin ratios normalized to *Asic3*^+/+^_1. (**C** and **D**) *Meis1* knockdown in *Asic3*-null AML cells by shRNA (sh-*Meis1*_1-2), followed by transplantation; YFP^+^ leukemia cells in PB at 2 weeks (**C**) (*n* = 5) and survival in WT, *Asic3*-null, and *Meis1*-knockdown *Asic3*-null recipients (**D**) (*n* = 5). Data are reported as mean ± SEM. Statistical analyses were conducted with Student’s 2-tailed unpaired *t* test (**A**), 1-way ANOVA with Tukey’s multiple comparison test (**C**), and log-rank test (**D**). *P < 0.05, **P < 0.01, ***P < 0.001.

To further tease apart the acid-*Asic3–*mediated noncanonical signaling, RNA-Seq analyses were conducted with WT and *Asic3*-null LICs, and many leukemogenesis-related pathways were significantly changed. We observed significant alterations in the calcium ion binding and hematopoietic cell lineage pathways (Supplemental Figure 6, A and B). Given that *Asic3* deletion promoted LIC self-renewal while impairing their differentiation, and considering the well-established role of calcium signaling in regulating LIC stemness and lineage commitment (72, 73), we hypothesized that calcium signaling may contribute to the noncanonical functions mediated by *Asic3*. In line with the phenotype changes in *Asic3*-null LICs, several candidate genes involved in the self-renewal (*Meis1*, *Mef2c*, *Bmi1*, *Hoxb4*) and differentiation (*Gata2*) of LICs were significantly changed in *Asic3*-null LICs (Supplemental Figure 6C). The mRNA levels of several potential targets were further validated by qRT-PCR, and we found the mRNA levels, indeed, were distinctly upregulated (*Hoxa9*, *Meis1*, *Bmi1*, *Cebpg*) or downregulated (*Spi1*, *Cebpa*) in *Asic3*-null LICs than those in WT counterparts (Figure 4A).

Many studies have shown that the transcription factor of MEIS1, which is fine-tuned by calcium signaling (74), plays pivotal roles in the self-renewal maintenance and differentiation inhibition during leukemogenesis or other cancer development (75, 76). We noticed an approximately 6-fold increase in *Meis1* mRNA levels (Figure 4A), which prompted us to test whether MEIS1 serves as the key downstream target of ASIC3. We measured MEIS1 protein levels in both LICs and the bulk of BM leukemia cells, using Western blotting, and found that the MEIS1 levels were substantially increased in *Asic3*-null AML cells, especially in *Asic3*-null LICs (Figure 4B and Supplemental Figure 6D). Treatment under conditions slightly more acidic than pH 7.4 efficiently downregulated MEIS1 protein levels in WT LICs but not in *Asic3*-null LICs (Supplemental Figure 6E), indicating acid-ASIC3-MEIS1 signaling may inhibit LIC activities.

To examine whether MEIS1 is a downstream target of ASIC3, a rescue assay showed that the mice receiving the *Meis1*-knockdown *Asic3*-null AML cells succumbed to death much more slowly than did mice transplanted with *Asic3*-null leukemia cells, as indicated by the reduced leukemia cell frequency and prolonged survival (Figure 4, C and D, and Supplemental Figure 6F). Consistently, functional analysis, as evaluated by colony-forming assay, showed colony numbers and total cell counts derived from colonies were markedly reduced upon *Meis1*-knockdown (Supplemental Figure 6, G and H). These data prove that acid-ASIC3 signaling restrains leukemogenesis mainly through the downregulation of MEIS1 level.

### ASIC3 downregulates STIM1-CAMK1-CREB signaling to reduce the MEIS1 level.

**Figure 5:**
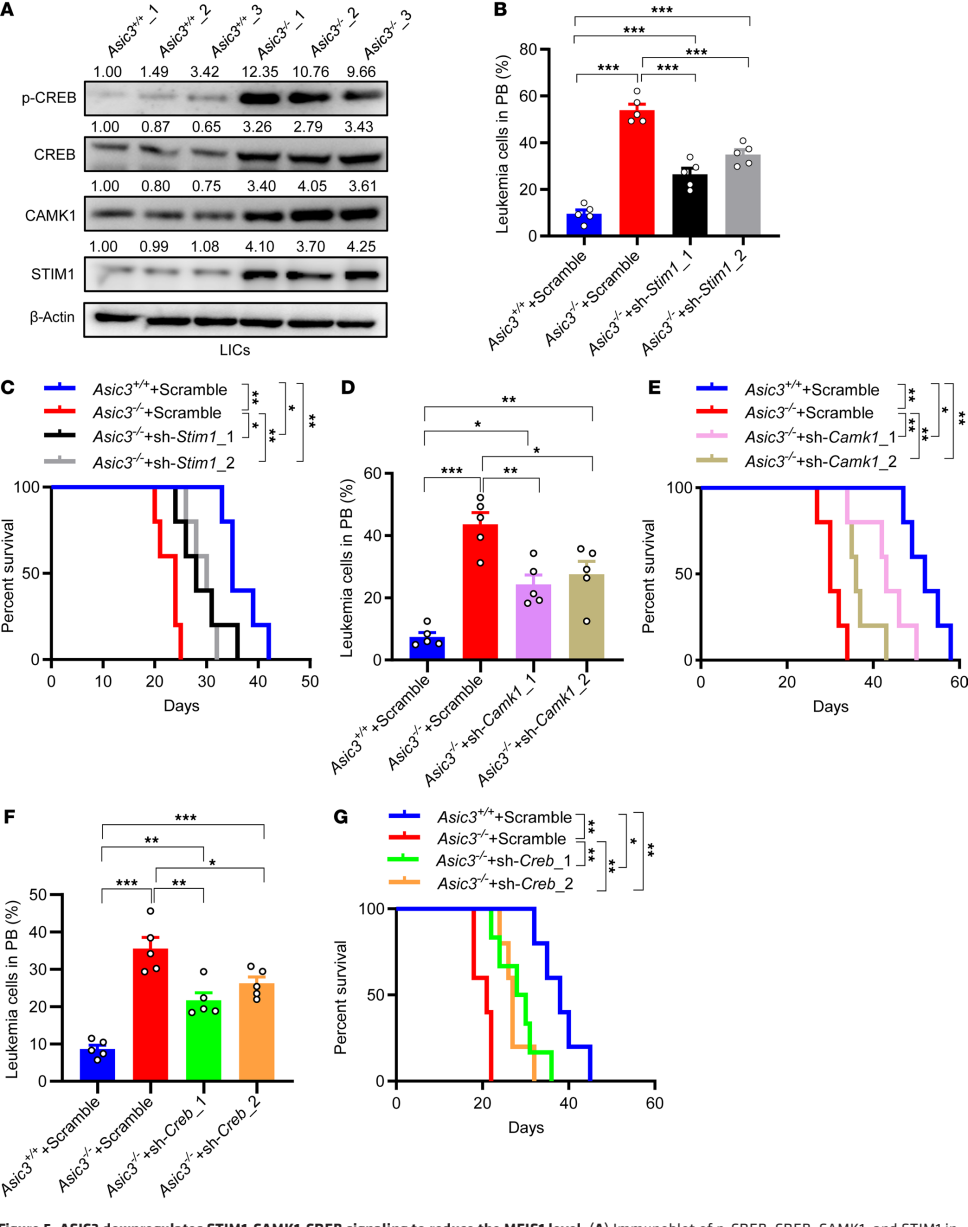
ASIC3 downregulates STIM1-CAMK1-CREB signaling to reduce the MEIS1 level. (**A**) Immunoblot of p-CREB, CREB, CAMK1, and STIM1 in WT and *Asic3*-null LICs; protein/β-actin ratios normalized to *Asic3*^+/+^_1. (**B**–**G**) *Stim1*, *Camk1* or *Creb* knockdown in *Asic3*-null AML cells, followed by transplantation. (**B**, **D**, and **F**) GFP^+^ leukemia cell frequency in PB at 2 weeks (n = 5) (**C**, **E**, and **G**) Survival among WT, *Asic3*-null, and knockdown groups (*n* = 5–6). Data are reported as mean ± SEM. Statistical analyses were conducted with 1-way ANOVA with Tukey’s multiple comparison test (**B**, **D**, and **F**), and log-rank test (**C**, **E**, and **G**). *P < 0.05, **P < 0.01, ***P < 0.001.

MEIS1 has been reported to be transactivated by the AML oncogene cAMP response element-binding protein (CREB) (74), which is tightly controlled by the mediator of calcium signaling, calcium/calmodulin dependent protein kinases (CAMKs). We sought to determine whether CREB and CAMKs were involved in the ASIC3-MEIS1 signaling pathways. Thus, we examined the protein levels of CREB in *Asic3*-null YFP^+^ BM leukemia cells by immunoblotting, which showed that both phosphorylated and total CREB levels were markedly increased (Supplemental Figure 7A). One of the key upstream regulators of CREB, CAMK1 (but not CAMK2D and CAMK4), was simultaneously upregulated in total protein levels (Supplemental Figure 7A; data not shown). Of note, higher protein levels of p-CREB, CREB, and CAMK1 were observed in *Asic3*-null LICs (Figure 5A). Because CAMK1 activity is tightly regulated by the calcium signaling, we sought to understand how calcium signaling was affected by *Asic3* deletion and found that the STIM1 level, a key mediator of calcium influx, was notably increased in LICs, as well as in YFP^+^ BM leukemia cells (Figure 5A and Supplemental Figure 7A). The elevated levels of STIM1 in *Asic3*-null LICs were also validated by immunofluorescence staining in LICs (Supplemental Figure 7B).

Currently, the function of STIM1 in leukemogenesis is largely unknown. To verify the changes of STIM1-mediated calcium signaling during leukemia development, we measured constitutive capacities of calcium influx in *Asic3*-null leukemia bulk cells or LICs. The capacities were much higher in both cell populations, especially in LICs, in comparison with WT counterparts (Supplemental Figure 7, C–F). The constitutive calcium influx was significantly reduced at pH 7.0 in either WT leukemia bulk cells or LICs, but not in *Asic3*-null cells (Supplemental Figure 7, G–J). Moreover, the elevated calcium levels in *Asic3*-null LICs could be significantly reduced by the knockdown of *Stim1* (Supplemental Figure 7, K–M). Because ASIC3 is thought to mainly mediate a certain type of cation current, such as Na^+^ (but maybe not Ca^2+^), in a pH-dependent manner (77), we speculate the upregulation of calcium levels in *Asic3*-null LICs may result from an indirect effect of the ASIC3-mediated noncanonical pathway, because no substantial inward currents could be elicited at pH 7.4 or pH 7.0, as shown in Figure 3A. When we examined whether STIM1, CAMK1, and CREB are downstream components of ASIC3 signaling, rescue assays showed that knockdown of *Stim1* or its downstream targets *Camk1* and *Creb* could largely reverse the phenotypes caused by *Asic3* deletion, as evidenced by decreased frequencies of leukemia cells in PB, extended overall survival, and reduced colony formation (Figure 5, B–G, and Supplemental Figure 7, M–U).

To further understand how ASIC3 deletion contributed to the increased calcium levels in LICs, we performed co-immunoprecipitation experiments and demonstrated that ASIC3 could directly interact with STIM1 (Supplemental Figure 8A). We further demonstrated that the N-terminal, but not C-terminal, of ASIC3, was tightly associated with STIM1 (Supplemental Figure 8B). Alternatively, we produced 2 peptides, NP-1 and NP-2, corresponding to amino acid residues 1–22 and 23–43 of the intracellular N-terminal domain of ASIC3, which is known to mediate intracellular protein interactions, to test whether they could block the constitutive calcium flux in LICs. Interestingly, we observed that NP-1, but not NP-2, could efficiently downregulate the calcium flux levels in both WT and *Asic3*-null LICs (Supplemental Figure 8, C and D), indicating the N-terminal of ASIC3 is critical for interaction with STIM1. NP-1 peptide treatment also sufficiently inhibited the phosphorylation level of CREB and cell proliferation in vitro (Supplemental Figure 8, E and F). Consistently, functional analysis, as evaluated by colony-forming assay, showed colony numbers and total cell counts derived from colonies were markedly reduced upon NP-1 peptide treatments (Supplemental Figure 8, G and H). More interestingly, MEIS1 could bind to the promoter region of STIM1 and efficiently transactivate its expression (Supplemental Figure 8, I and J). These results substantiate the concept that ASIC3 interplays with STIM1 to reduce calcium signaling in a noncanonical manner to suppress the CAMK1-CREB-MEIS1 pathway to inhibit leukemogenesis.

To explore whether other ion channels or signaling pathways might contribute to the elevated intracellular calcium levels observed in *Asic3*-null LICs, we examined the expression of several calcium influx–related molecules. Notably, we found that *Orai1* and *Trpc1* expression levels were significantly upregulated in *Asic3*-null LICs (Supplemental Figure 8K), which is consistent with previous reports indicating the ORAI1–STIM1–TRPC1 complex is essential for sustaining store-operated calcium entry (78). To functionally validate the involvement of ORAI channels, we treated LICs with Synta-66, a selective inhibitor of ORAI channels. Remarkably, this treatment completely abolished calcium influx in both WT and *Asic3*-null LICs (Supplemental Figure 8L), indicating ORAI channels are essential downstream effectors contributing to the calcium elevation observed upon *Asic3* deletion.

We also investigated whether altered sodium flux might play a role in modulating intracellular calcium dynamics, considering the canonical sodium-conducting function of ASIC3. However, our measurements revealed no marked difference in Na^+^ flux between WT and *Asic3*-null LICs under both neutral (pH 7.4) and mildly acidic (pH 7.0) conditions (Supplemental Figure 8M), suggesting that ASIC3 loss does not substantially affect sodium homeostasis in this context.

Given the possibility that *Asic3* deletion might perturb membrane potential and indirectly influence calcium channel activity, we further assessed the resting membrane potential of LICs. Our results showed no significant changes in membrane potential between WT and *Asic3*-null LICs under either pH condition (Supplemental Figure 8N), indicating ASIC3 does not exert major effects on the electrical properties of the LIC plasma membrane. Taken together, these results further underscore the functional significance of the noncanonical STIM1-interacting role of ASIC3 in modulating calcium signaling in LICs.

### ASIC3 suppresses the glycolysis of LICs to delay the leukemogenesis.

MEIS1 delicately enhances glycolytic levels to maintain the stemness of HSCs (79), and LICs may mainly use glycolysis as their energy source (59); therefore, it is conceivable that the metabolic status of *Asic3*-null LICs may be influenced by the pronounced upregulation of MEIS1. To address this possibility, we took advantage of a genetically encoded sensor (SoNar) for cytosolic NADH/NAD^+^ ratio (which mainly reflects the glycolytic level in a cell) to measure glycolysis in AML cells as indicated by the ratios of SoNar fluorescence with excitation at 405 nm and 561 nm, which can precisely monitor the subtle changes of glycolysis of AML cells in real time in different leukemic BM niches, according to previous studies from our group and others (59, 80).

We then ectopically expressed the SoNar in WT and *Asic3*-null LICs and measured their glycolytic status. *Asic3*-null LICs had much higher levels of SoNar and were more sensitive to be reduced or elevated upon pyruvate (a substrate catalyzed by lactate dehydrogenase A (LDHA) to form the lactate while consuming NADH) or oxamate (an LDHA inhibitor to increase cytosolic NADH/NAD^+^ level) stimulation (Supplemental Figure 9, A–D), which was consistent with the much lower levels of ATP and the oxygen consumption rate (an indicator of oxidative phosphorylation levels) (Supplemental Figure 9, E and F), but higher levels of extracellular acidic rate (an indicator of lactate levels) (Supplemental Figure 9G) or absolute concentration of lactate secretion (Supplemental Figure 9H) in *Asic3*-null LICs. *Asic3*-null LICs also had much lower levels of mitochondrial DNA than did WT counterparts (Supplemental Figure 9I). Acidification treatment also led to a marked decrease of the ratios of SoNar fluorescence in WT, but not in *Asic3*-null LICs, suggesting that low pH inhibits glycolysis (Supplemental Figure 9J).

**Figure 6:**
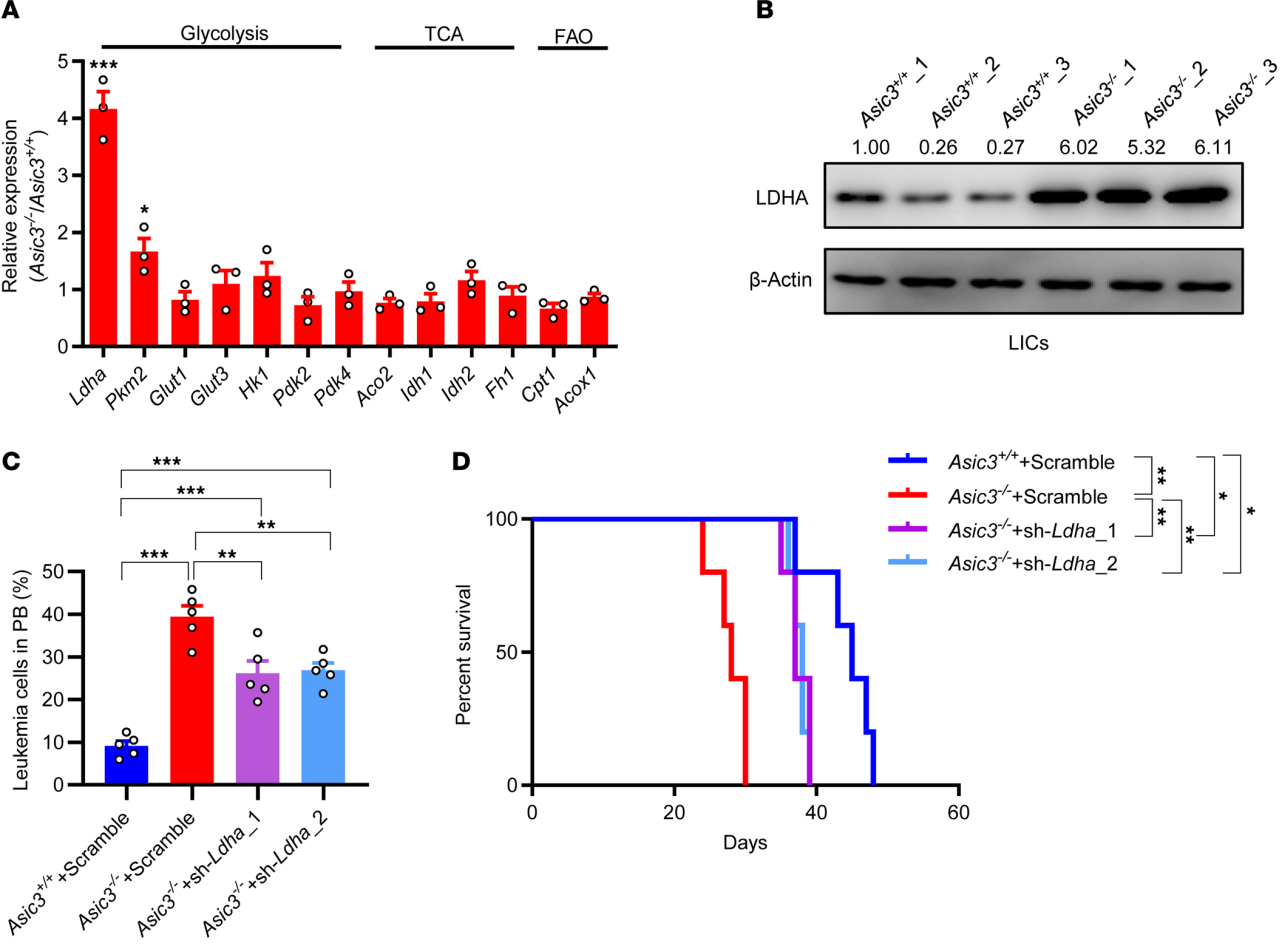
ASIC3 suppresses the glycolysis of LICs to delay the leukemogenesis. (**A**) mRNA levels of glycolysis, TCA, and fatty acid oxidation (FAO) genes in WT and *Asic3*-null LICs by qRT-PCR (*n* = 3). (**B**) Protein levels of LDHA were measured in WT and *Asic3*-null LICs, using immunoblotting. Ratios of LDHA/β-actin were quantified and normalized against *Asic3*^+/+^_1. (**C**) *Ldha* knockdown (sh-*Ldha*_1-2) in *Asic3*-null AML cells, followed by transplantation; GFP^+^ leukemia cell frequency in PB at 2 weeks (*n* = 5). (**D**) Recipient survival among WT, *Asic3*-null, and Ldha-knockdown *Asic3*-null groups (*n* = 5). Data are reported as mean ± SEM. Statistical analyses were conducted with Student 2-tailed unpaired *t* test (**A**), 1-way ANOVA with Tukey’s multiple comparison test (**C**), and log-rank test (**D**). *P < 0.05, **P < 0.01, ***P < 0.001.

We further examined mRNA expression levels of several *Meis1* downstream targets related to glycolysis (*Ldha*, *Pkm2,* and *Glut1*), oxidative phosphorylation determinants (*Aco2*, *Idh1*, *Idh2,* and *Fh1*), or fatty acid oxidation genes (*Cpt1* and *Acox1*) and found a 4.2-fold or 1.6-fold increase of *Ldha* or *Pkm2*, respectively, in *Asic3*-null LICs (Figure 6A). Protein levels of LDHA were remarkably elevated in *Asic3*-null LICs and total AML cells (Figure 6B and Supplemental Figure 9K), suggesting LDHA may serve as the main downstream molecule of MEIS1. This was evidenced by silencing of *Ldha* that could effectively reverse the phenotypes of the loss of function of ASIC3 (Figure 6, C and D, and Supplemental Figure 9, L–N). These data point to a unique connection between the acidic niche and glycolytic metabolism in LICs mediated by ASIC3.

### Acid-ASIC3 signaling inhibits the growth of human AML cell lines and LICs.

To unravel the roles of ASIC3 in human AML, we determined the mRNA levels of *ASIC3* in different subtypes of human AML cell lines by qRT-PCR and found most of them expressed ASIC3, including Kasumi-1 (M2), HL-60 (M3), THP-1 (M5), U937 (M5), and MV4-11 (M5) (Supplemental Figure 10A). To be consistent with the study in the murine AML model, ASIC3 was knocked down in THP-1, U937, MOLM-13, and Kasumi-1 cells by the validated shRNAs targeting *ASIC3* (sh-ASIC3_1-2), which drastically enhanced their proliferation in vitro (Supplemental Figure 10, B–I). More importantly, silencing *ASIC3* in THP-1 or U937 cells significantly increased the frequency of leukemia cells in the PB (Supplemental Figure 10, J and K) and markedly reduced the survival of NOD-SCID mice compared with their counterparts (Supplemental Figure 10, L and M). We overexpressed *ASIC3* in THP-1 and U937 AML cell lines and observed a significant reduction in cell growth (Supplemental Figure 10, N and O), supporting the potential antitumor role of ASIC3 in leukemogenesis. Knockdown of *ASIC3* in AML cell lines (THP-1, MOLM-13, and Kasumi-1) led to elevated protein levels of MEIS1, p-CREB, CAMK1, and STIM1 (Supplemental Figure 10, P–R). In addition, co-immunoprecipitation experiments demonstrated an interaction between ASIC3 and STIM1 in THP-1, U937, and MOLM-13 cells (Supplemental Figure 11A). *ASIC3*-knockdown THP-1 cells also had enhanced constitutive capacities of calcium influx (Supplemental Figure 11, B and C).

**Figure 7:**
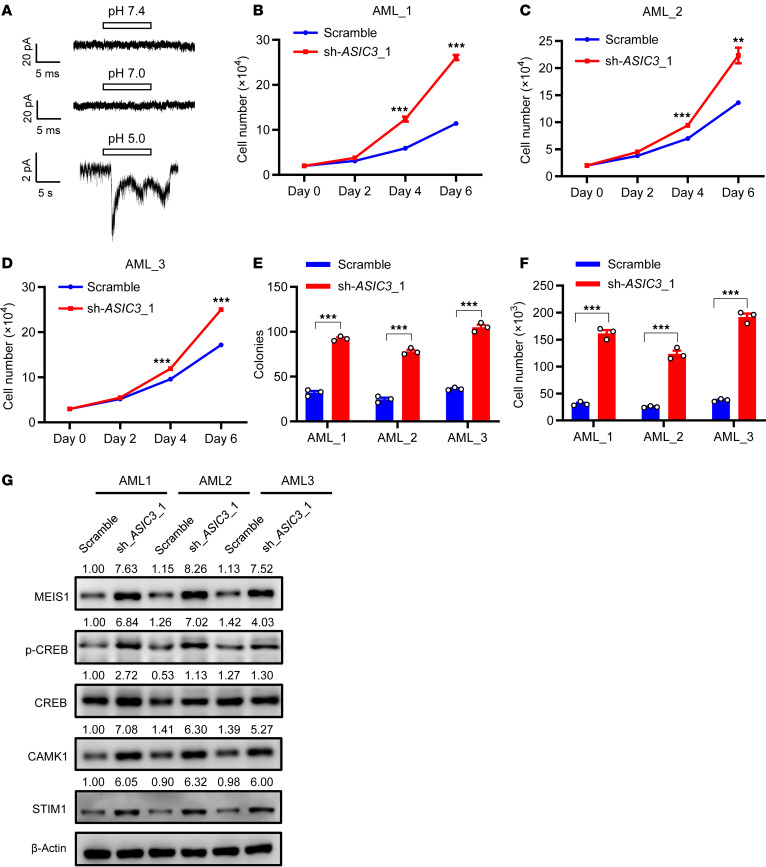
Acid-ASIC3 signaling inhibits the growth of human AML cell lines and LICs. (**A**) Whole-cell currents in human CD34^+^ LICs at pH 7.4, 7.0, and 5.0 (data are representative of 3 independent experiments). (**B**–**D**) Cell numbers were counted in *ASIC*3-knockdown (sh-*ASIC3*_1) CD34^+^ LICs, or scrambled LICs from 3 patients at the indicated days. (**E** and **F**) Colony-forming abilities were measured in *ASIC*3-knockdown CD34^+^ LICs or scrambled LICs from 3 patients (**E**); total cell numbers from respective colonies were counted as well (*n* = 3) (**F**). (**G**) Protein levels of MEIS1, p-CREB, CREB, CAMK1, and STIM1 were determined by immunoblot in samples from patients with *ASIC*3-knockdown (**B**–**F**). Ratio of these proteins to β-actin were quantified and normalized against AML1-Scramble. Data are reported as mean ± SEM. Statistical analyses were conducted with Student 2-tailed unpaired *t* test in **B**–**F**. **P <0.01, ***P <0.001.

To further test the functions of ASIC3 in human AML-LICs, Lin^–^CD34^+^CD38^–^CD90^–^CD45RA^+^ LICs and CD34^–^CD38^–^ leukemia cells were purified for the evaluation of *ASIC3* mRNA level by qRT-PCR, which showed that ASIC3 was also expressed in both cell populations, as well as their counterparts of human cord blood HSCs (Supplemental Figure 11, D–F). Similar to what is observed in mouse LICs, no obvious inward currents were elicited in CD34^+^-enriched human LICs at pH 7.4, pH 7.0, and pH 5.0, using whole-cell patch clamping (Figure 7A), which indicated acid-ASIC3 exerted a noncanonical effect on leukemia development. We then knocked down *ASIC3* in several human AML samples and demonstrated that the *ASIC3*-knockdown CD34^+^ LICs grew much faster compared with scrambled cells (Figure 7, B–D, and Supplemental Figure 11G) and gave rise to more colonies as well as total derived cell numbers (Figure 7, E and F). *ASIC3*-knockdown LICs also had notably enhanced levels of MEIS1, p-CREB, CREB, CAMK1, and STIM1 (Figure 7G) than did scrambled cells. In silico analyses indicated that certain subtypes of AML with abnormal karyotypes—inv (16) or t (15:17)—also had slightly reduced levels of *ASIC3* from the curated database (Supplemental Figure 11H), and *ASIC3* expression levels were positively correlated with the overall survival of patients with AML (Supplemental Figure 11I), which strongly suggests ASIC3, indeed, acts as a tumor suppressor in human AML.

A working model is depicted in Supplemental Figure 11J, showing LICs tend to reside in the endosteal niche with a relative neutral pH condition rather than in the vascular niche with a relatively lower level of pH. An acidic niche suppresses the self-renewal and glycolysis and promotes the differentiation of LICs through the noncanonical ASIC3-STIM1-CAMK1-MEIS1-LDHA pathway, which indicates ASIC3 may be ideal for targeting LICs.

## Discussion

The BM niche affords a special microenvironment to support LIC growth, escape from immune surveillance, and protect from chemotherapy or radiotherapy. Identification of key niche components and their downstream pathways may be beneficial for the development of novel strategies for leukemia treatment. In this study, we show that an acidic niche can activate ASIC3-mediated noncanonical signaling independent of its channel function in both mouse and human LICs to inhibit leukemia development. It is possible that other ligands exist, except for the hydrogen ions from the BM niche that can synergistically activate ASIC3 noncanonical pathways. Interestingly, Marra et al. (81) reported that lysophosphatidylcholine and arachidonic acid can serve as endogenous activators of ASIC3 in dorsal root ganglion neurons at physiological pH 7.4. Identification of additional ligands for ASIC3 may be beneficial for understanding ASIC3’s role in leukemogenesis and the development of strategies for leukemia treatment.

The canonical function of ASIC3 is as a proton-gated cation channel, where extracellular acidification triggers conformational changes leading to Na^+^ (and, to a lesser extent, Ca²^+^) influx through its transmembrane pore. This process primarily depends on the extracellular domain and the transmembrane segments. In contrast, the noncanonical mechanism identified in our study involves a physical interaction between the N-terminal intracellular domain of ASIC3 and STIM1, which modulates calcium signaling independently of ASIC3’s ion channel activity. This interaction likely influences STIM1’s ability to activate store-operated calcium entry pathways. These 2 mechanisms occur at different structural domains and may represent functionally distinct but potentially complementary roles of ASIC3 under different conditions. Importantly, in our AML model, the STIM1-ASIC3 N-terminal interaction appears to play a dominant role in regulating downstream calcium signaling and leukemia cell behavior, regardless of extracellular pH or ASIC3 conductance. Thus, we believe the noncanonical function does not conflict with, but rather expands, the physiological relevance of ASIC3 beyond its classical role as a pH sensor. We are currently investigating whether these 2 modes of action might cooperate or be differentially activated in response to environmental cues such as acidosis or ER calcium depletion.

ASIC3 expression is elevated in AML cells compared with normal hematopoietic cells; this may appear paradoxical given its inhibitory effect on AML progression. However, similar patterns have been observed for well-known tumor suppressors such as TP53, whose upregulation in leukemic cells is interpreted as a compensatory response to oncogenic stress, rather than a reflection of functional activity (82, 83). In this context, ASIC3 upregulation in AML may reflect an adaptive attempt by cells to counteract the progressively acidic BM microenvironment. Additionally, many genes exhibit opposing functions across malignancies depending on cellular context and microenvironment. For example, EZH2 acts as an oncogene in diffuse large B-cell lymphoma but displays tumor-suppressive properties in myeloid malignancies (84, 85). Similarly, ASIC3 may exert divergent effects in different malignancies depending on the cellular context, disease type, and microenvironmental conditions, such as extracellular pH. These observations underscore the need for further investigation into the canonical and noncanonical roles of ASIC3 across cancer types.

Many groups, including ours, have shown that LICs may use glycolysis as the main energy source in their hypoxic BM niches (59, 86, 87). We herein demonstrated that the deficiency of the acidic sensor of ASIC3 enhances the leukemogenesis through the metabolic switch from oxidative phosphorylation to glycolysis via an MEIS1-LDHA pathway. Our findings show a direct connection between an acidic microenvironment and glycolysis via ASICs, although it has been suggested that low pH may result in the switch of glycolysis to oxidative metabolism (28). Moreover, the acidic niche delivers an inhibitory signaling to suppress leukemogenesis, which may be different from the functions in many solid tumors as reported previously. We speculate there may exist an acidification-dependent effect on the cell fates of different tumors. In the present study, we also used a genetically encoded sensor to sensitively and specifically monitor the dynamics of glycolytic levels in live LICs, which may provide a unique angle and powerful tool to precisely evaluate the metabolic changes of LICs both in vitro and in vivo to dissect the impacts of different niche components on LIC metabolism. We are developing other metabolic sensors to explore whether acidic niche/ASICs or other acid-sensing molecules also have some effects on the metabolisms of other nutrients, including fat acids or amino acids.

In this study, we also engineered a pH biosensor, named pHluorin3, for the spatiotemporal monitoring of live-cell or in vivo pH. To our knowledge, this ultrasensitive, ratiometric pHluorin3 sensor presented the most intense brightness and the largest dynamic change among all the pHluorin versions in mammalian cells. Compared with pH measurements in BM niches using SNARF-1 dye, pHluorin3 allows for easy and robust detection of extracellular pH at single-cell resolution with high spatial accuracy. In our study, we used pHluorin3 to visualize and quantify extracellular pH in the BM microenvironment during AML progression. While this approach allowed us to capture spatial and temporal pH heterogeneity with high resolution, acidosis in the BM is often tightly coupled with hypoxic conditions, especially in poorly perfused niches such as the endosteal region. Therefore, combining pHluorin3 with oxygen sensors could provide a more comprehensive and integrated view of the metabolic and physiological landscape of the leukemic microenvironment. Such dual-parameter imaging would not only enhance our understanding of how pH and oxygen gradients coexist and interact in situ but also help clarify their individual and synergistic roles in regulating LIC behavior, metabolic reprogramming, and therapy resistance. Given the vital roles of pH in cell metabolism, we anticipate that the pHluorin3 sensor, together with other metabolite sensors such as the ATP sensor Ateam-1.03 (88), the NADH sensors Frex (89) and SoNar (90), the NADPH sensor iNap (91), and the glucose FLI Pglu-series (92, 93) can better visualize the landscape of cellular and subcellular metabolisms. In addition, the intense fluorescence and large dynamic range of pHluorin3 allow the high-throughput chemical screening in a microplate reader–based assay of candidate compounds or genes targeting cell metabolisms.

In summary, the acidic sensor of ASIC3 efficiently inhibits LIC activities and glycolysis through the STIM1-CAMK1-CREB-MEIS1-LDHA pathway in a noncanonical manner. Targeting ASIC3 may be an attractive way to eradicate LICs or other types of cancer stem cells.

## Methods

### Sex as a biological variable.

Our study examined male and female human and animals, and similar findings were reported for both sexes.

### Mice.

*Asic3* and *Asic1a* KO mice were generated as previously described (94, 95) and provided by John A. Wemmie, Margaret P. Price, and Michael J. Welsh at the University of Iowa (Iowa City, Iowa). C57BL/6 CD45.2 mice or NOD/SCID mice were purchased from the Shanghai SLAC Laboratory Animal Co. CD45.1 mice were provided by Jiang Zhu at Shanghai Jiao Tong University School of Medicine (Shanghai). All animal experiments were conducted in accordance with the guidelines approved by the Institutional Animal Care and Use Committee of Shanghai Jiao Tong University School of Medicine.

### Statistics.

Statistical analysis was performed using Prism, version 7.04 (GraphPad Software); SigmaPlot, version 12.5 (Grafiti); and SPSS software program, version 19.0 (IBM Corp.). Data are represented as mean ± SEM. Significance was calculated using unpaired 2-tailed Student’s *t* test for data with 2 groups; 1-way ANOVA with Tukey’s multiple comparison test or 2-way ANOVA with Šídák’s multiple comparison test was used for data with more than 2 groups. The survival in different groups was evaluated using the Kaplan-Meier method with a log-rank test. Experiments were biologically repeated at least 3 times unless otherwise indicated.

### Study approval.

Human samples were collected from the patients after diagnostic work in the Department of Hematology at the 1st People’s Hospital or Xinhua Hospital, Shanghai Jiao Tong University School of Medicine. Written informed consent was obtained from all the participants and the standard procedures were fully discussed and approved by the Ethics Committee for Medical Research (institutional review board) at Shanghai Jiao Tong University School of Medicine.

### Data availability.

All data supporting this study are included in the article or in the supplemental materials, with values for all data points in the graphs provided in the Supporting Data Values file. The raw bulk RNA-Seq data in this study have been deposited in the National Center for Biotechnology Information Gene Expression Omnibus database under accession number GSE102240.

All the other experimental details can be found in the supplemental material.

## Author contributions

HG, LW, CC, X Hao RT, XQ, Y Zhao, TLX, GQC, and JZ designed the experiments, performed the experiments, analyzed data and wrote the manuscript. XL, LL, TZ, YJ, JW, WGL, ZY, LX, Y Zhang, and X He performed the experiments. Y Yang, Y Yu, and DW provided reagents and helped with the experiments. The order of co–first authors was determined based on the relative magnitude of their contributions to the study.

## Funding support

- National Basic Research Program of China (grants 2024YFA1803500 and 2024YFA0917700).
- National Natural Science Foundation of China (grants 32030030, 82430007, 32030065, 32530064, 92457301, 82170175, 82370180, and 32371160).
- CAMS (Chinese Academy of Medical Sciences) Innovation Fund for Medical Sciences (grant, 2019-I2M-5-013).
- The Shanghai Songjiang District Science and Technology Research Project (grant 2023SJKJGG37).
- The Key Discipline Project of Shanghai Municipal Health Commission (grant 2024ZDXK0050).
- Fundamental and Interdisciplinary Disciplines Breakthrough Plan of the Ministry of Education of China.

## Supplementary Material

Supplemental data

Unedited blot and gel images

Supporting data values

## Version 1 12/15/2025

Electronic publication
