# Supplementary material for: Nonionotropic action of an acid-sensing ion channel inhibits leukemogenesis in the acidic bone marrow niche
Source: J Clin Invest. 2025 Dec 15;135(24):e189051. doi: 10.1172/JCI189051 (PMC12700551; doi:10.1172/JCI189051)
Supplement: Supplemental data [file jci-135-189051-s072.pdf]

Supplemental methods

Cell lines and primary patient AML cells

The acute myeloid leukemia (AML) cell lines THP-1 (ATCC), U937 (ATCC), MOLM-13 (DSMZ), Kasumi-1 (ATCC), HL-60 (ATCC), and MV4-11 (ATCC) were cultured in RPMI 1640 (Gibco, 11875093) supplemented with 10% fetal bovine serum (FBS) (ExCell Bio, FSP500) and 1% penicillin-streptomycin (Beyotime, C0222). HEK293T cells (Procell) were cultured in DMEM (Gibco, 11965092) supplemented with 10% FBS. Human primary AML cells were cultured in serum-free medium (StemCell Technologies, 09650) supplemented with 10 ng/ml human SCF (PeproTech, 300-07), 10 ng/mL human IL-3 (PeproTech, 200-03), and 10 ng/mL human IL-6 (PeproTech, 200-06).

For knockdown of *ASIC3* in AML cell lines and primary AML cells, plasmids pLKO.1-sh-*ASIC3*_1-GFP, pLKO.1-sh-*ASIC3*_2-GFP, or pLKO.1-Scramble-GFP were co-transfected with psPAX2 and pMD2.G packaging plasmids to produce virus supernatant. For overexpression of *ASIC3* in AML cell lines, plasmids pCDH-CMV-ASIC3-EF1α-copGFP or pCDH-CMV-EF1α-copGFP empty vector were co-transfected with psPAX2 and pMD2.G packaging plasmids to produce virus supernatant. THP-1, U937, MOLM-13, Kasumi-1, and primary AML cells were infected with lentiviruses through two rounds of spinoculation with 4 μg/ml polybrene (Yeasen, 40804ES76).

Mouse leukemia model

For mouse AML or B-cell acute lymphoid leukemia model, an MSCV-MLL-AF9-IRES-YFP plasmid, an MSCV-MLL-AF9-IRES-puro plasmid, an MigR1-AML1-ETO9a-IRES-GFP plasmid or an MSCV-N-myc-IRES-GFP plasmid was co-transfected with a pCL-ECO packaging plasmid (2:1) into HEK293T cells to produce retroviruses, followed by the infection of isolated *Asic3*$^{+/+}$ (WT) and *Asic3*$^{-/-}$ (*Asic3*-null) or *Asic1a*$^{+/+}$ (WT) and *Asic1a*$^{-/-}$ (*Asic1a*-null) Lin^{-} fetal liver cells by two rounds of spinoculation in the presence of 4μg/mL polybrene. Infected cells (3×10^5) were transplanted into lethally irradiated (10 Gy) C57BL/6 recipient mice by retro-orbital injection. Secondary transplantations were performed with purified YFP$^+$ or GFP$^+$ leukemia cells with

supporting cells (normal BM cells). For the limiting dilution analysis, the indicated numbers of WT and *Asic3*-null AML cells of primary recipients were injected into lethally irradiated recipients. The overall survival was recorded and LIC frequencies were calculated using the L-calc software provided by STEMCELL Technologies.

In vivo rescue assay

For rescue experiments, MSCV-mouse-ASIC3-IRES-GFP, MSCV-mouse-ASIC3^{E79C}-IRES-GFP, MSCV-mouse-ASIC3^{W280A}-IRES-GFP, MSCV-mouse-ASIC3^{Q443W}-IRES-GFP, MSCV-mouse-ASIC3^{G449A}-IRES-GFP or shRNA constructs (pLKO.1-sh-*Meis1*-GFP, pLKO.1-sh-*Stim1*-GFP, pLKO.1-sh-*Creb*-GFP, pLKO.1-sh-*Camk1*-GFP, and pLKO.1-sh-*Ldha*-GFP) were co-transfected with pCL-ECO (2:1) or psPAX2 and pMD2.G packaging plasmids (4:3:1) into HEK293T cells, respectively. The resulting virus supernatant was collected for the infection of WT and *Asic3*-null BM leukemia cells, followed by the injection into lethally irradiated (10Gy) recipients.

BM competitive transplantation

For the competitive reconstitution analysis, 5×10^5 CD45.2 wild-type or *Asic3*-null BM cells, along with 5×10^5 CD45.1 competitor cells were transplanted into lethally irradiated (10 Gy) CD45.1 recipients. Peripheral blood samples were collected and analyzed at 3 or 4, 8, and 16 weeks after transplantation. Hematological cell multilineage potential was assessed at 16 weeks. One million BM cells from primary recipients were used for the secondary transplantation.

Flow cytometry

The myeloid/lymphoid lineages and YFP$^+$Mac-1$^+$c-Kit$^+$ enriched LICs from WT and *Asic3*-null AML recipients were stained with anti-mouse Mac-1-APC (17-0112-82), Mac-1-PE (12-0112-85), Gr-1-PE (12-5931-83), CD3-APC (17-0032-82), B220-PE (12-0452-82), c-Kit-PE (12-1171-82), and c-Kit-APC (17-1171-82) monoclonal antibodies procured from eBioscience. YFP$^+$Lin$^-$CD127$^-$Sca-1$^-$c-Kit$^+$CD34$^+$CD16/32$^+$ immunophenotypic L-GMP cells were stained with anti-mouse CD3e-biotin (13-0031-85), B220-biotin (13-0452-85), Gr-1-biotin (13-5931-85), Ter119-biotin (13-5921-85), CD127-biotin (13-1271-82), Sca-1-biotin (13-5981-82), Streptavidin PerCP/Cyanine5.5 (45-4317-82), c-Kit-APC (17-1171-82), CD34-PE (12-0349-42), and CD16/32-eFluor 450 (48-0161-82) procured from eBioscience. The staining

procedure was performed as described previously (1). Human Lin$^-$CD34$^+$CD38$^-$ CD90$^+$CD45RA$^-$ LT-HSCs and Lin$^-$CD34$^+$CD38$^-$CD90$^-$CD45RA$^+$ LICs were identified by the antibodies against human Lin-Pacific Blue (348805), CD34-FITC (343504), CD38-PE/Cyanine7 (303516), CD90-PerCP/Cyanine5.5 (328118) and CD45RA-PE (304108) procured from BioLegend.

Chemicals

DNA polymerase prime star HS max and pfu DNA polymerase were obtained from TaKaRa. FastDigest Restriction endonucleases, T4 polynucleotide kinase, and T4 DNA ligase were purchased from Fermentas. Ionophores (nigericin and monensin), lysozyme, and RNase were provided by Sigma-Aldrich. Hydrogen peroxide was supplied by Calbiochem. Other reagents of analytical grade were obtained from local suppliers unless otherwise noted.

Mutagenesis and screening

We varied the amino acid mutations in the super ecliptic pHluorin from EGFP by using bridge PCR. The rationally designed mutation sites F46L, F64L, S72A, Q204H, and L220F were sequentially introduced into the super ecliptic pHluorin (2, 3). For the site-directed saturation mutagenesis, we incorporated base pairs NNN into primers on site 69, 146, 147, 148, 203, 204. The purified PCR products were phosphorylated, cyclized and transformed into Escherichia coli JM109 (DE3). Fluorescent colonies were cultured and induced by 1 mM isopropyl-β-D-thiogalactoside (IPTG) for 4 h at 37 °C. Cell pellets were diluted into phosphate buffered saline (PBS) with the absorbance value of 0.05 at 600 nm and measured the fluorescence to compare the brightness of the mutants. To measure the dynamic range, we lysed bacteria pellets in PBS containing 0.2 mg/ml lysozyme and 200 U/ml DNase I. The supernatants were diluted into buffers at pH 5.0 (100 mM citrate, 100 mM NaCl) and pH 9.0 (100 mM glycine, 100 mM NaCl). Fluorescence was monitored by the Synergy 2 Multi-Mode microplate reader equipped with an emission filter 528/20 and two excitation filters of 400/10 and 485/20. The colonies with intense fluorescence or significant response to pH were sequenced and then used to purify the recombinant probes for detailed characterization.

Protein expression and purification

Competent bacteria JM109 (DE3) or BL21plysS were transformed with the plasmids harboring the pHluorin variants. Single colonies were placed in 100 ml of LB medium with 0.1 mg/ml ampicillin and were cultured at 220 rpm for 10 h at 37 °C. The protein

was then induced with 1 mM IPTG at 37 °C for 4 h, followed by 18 °C for 12 h. Bacteria were collected and resuspended in binding buffer (30 mM Na_3PO_4, 500 mM NaCl, 10 mM imidazole, and 1 mM protease inhibitor PMSF, pH 7.4). After ultrasonication, bacteria lysate was clarified by centrifugation at 9600 g at 4 °C for 20 min and repeated twice. The supernatant was purified by the hexahistidine affinity chromatography (GE healthcare). Excess imidazole and salt in protein eluent were removed using a 6 ml Sephadex G-25 column (GE Healthcare) equilibrated with PBS. Protein concentration was determined using the BCA Protein Quantification Kit (Yeasen, 20201ES76).

Characterization of purified pHluorin3 *in vitro*

The purified pHluorin3 was diluted at 3 μM buffers with pH ranging from 4.0 to 10.0; the buffers contain 100 mM citrate (4.0–5.5), 100 mM MES (6.0–6.5), 100 mM HEPES (7.0–7.5), 100 mM TRIS (8.0–10.0), and 100 mM NaCl. Absorbance and fluorescence spectra were investigated using Model UV-2600A (UNICO) and Cary Eclipse spectrofluorimeter (Varian), respectively. The extinction coefficients and quantum yields of pHluorin3 in different pH buffer were measured at room temperature. The pH-dependent extinction coefficients were calculated according to the Beer-Lambert equation based on the absorbance spectra. We used the relative determination methods to measure the pH-dependent quantum yield measurements using EGFP (QY=0.60) at pH 7.5 as the reference standard (4). In the microplate experiments, the conditions of fluorescence measurement of recombinant protein were similar to those of screening. After background subtraction, the minimum and maximum values of excitation ratios ($R_{400/485}$) were normalized to 0 and 1 at pH 5.0 and 9.0, respectively. Three independent measurements were averaged and fitted by SigmaPlot to calculate pKa.

To determine the pHluorin3 specificity against other environmental disturbances, we prepared 0.1 μM pHluorin3 or iR-pHluorin in buffers containing oxidants, reductants, or different ion compositions at concentrations listed below: 0.5 mM H_2O_2 and 0.5 mM diamide; 10 mM GSH, 10 mM DTT; cations: 300 mM Na^+, 300 mM K^+, 10 mM Ca^{2+}, and 10 mM Mg^{2+}; and anions: 300 mM Cl^-, 10 mM HCO_3^-, 10 mM SO_4^{2-}, 10 mM PO_4^{3-}, and 10 mM NO_3^-. Sequential acidification and alkalization were conducted to investigate the response kinetics of pHluorin3 in solutions. After acquisition of fluorescence in the first 3 min, 1 mM HEPES buffer (pH 7.0), 10 mM MES (pH 6.0), 50 mM TRIS (pH 8.5), and 100 mM HEPES (pH 7.4) were successively added to the pHluorin3 probe with the interval of 5 min. The reversible fluorescence change was immediately detected by the microplate reader.

Mammalian cell expression and plasmid construction

The cDNA of pHluroin3 in the pRSETb vector was digested and ligated to the pcDNA3.1 vector using BamHI and HindIII. To exclude pHluorin3 from nucleus, we placed the tandem fused MAPKK signal peptide at the N-terminus (5). Mitochondrial pH reporter plasmid was constructed by fusing the duplicated mitochondrial signal peptide (30 amino acids) with the cytochrome c oxidase subunit VIII at the N-terminus (6).The pH sensor was cloned into the pDisplay vector (Invitrogen) to target pHluroin3 to the surface of mammalian cells. For nuclear targeting, we used the three-fold nuclear localization sequence, $(DPKKKRKV)_3$, at the C-terminus (7). The pH indicator without any targeting sequence was uniformly distributed in the cytosol and nuclei because of the large diffusion ability of the nuclear membrane pore. For the construction of stable cell line, we cloned pHluorin3 into the pLVX lentivirus plasmid by NheI and PmeI.

Cell culture and transfection

HeLa cells (cell bank of Chinese Academy of Sciences) and HEK293T cells were maintained in a humidified atmosphere of 95% air and 5% CO_2 and were cultured in DMEM supplemented with 10% FBS at 37 °C. For the transient transfection, about 40,000 HeLa cells were plated on a 4-well glass bottom dish (35 mm^2). The plasmids were transfected with Hieff Trans Liposomal Transfection Reagent (Yeasen, 40802ES) according to manufacturer's instructions and were imaged 24–36 h after the transfection.

Construction of stable cell lines expressing pHluorin3

To generate a stable cell line that expresses pHluorin3, we co-transfected of pLVX-pHluorin3 plasmid and two packaging plasmids pMD2.G and psPAX2 with the mass ratio of 4:1:3 into HEK293T using Hieff Trans; the culture medium was collected at 48 and 72 h. About 30% confluent of HeLa cells in the 6-well plate were then transduced by the supernatants supplied with 4 μg/ml polybrene and centrifuged at 1,800 rpm for 1 h. After 48 h, the cell lines were selected with 0.2 μg/ml puromycin, followed by FACS sorting.

Imaging with fluorescence microscope

In the cell imaging experiment, we used the high-performance Nikon Eclipse Ti-E automatic microscope with the Sutter Lambda XL as the light source. The digital image was captured by a Photometrics Evolve 512 EM-CCD. For the ratiometric imaging of pHluorin3 and iR-pHluorin, two excitation filters (387BP11 (semrock) and 482BP30

(semrock)) and one 535BP40 emission filter (semrock) were controlled by a Lambda 10-XL filter wheel (Shutter Instruments). The 12-bit depth images were captured using 640 × 480 format for all experiments. The background of images was subtracted in NIS-Element AR software (Nikon). These pictures were then submitted to image J for quantification and creation of ratiometric images by dividing the 387 nm picture by the 482 nm image pixel by pixel.

Calibration of intracellular and extracellular pH

Stably expressed pHluorin3 HeLa cells were re-seeded into 96-well plate with 20,000 cells per well. *In situ* titration of HeLa cells at room temperature was performed as reported with few modifications. Adhered cells were washed twice using 150 mM KCl and bathed in NaCl-free pH buffers with 150 mM KCl and 5 μM each of the ionophores, nigericin and monensin. About 3-5 min of balance, the excitation ratio of cytosolic pHluorin3 was stable and imaged by a Plan Apo 20 × 0.75 NA objective. The ratios of over 100 cells in each pH were collected and plotted as the standard curve. HeLa cells expressing the pHluorin3 sensor in different compartments were washed, incubated into HBSS buffer, and then imaged using the same sets in the permeability experiments. The proton concentrations in various subcellular organelles were calculated by fitting the excitation ratios of pHluorin3 to the standard curve.

For *in vitro* measurement of extracellular pH, THP-1 cells or MLL-AF9^{+} mouse AML cells expressing pHluorin3 were incubated in buffer solutions ranging from pH 4.0 to pH 10.0 or in culture medium, and imaged using a Nikon A1 confocal microscope under 405 nm and 488 nm excitation.

For *in vivo* detection of bone marrow niche pH in NOD-SCID mice transplanted with THP-1 cells, 5×10^{6} THP-1 cells expressing pHluorin3 were injected into untreated 6–8-week-old NOD-SCID mice. Two weeks post-transplantation, once more than 10% of circulating leukocytes in the peripheral blood were pHluorin3^{+}, calvarium samples were harvested, placed horizontally in a 35-mm glass-bottom dish containing either pH 4.0–10.0 buffer or PBS + 2% FBS, and imaged using a Nikon A1 confocal microscope. For bone marrow niche pH measurement in C57BL/6 mice transplanted with either lineage-negative normal bone marrow cells or MLL-AF9^{+} mouse AML cells expressing pHluorin3, 1×10^{6} pHluorin3^{+} lineage-negative normal BM cells or 1×10^{4} pHluorin3^{+} MLL-AF9^{+} AML cells (mixed with 2×10^{5} support cells) were transplanted into recipient mice conditioned with two doses of 3 Gy irradiation (administered 4 hours apart). At the indicated time points (at least 4 weeks post-transplantation), calvarium samples were harvested and placed horizontally in a 35-mm glass-bottom dish containing IMDM + 2% FBS, followed by imaging using a Nikon A1 confocal microscope. For the

detection of cortical bone region and trabecular bone region, fresh intact femurs from leukemic mice were partially decorticated using a microscalpel and fine abrasive paper to generate an optical window over both trabecular and cortical bone regions while preserving the underlying bone marrow integrity. Dual-excitation ratiometric pH imaging was performed using a two-photon microscope (Olympus FVMPE-RS) with excitation at 780 nm and 900 nm—corresponding to conventional 405 nm and 488 nm excitation for pHluorin3—with emission collected at 510 nm. Simultaneously, bone microarchitecture was visualized using excitation at 780 nm and emission at 450 nm. We defined the endosteal niche as cells in direct contact with the endosteal surface and located more than 10 μm (approximately one cell diameter) away from the nearest VE-cadherin^{+} sinusoidal vessel. Conversely, the vascular niche was defined as cells in direct contact with vascular endothelium and positioned more than 10 μm from the endosteal surface.

Fluorescence signals were visualized as pseudo-color representations of the 405 nm/488 nm excitation ratio and quantified using ImageJ software. The conversion of the fluorescence ratio (405 nm/488 nm) to pH values was based on the equation: pH = pKa + log((R - Rmin) / (Rmax - R)), where pKa is 7.3, R is the F405/F488 fluorescence ratio of the sample, and Rmin and Rmax represent the ratios obtained from cells incubated in pH 4 and pH 10 buffer, respectively.

Comparison of response of pDisplay-pHluorin3 and pCytosol-pHluroin3 to intracellular pH change

HEK293T cells were transfected with pDisplay-pHluorin3, pCytosol-pHluroin3, or control (cpYFP) plasmids. After 24 h, transfected cells were treated with/without 30 mM NH_4Cl (pH 7.4) or 10 mM Sodium acetate (pH 7.4), followed by the evaluation of the changes of the ratios of fluorescence with excitation at 405 and 488 nm using a Nikon A1 confocal microscope living cell imaging system.

Xenograft analysis

For the xenograft experiment, a total of $5x10^6$ *ASIC3*-knockdown THP-1 or U937 cells and scrambled cells were transplanted into 6-8-week-old female NOD/SCID recipient mice by retro-orbital injection. Percentages of leukemia cells in the peripheral blood were examined at indicated time point after transplantation, and the overall survival was evaluated as well.

RNA-sequencing and quantitative RT-PCR

WT and *Asic3*-null $YFP^+Mac\text{-}1^+c\text{-}Kit^+$ LICs were purified by flow cytometry for the extraction of total RNA and subjected to RNA-sequencing. A Gene ontology enrichment analysis was performed by a Bioconductor package "topGo". A KEGG pathway enrichment analysis was conducted by a Bioconductor package "GSEABase" (http://www.r-project.org). For quantitative RT-PCR, first strand cDNA was reverse transcribed using AMV reverse transcriptase (TakaRa). PCR reactions were performed according to the manufacturer's protocol. In brief, 10 μL reactions with FastStart Universal SYBR Green Master (ROX), primers and cDNA were used for the analysis of mRNA expression levels with the Applied Biosystems 7900HT. The mRNA levels were normalized to the level of β-actin RNA transcripts. The primer sequences used are shown in Supplemental Table 4.

Colony forming unit assay and hematoxylin and eosin staining

Ten thousand leukemia cells of primary recipient mice were seeded in methylcellulose medium (Stem Cell Technologies, M3534) according to the manufacturer's instructions. The numbers of colonies were counted 6 days after culture, followed by re-plating with the same numbers of primarily plated leukemia cells. For human primary AML cells, 3×10^3 AML cells were seeded into methylcellulose-based medium (StemCell Technologies, H4535). Colonies and derived cell numbers were counted 6 days after plating. Liver and spleen tissues of *Asic3*$^{+/+}$ and *Asic3*$^{-/-}$ leukemic mice were fixed in 4% paraformaldehyde and embedded in paraffin. Sections were stained with hematoxylin and esosin for the analysis of the infiltration of leukemia cells.

Transfection of CHO cells

Transient transfection of CHO cells (cell bank of Chinese Academy of Sciences) was performed using HilyMax liposome transfection reagent (Dojindo). For 35-mm dish with 80% cell density, 2 μg of the indicated plasmid (pEGFP-C3-ASIC3, pEGFP-C3-ASIC3^{E79C}, pEGFP-C3-ASIC^{W280A}, pEGFP-C3-ASIC3^{Q443W}, pEGFP-C3-ASIC3^{G449A}) was added into 120 μL Opti-MEM (Gibco), followed by the mixing with 10 μL HilyMax solution. The mixture solution was transferred into the dish after incubating at room temperature for 15 minutes. The electrophysiological currents mediated by ASIC3 or its mutants in transfected cells were recorded 24 h after transfection. Membrane protein of transfected cells was also purified for the validation of their expression levels.

Electrophysiology

The electrophysiological recordings were performed using the conventional whole-cell configuration under voltage clamp as described previously (8). Patch pipettes were

pulled from glass capillaries on a two-stage puller (PC-10, Narishige). The resistance between the recording electrode filled with pipette solution and the reference electrode was 5-10 MΩ. Membrane currents were measured using a patch clamp amplifier (Axon patch 200B, Molecular Devices) and were filtered at 2 kHz using low-pass Bessel filter. All currents were sampled and analyzed using a Digidata1440 interface and a computer running the Clampex and Clamp-fit 10.0 software (Molecular Devices).

Membrane protein purification

Membrane protein were purified with CHO cells as described previously (9). Twenty-four hours after transfection with ASIC3 or its mutants, cells were washed three times with ice-cold PBS (pH 8.0), followed by the addition of 2 ml of 0.25 mg/ml Sulfo-NHS-LC-Biotin (Thermo Scientific) in the PBS (pH 8.0) to each 6-cm dish and incubated at 4°C for 30 min with gentle rocking. Then cells were washed once with PBS plus 0.1 M glycine to quench and remove excess biotin reagent. Total proteins were extracted from cell lysis and incubated overnight at 4°C with High Capacity NeutrAvidin™ Agarose (Thermo Scientific). The beads were washed five times with the PBS and mixed with SDS sample buffer for the subsequent immunoblot analysis.

Immunoblot analysis and Immunofluorescence staining

For immunoblot analysis, whole cell lysates were electrophoresed on 10% sodium dodecyl sulfate-polyacrylamide gels and transferred to nitrocellulose membrane (Millipore). The membranes were incubated with primary antibodies overnight at 4°C, followed by incubation with appropriate horseradish peroxidase-conjugated secondary antibodies. The following primary antibodies were used: anti-MEIS1 (Abcam, ab19867), anti-CREB (Abways, CY5426), anti-phospho-CREB (CST, 9198), anti-CAMK1 (Abcam, ab68234), anti-STIM1 (CST, 5668), anti-LDHA (Proteintech, 21799-1-AP), anti-PKM2 (CST, 4053), anti-GFP (Santa Cruz Biotechnology, sc-9996), anti-Flag (Sigma-Aldrich, A8592), anti-Strep Ⅱ（GenScript, A02342) and anti-β-actin (MBL, PM053). For immunofluorescence staining, sorted $YFP^{+}Mac\text{-}1^{+}c\text{-}Kit^{+}$ LICs were fixed by pre-chilled 4% paraformaldehyde containing 4% sucrose. Cells were treated with 0.3% Triton X-100, blocked with 3% BSA in PBS for 60 min, and followed by incubation with anti-STIM1 antibody (CST, 5668) at 4 ℃ overnight. Cells were incubated with fluorochrome-conjugated secondary antibodies (Thermo Scientific, A-21428) 1 h at room temperature. DAPI was included in all staining to evaluate nuclear morphology. Microscopy was performed using a Nikon A1 confocal microscope.

Co-immunoprecipitation

CMV5.1-mosue-STIM1-Flag and pLVX-mouse-ASIC3-Strep Ⅱ, or pLVX-mouse-ASIC3$^{N\text{-terminal-deletion}}$-Strep Ⅱ (lacking the first 43 amino acids at the N-terminus), or pLVX-mouse-ASIC3$^{C\text{-terminal-deletion}}$-Strep Ⅱ (lacking the last 70 amino acids at the C-terminus) plasmids were transfected into HEK293T cells. After 48 h, transfected cells were collected for lysis using weak RIPA buffer containing protease inhibitor and PMSF. After lysis for 30 min at 4 ℃ while slowly rotating, the lysates were sonicated and then centrifuged at 13, 000 g for 15 min. The supernatant was incubated with Flag M2 beads (Sigma-Aldrich) or Strep Ⅱ beads (IBA Lifesciences) overnight at 4 ℃. Then beads were washed three times with PBS containing 0.5% NP-40, and mixed with SDS buffer and subjected to the subsequent immunoblot analysis.

Lentiviruses were generated using the pCDH-human-ASIC3(3×Flag)-EF1-GFP or pCDH-MCS-EF1-GFP plasmids and used to establish stable *ASIC3*-overexpressing or control THP-1, U937, and MOLM-13 cell lines. Cells were harvested by centrifugation and lysed in RIPA buffer supplemented with protease inhibitor cocktail and PMSF on ice for 30 minutes. Lysates were then sonicated and clarified by centrifugation at 13,000 g for 15 minutes at 4 °C. The resulting supernatants were incubated with anti-Flag M2 affinity beads overnight at 4 °C. Beads were washed three times with PBS containing 0.5% NP-40, and bound proteins were eluted in SDS loading buffer for subsequent immunoblotting analysis.

Luciferase reporter assays

The luciferase reporter vector pGL4.27 containing the *Stim1* promoter was constructed for identification of transcriptional activation of *Stim1* by MEIS1. Indicated doses of pLVX-MEIS1-Strep Ⅱ (or negative control vector) plasmid along with pGL4.27-Stim1 promoter vector were co-transfected into HEK293T cells. Luciferase activities were measured according to the manufacturer's instructions (Promega, E1910) by using a luciferase reporter system (GloMax®Multi Instrument) 24 h after transfection.

Chromatin immunoprecipitation (ChIP) assays

ChIP assays were performed using the ChIP Assay Kit (Beyotime, P2078). Briefly, HEK293T cells were transfected with pGL4.27-Stim1 and pLVX-MEIS1-Strep Ⅱ plasmids and then crosslinked with 1% formaldehyde (Sigma--Aldrich) at 37 °C for 10 min. Pre-cleared DNA was used for immunoprecipitation with 4 mL of anti-Strep Ⅱ antibody (GenScript, A02342) or rabbit IgG isotype control (CST, 3900) at 4°C overnight. For the sample input, 1% of the sonicated pre-cleared DNA was purified at

the same time with the precipitated immune complex. The ChIP samples were purified by the Gel and PCR-clean up Kit (Necleospin). The MEIS1-binding sequence was amplified by the semi-quantitative PCR using primers specific for *Stim1* promoter region as listed in Supplemental Table 4.

Calcium imaging

Intracellular Ca^{2+} levels were measured with Fura-2 AM as described previously (10). Briefly, WT and *Asic3*-null BM leukemia cells, $YFP^+Mac\text{-}1^+c\text{-}Kit^+$LICs, *ASIC3*-knockdown-THP-1 cells and their control cells were seeded on the poly-D-lysine pre-treated glass slide, followed by the incubation with 2 μM Fura-2 AM for 45 min. Cells were then maintained in Fura-2 AM free solution for another 45 min. Cells were first subjected to the Ca^{2+}-free solution (pH 7.4) for 2 min, which was then changed to the solution with 1 mM Ca^{2+} (pH 7.4 or 7.0) for 2-3 min, and followed by incubating in Ca^{2+}-free solution (pH 7.4). For pharmacological blockade of Orai1-mediated Ca^{2+} entry, 10 μM Synta-66 (MedChemExpress, HY-111325) was added into 1 mM Ca^{2+}. The ratio of fluorescence with excitation at 340 (F340) and 380nm (F380) was recorded every 2 sec and shown.

Sodium imaging

Intracellular Na^+ signals in LIC were monitored using the fluorescent sodium indicator CoroNa Green AM (Thermo Fisher, C36676). Cells were loaded with 2 μM CoroNa Green AM and 0.02% Pluronic F-127 in standard extracellular solution for 30 min at 37 °C/5% CO_2, followed by 30 min de-esterification in dye-free solution and attached to glass-bottom dishes pre-coated with 0.1 mg/mL poly-L-lysine. Imaging was performed on an inverted confocal microscope with 488 nm excitation and 515–565 nm emission collection. All recordings were conducted at 25°C with continuous perfusion (1 mL/min).

Cell membrane potential measurement

Resting cell membrane potential (RMP) measurements were performed on LIC using whole-cell patch-clamp electrophysiology at room temperature (22–25 °C). Cells were suspended in culture and allowed to attach for 1 hour to glass-bottom dishes pre-coated with 0.1 mg/mL poly-L-lysine. The extracellular solution contained (in mM): 150 NaCl, 5 KCl, 10 Glucose, 10 HEPES, 2 $CaCl_2 \cdot 2H_2O$, and 1 $MgCl_2 \cdot 6H_2O$ (pH adjusted to 7.4 with NaOH). The pipette internal solution contained (in mM): 120 KCl, 30 NaCl, 1 $MgCl_2 \cdot 6H_2O$, 0.5 $CaCl_2 \cdot 2H_2O$, 5 EGTA, 2 Mg-ATP, and 10 HEPES (pH adjusted to

7.4 with NaOH). RMP recordings were conducted in current-clamp mode (I = 0) with cells stabilized for 3 min before recording continuous 60 sec traces.

Glycolytic levels measurement in living leukemia cells

The glycolytic levels were determined using a genetically encoded intracellular NADH sensor, SoNar, as described previously (1). Briefly, SoNar was stably expressed in WT or *Asic3*-null BM leukemia cells. Indicated cells were further seeded on the poly-D-lysine pre-treated glass slide, followed by the measurement of cytoplasmic NADH levels as displayed by the ratio of fluorescence with excitation at 405 and 561 nm with/without pyruvate (1 mM) and oxamate (5 mM) stimulation, or under conditions with low pH (7.0) using a Nikon A1 confocal microscope living cell imaging system.

Metabolic assay

The ATP content was measured using an ATP Bioluminescence Assay Kit HS Ⅱ (Sigma-Aldrich) according to the manufacturer's instruction. Oxygen consumption and lactate generation were measured using a Seahorse XF96 extracellular flux analyzer as previously described (11). Briefly, three replicate wells of $3x10^5$ WT and *Asic3*-null BM leukemia cells were seeded on 96-well plates coated with BD Cell-Tak (BD Biosciences) in unbuffered DMEM and incubated at 37 ℃ for pH stabilization. Analyses were performed both at basal conditions and after injection of oligomycin (0.5 μM for OCR or 2 μM for ECAR), FCCP (2 μM), antimycin A (0.5 μM) / rotenone (0.5 μM), glucose (10 mM) and 2-DG (100 mM). For extracellular lactate detection, culture medium from each well was collected at 2 h and 4 h and then 20 μL medium mixed with 180 μL of assay buffer (PBS pH 7.4; 0.1 % BSA; 500 μM NAD^+; 0.5 U LDH; 0.2 U diaphorase; 10 μM resazurin) was added to a 96-well plate. Changes in fluorescence were measured every 30 s for 15 min at 37 °C by a Synergy 2 Multi-Mode Microplate Reader with excitation filter 540 BP 25 nm and emission filter 590 BP 35 nm at 37 °C. Calibration experiments were performed with 20 μL lactate standards (0, 10, 20, 40, 60, 100 and 200 μM/well). All the samples were diluted to fit within the range of standard curve and run in triplicate. To analyze the mitochondrial DNA copy numbers, total genomic DNA was extracted from the indicated cells. The relative copy numbers of mtDNA were determined by comparing the copies of the mitochondrial-specific mt-ND4 gene with that of the nuclear B2m gene. The primer sequences used are shown in Supplemental Table 4.

Homing assay

A total number of 6×10^6 WT and *Asic3*-null BM leukemia cells of primary recipient mice were labeled with 5-(and -6) carboxyfluoresceinsuccinimidylester (CFSE) and injected into lethally irradiated mice. Total CFSE$^+$ cells were measured in the bone marrow, spleens and livers 16 h after transplantation by flow cytometric analysis. When CFSE$^+$Mac-1$^+$c-Kit$^+$LICs were analyzed, BM cells were stained with Mac-1-PE and c-Kit-APC before analysis.

Acid or peptide treatment

For acid and peptide treatment *in vitro*, WT and *Asic3*-null AML cells were cultured in basic medium (Stemcell Technologies, 09650) supplemented with 10 ng/mL of SCF (PeproTech, 250-03), 10 ng/mL of IL-3 (PeproTech, 213-13), and 10 ng/mL of IL-6 (PeproTech, 216-16) or seeded in methylcellulose medium (IMDM, FBS, 2-Mercaptoethanol, BSA, methyl cellulose, 10 ng/mL of SCF, 10 ng/mL of IL-3 and 10 ng/mL of IL-6). The indicated pH of medium was adjusted using HEPES buffer. For detection of MEIS1 protein level upon conditions slightly more acidic than pH 7.4 (pH 7.0), AML cells were harvested 18 h post stimulation. For peptide treatment, AML cells were treated with 10 μM NP-1 (GRKKRRQRRRCMKPPSGLEEAQRRQASDIRVFA, 1-22 aa), NP-2 (GRKKRRQRRRCNSCTMHGLGHIFGPGGLTLRR, 23-43 aa) produced according to the N-terminal sequence of ASIC3, or control (GRKKRRQRRRC) peptides for 6 h, followed by subjection to the calcium imaging, immunoblot analysis, in vitro culture and colony forming unit assay.

In silico analysis for clinical data

To analyze *ASIC3* expression in patients with AML and healthy BM, we extracted data from the BloodSpot database (12). To investigate the association between *ASIC3* expression levels and the overall survival of AML patients, we analyzed the GSE8970 dataset using the KM-plotter database (13-15).

References

1. Hao X, et al. Metabolic Imaging Reveals a Unique Preference of Symmetric Cell Division and Homing of Leukemia-Initiating Cells in an Endosteal Niche. *Cell metabolism.* 2019;29(4):950-965.e956.
2. Nagai T, et al. A variant of yellow fluorescent protein with fast and efficient maturation for cell-biological applications. *Nature biotechnology.* 2002;20(1):87-90.
3. Griesbeck O, et al. Reducing the environmental sensitivity of yellow fluorescent protein. Mechanism and applications. *Journal of Biological Chemistry.* 2001;276(31):29188-29194.
4. Patterson GH, et al. Use of the green fluorescent protein and its mutants in quantitative fluorescence microscopy. *Biophysical journal.* 1997;73(5):2782-2790.

5. Fukuda M, et al. A novel regulatory mechanism in the mitogen-activated protein (MAP) kinase cascade. Role of nuclear export signal of MAP kinase kinase. *The Journal of biological chemistry.* 1997;272(51):32642-32648.
6. Imamura H, et al. Visualization of ATP levels inside single living cells with fluorescence resonance energy transfer-based genetically encoded indicators. *Proceedings of the National Academy of Sciences of the United States of America.* 2009;106(37):15651-15656.
7. Zhao Y, et al. Genetically encoded fluorescent sensors for intracellular NADH detection. *Cell metabolism.* 2011;14(4):555-566.
8. Yu Y, et al. A nonproton ligand sensor in the acid-sensing ion channel. *Neuron.* 2010;68(1):61-72.
9. Mammen AL, et al. Redistribution and stabilization of cell surface glutamate receptors during synapse formation. *The Journal of neuroscience : the official journal of the Society for Neuroscience.* 1997;17(19):7351-7358.
10. Wang YZ, et al. Tissue acidosis induces neuronal necroptosis via ASIC1a channel independent of its ionic conduction. *Elife.* 2015;4.
11. Kocabas F, et al. Hypoxic metabolism in human hematopoietic stem cells. *Cell & bioscience.* 2015;5:39.
12. Gíslason MH, et al. BloodSpot 3.0: a database of gene and protein expression data in normal and malignant haematopoiesis. *Nucleic Acids Res.* 2024;52(D1):D1138-d1142.
13. Raponi M, et al. A 2-gene classifier for predicting response to the farnesyltransferase inhibitor tipifarnib in acute myeloid leukemia. *Blood.* 2008;111(5):2589-2596.
14. Győrffy B. Integrated analysis of public datasets for the discovery and validation of survival-associated genes in solid tumors. *Innovation (Camb).* 2024;5(3):100625.
15. Győrffy B. Transcriptome-level discovery of survival-associated biomarkers and therapy targets in non-small-cell lung cancer. *Br J Pharmacol.* 2024;181(3):362-374.

Supplemental Figure 1

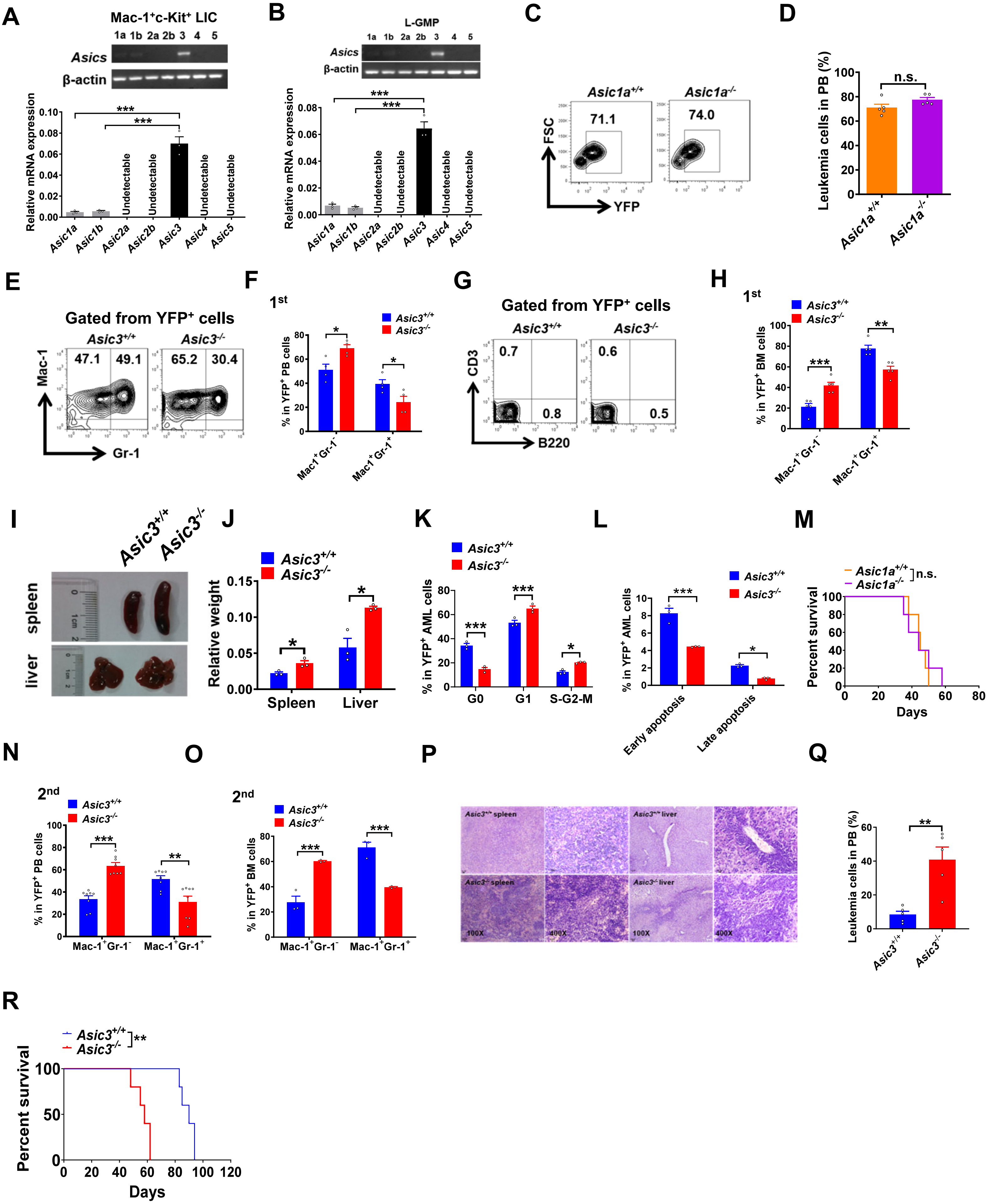

Supplemental Figure 1. (**A**) mRNA levels of *Asic1-5* in $YFP^+Mac\text{-}1^+c\text{-}Kit^+$LICs by qRT-PCR (n = 3); electrophoresis image of RT-PCR product (top). (**B**) mRNA expression levels of *Asic1-5* were determined in L-GMP cells by qRT-PCR (bottom, n = 3); electrophoresis image of RT-PCR product (top). (**C**) Representative flow cytometric analysis for the frequencies of wild-type (WT) and *Asic1a*-null leukemia cells (YFP^+) in the PB 3 weeks after primary transplantation. (**D**) Quantitative data in panel **C** (n = 5). (**E**-**G**) Representative flow cytometric images of lineage analysis with myeloid cell markers (Mac-1/Gr-1, E) and lymphoid cell markers (CD3/B220, G) of YFP^+ leukemia cells in YFP^+ PB cells 5 weeks after primary transplantation. Quantification data are shown in panel F (n = 4). (**H**) Frequencies of $YFP^+Mac\text{-}1^+Gr\text{-}1^+$ and $YFP^+Mac\text{-}1^+Gr\text{-}1^-$ leukemia cells in the YFP^+ BM AML cells of recipients 5 weeks after primary transplantation (n = 5). (**I** and **J**) Images and relative weights of spleens and livers from recipients 5 weeks post-transplantation (n = 3). (**K**) Cell cycle analysis of WT and *Asic3*-null YFP^+ AML cells using Ki-67/DAPI staining (n = 3). (**L**) Apoptosis analysis of WT and *Asic3*-null YFP^+ AML cells using Annexin V/PI staining (n = 3). (**M**) Survival was analyzed in primary recipients receiving MLL-AF9$^+$ WT and *Asic1a*-null Lin^- cells (n = 5). (**N**) Frequencies of $YFP^+Mac\text{-}1^+Gr\text{-}1^+$ and $YFP^+Mac\text{-}1^+Gr\text{-}1^-$ leukemia cells in the YFP^+ PB cells of recipients 2 weeks after secondary transplantation (n = 8). (**O**) Frequencies of $YFP^+Mac\text{-}1^+Gr1^+$ and $YFP^+Mac\text{-}1^+Gr1^-$ leukemia cells in the YFP^+ BM cells of recipients 2 weeks after secondary transplantation (n = 3). (**P**) Histological hematoxylin/eosin staining of the livers and spleens of recipients 2 weeks after secondary transplantation. (**Q**) The frequencies of WT and *Asic3*-null AML1-ETO9a$^+$ leukemia cells (GFP^+) in PB after primary transplantation (n = 5). (**R**) Survival was analyzed in primary recipients receiving AML1-ETO9a$^+$ WT and *Asic3*-null Lin^- cells (n = 5). Data are represented mean ± SEM. *P*-values determined by 1-way ANOVA with Tukey's multiple comparison test (A and B), Student 2-tailed unpaired *t* test (D, J, and Q), 2-way ANOVA with Sidak's multiple comparison test (F, H, K, L, N, and O), and log-rank test (M and R). *, $P<0.05$; **, $P<0.01$; ***, $P<0.001$. n.s., not significant.

Supplemental Figure 2

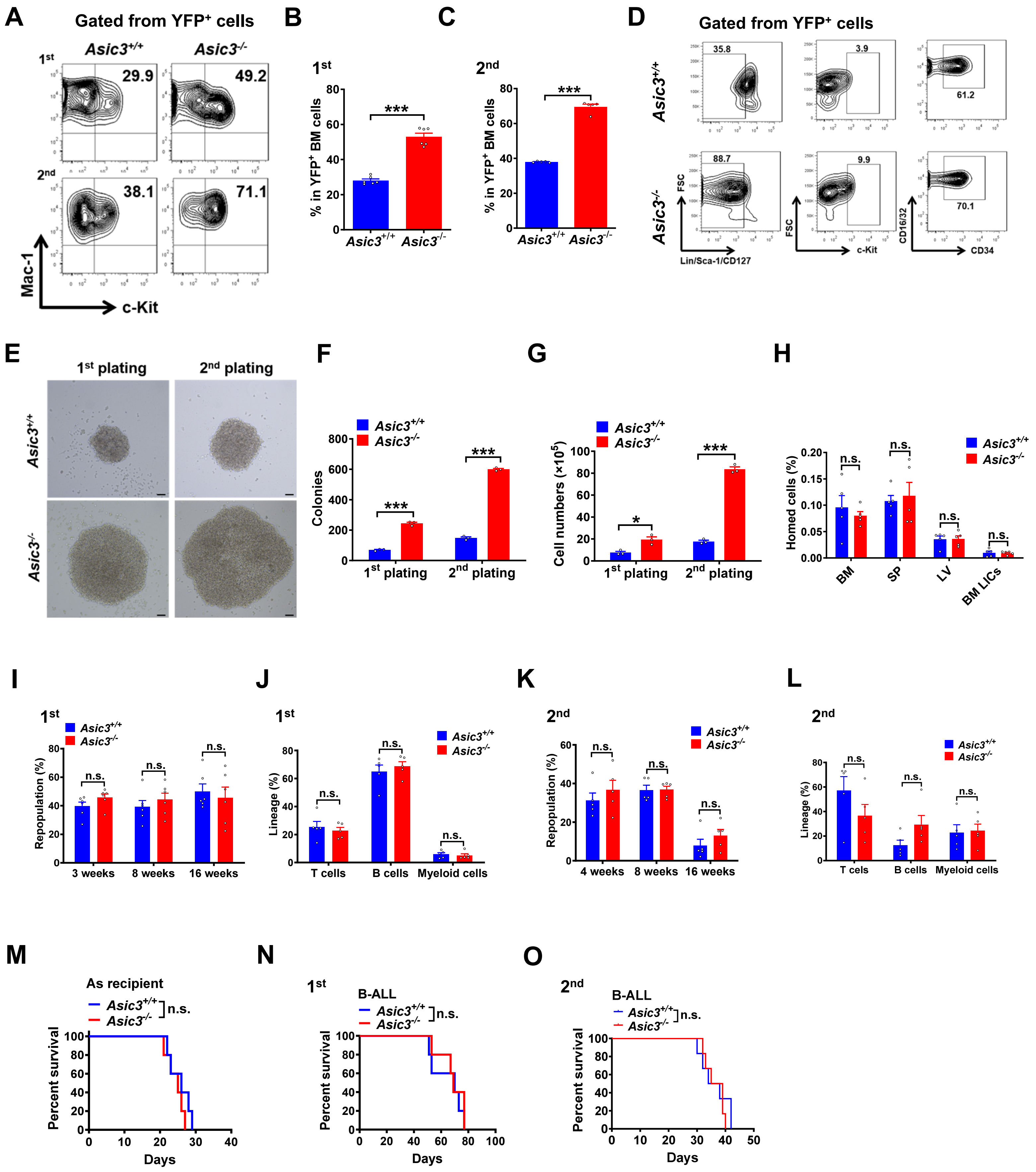

Supplemental Figure 2. (**A**-**C**) Representative flow cytometric analysis of the $YFP^+Mac\text{-}1^+c\text{-}Kit^+$ LICs in the YFP^+ BM cells of primary (1st, upper panel) and secondary (2nd, bottom panel) recipients (A), and the quantification results are shown (B and C, n = 5-6). (**D**) Representative flow cytometric analysis for WT and *Asic3*-null $YFP^+Lin^-CD127^-Sca\text{-}1^-c\text{-}Kit^+CD34^+CD16/32^+$ bone marrow (BM) cells (L-GMP cells) in the YFP^+ BM cells from recipients 5 weeks after primary transplantation. (**E**-**G**) Representative colonies from WT and *Asic3*-null YFP^+ leukemia cells in 1st and 2nd platings (E, scale bar: 50 μm), colony numbers (F), and total cell counts (G) (n = 3). (**H**) Quantification data of WT and *Asic3*-null YFP^+ cells homed to the BM and spleens 16 h after transplantation (n = 5). (**I**-**L**) The frequencies of WT and *Asic3*-null donor cells were assessed at the indicated time points following primary (I, n = 6) and secondary (K, n = 5) competitive BM transplantation. Multilineage potential of donor cells in the recipient mice transplanted with WT and *Asic3*-null normal BM cells 16 weeks post-primary (J, n = 5) and secondary (L, n = 5) transplantation. (**M**) Survival was analyzed in WT and *Asic3*-null mice receiving WT YFP^+ leukemia cells from primary recipients (n = 5). (**N** and **O**) Survival data for recipient mice receiving WT or *Asic3*-null $N\text{-}Myc^+$ BM cells upon the 1st (N, n = 5) and 2nd (O, n = 6). Data are represented mean ± SEM. *P*-values determined by Student 2-tailed unpaired *t* test (B, C, F, G, H, I, and K), 2-way ANOVA with Sidak's multiple comparison test (J and L), and log-rank test (M-O). *, $P < 0.05$; ***, $P < 0.001$. n.s., not significant.

Supplemental Figure 3

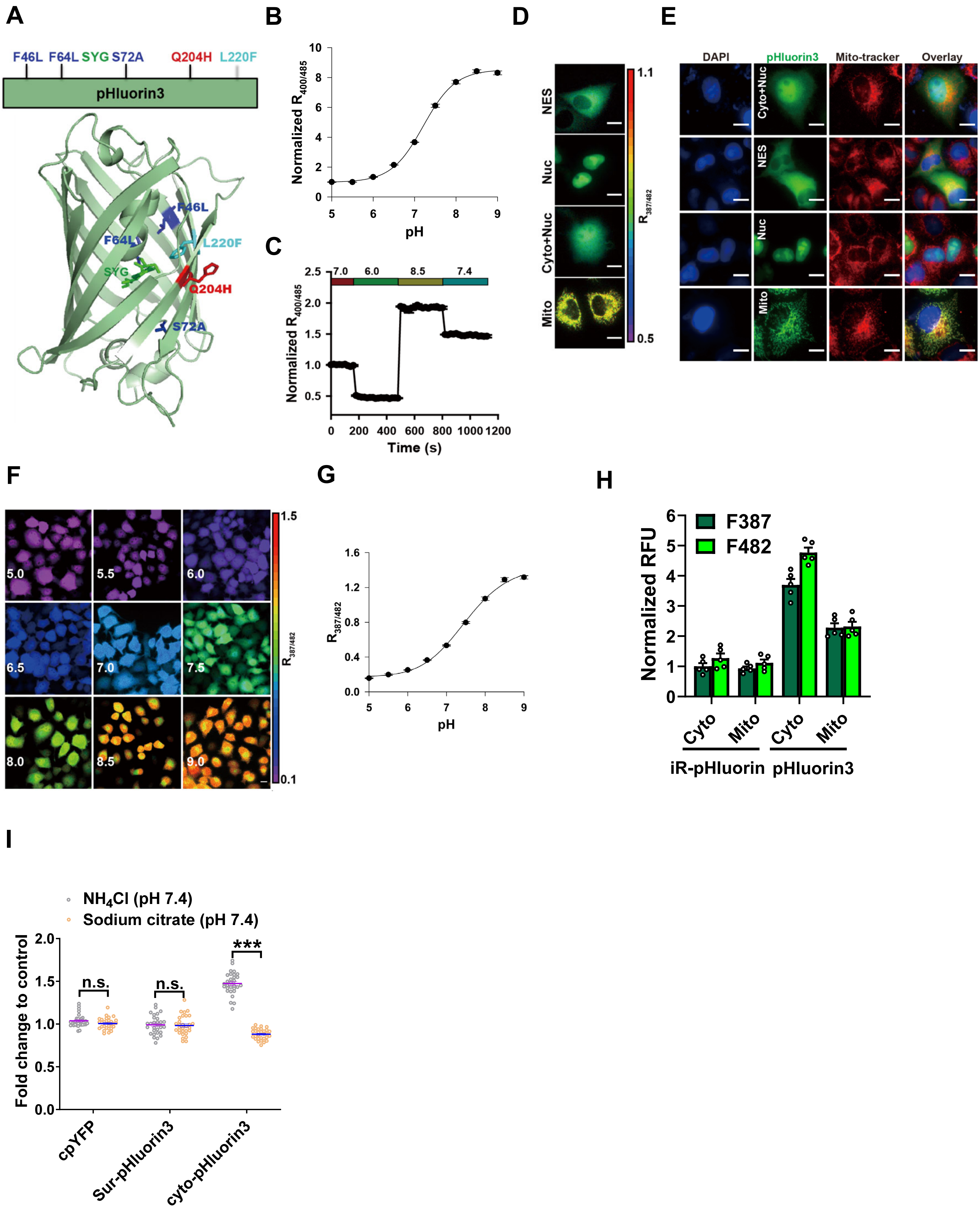

Supplemental Figure 3. (**A**) pHluorin3 design with mutations F46L, F64L, S72A, Q204H, and L220F; chromophore SYG shown as green star; mutation sites highlighted on pHluorin3 structure (PDB:1YHG). (**B**) Intensity ratios (R400/485) normalized to pH 5 (n = 3). (**C**) Response kinetics of pHluorin3 to sequential acidification/alkalization. (**D**) Ratiometric fluorescence images of HeLa cells expressing pHluorin3 in nucleus-excluded (NES), nucleus (Nuc), cytosol/nucleus (Cyto+Nuc), mitochondria (Mito). Scale bar, 10 μm. (**E**) HeLa cells expressing pHluorin3 sensors (green) in different subcellular organelles were stained with by DAPI (nucleus, blue) and Mito-tracker 580 (red). Scale bar, 10 μm. (**F**) Ratiometric fluorescence images of HeLa cells expressing pHluorin3 exposed to pH buffers. Scale bar, 10 μm. (**G**) Fluorescence ratio of Hela cells expressing pHluorin3 in response to the indicated pH buffer (n = 3). (**H**) Comparison of fluorescence intensity of pHluorin3 and iR-pHluorin in cytosol (Cyto) and mitochondria (Mito) of HeLa cells (n = 5). (**I**) Response of 293T cells expressing cpYFP, surface- or cyto-pHluorin3 to NH_4Cl or sodium citrate, normalized to PBS (n = 30). Data are represented mean ± SEM. *P*-values determined by Student 2-tailed unpaired t test (I). ***, *P* <0.001. n.s., not significant.

Supplemental Figure 4

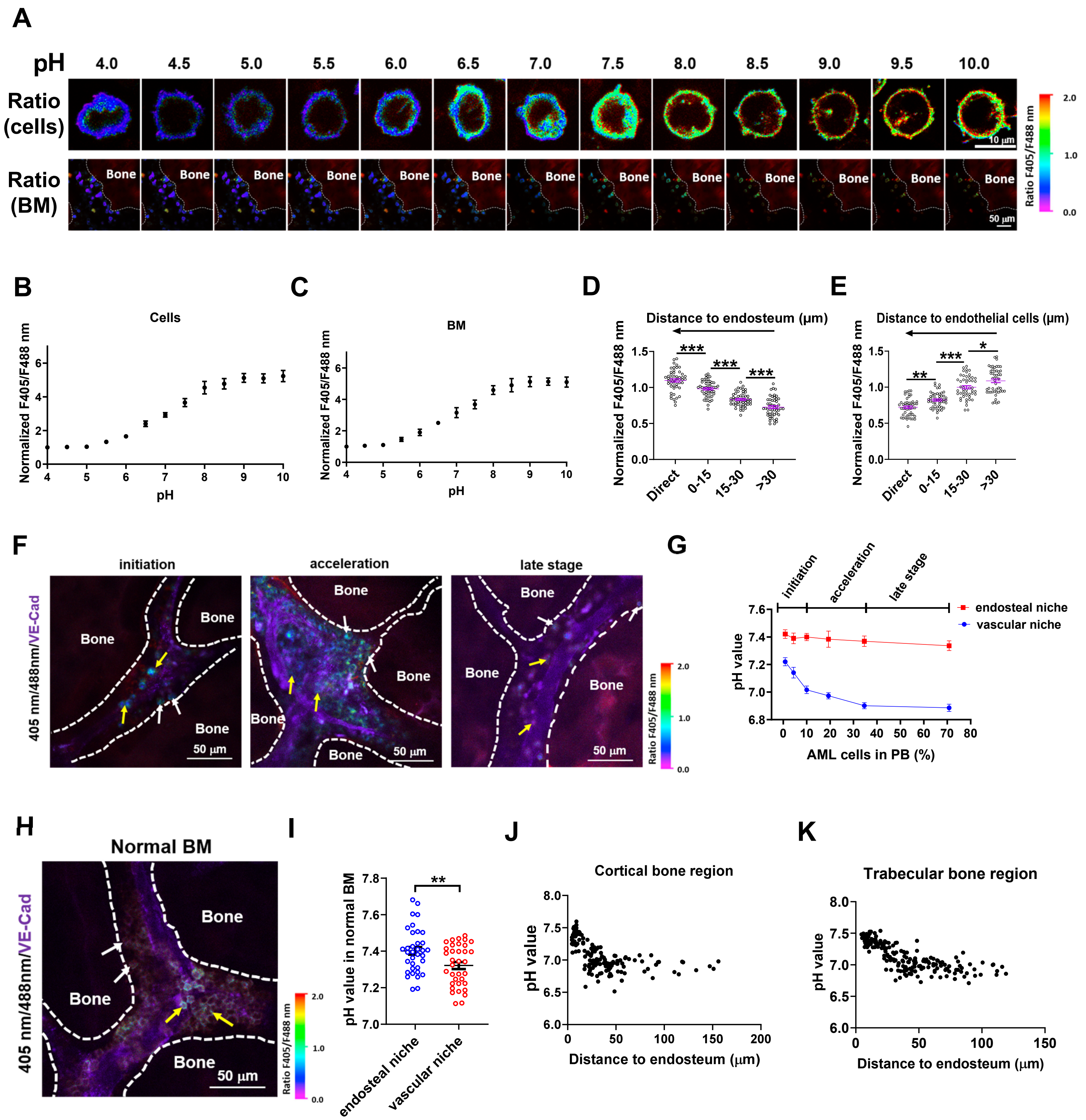

Supplemental Figure 4. (**A**) Representative images of the ratios of fluorescence in cell surface-pHluorin3-expressing THP-1 cells upon in vitro and in vivo treatment with acidic or alkalic buffer between pH 4.0 and 10.0. (**B** and **C**) Correlations between the ratios of cell surface-pHluorin3 fluorescence and pH changes as plotted as S-curve (n = 8-12). (**D** and **E**) Quantification of fluorescence ratios grouped by distance to endosteum (D) or endothelium (E) (n = 50). (**F** and **G**) Representative images of pHluorin3-expressing MLL-AF9$^+$ AML cells in the bone marrow microenvironment at different disease stages (F). MLL-AF9$^+$ mouse AML cells expressing pHluorin3 were injected into recipient mice. Calvaria were imaged at distinct stages of AML progression (initiation, acceleration, and late stage), focusing on areas commonly infiltrated by leukemic cells. Local pH in the endosteal and vascular niches was evaluated by calculating the fluorescence excitation ratio of pHluorin3 (405 nm/488 nm) (G, n = 30-50). White arrows indicate AML cells localized to the endosteal niche, and yellow arrows indicate those in the vascular niche. VE-cadherin$^+$ vasculature is shown in purple and white dashed lines mark the bone surface. Scale bar, 50 µm. (**H** and **I**) Representative image and quantification of pHluorin3-expressing normal bone marrow cells in the BM microenvironment. pHluorin3$^+$ mouse Lin$^-$ cells were transplanted into recipient mice, and calvaria were imaged 4 weeks post-transplantation (H). Local pH in the endosteal and vascular niches was assessed by calculating the fluorescence excitation ratio of pHluorin3 (405 nm/488 nm) (I, n = 35). White arrows indicate cells localized to the endosteal niche, and yellow arrows indicate those in the vascular niche. VE-cadherin$^+$ vasculature is shown in purple and white dashed lines denote the bone surface. Scale bar, 50 µm. (**J** and **K**) Two-photon imaging was performed on pHluorin3-expressing MLL-AF9$^+$ mouse AML cells in both the cortical and trabecular bone regions of the femur. Local pH values were quantified based on their distance from the endosteal surface (n = 160 for J, 190 for K). Data are represented mean ± SEM. *P*-values determined by 1-way ANOVA with Tukey's multiple comparison test (D and E) and Student 2-tailed unpaired t test (I). *, $P < 0.05$; **, $P < 0.01$; ***, $P < 0.001$.

Supplemental Figure 5

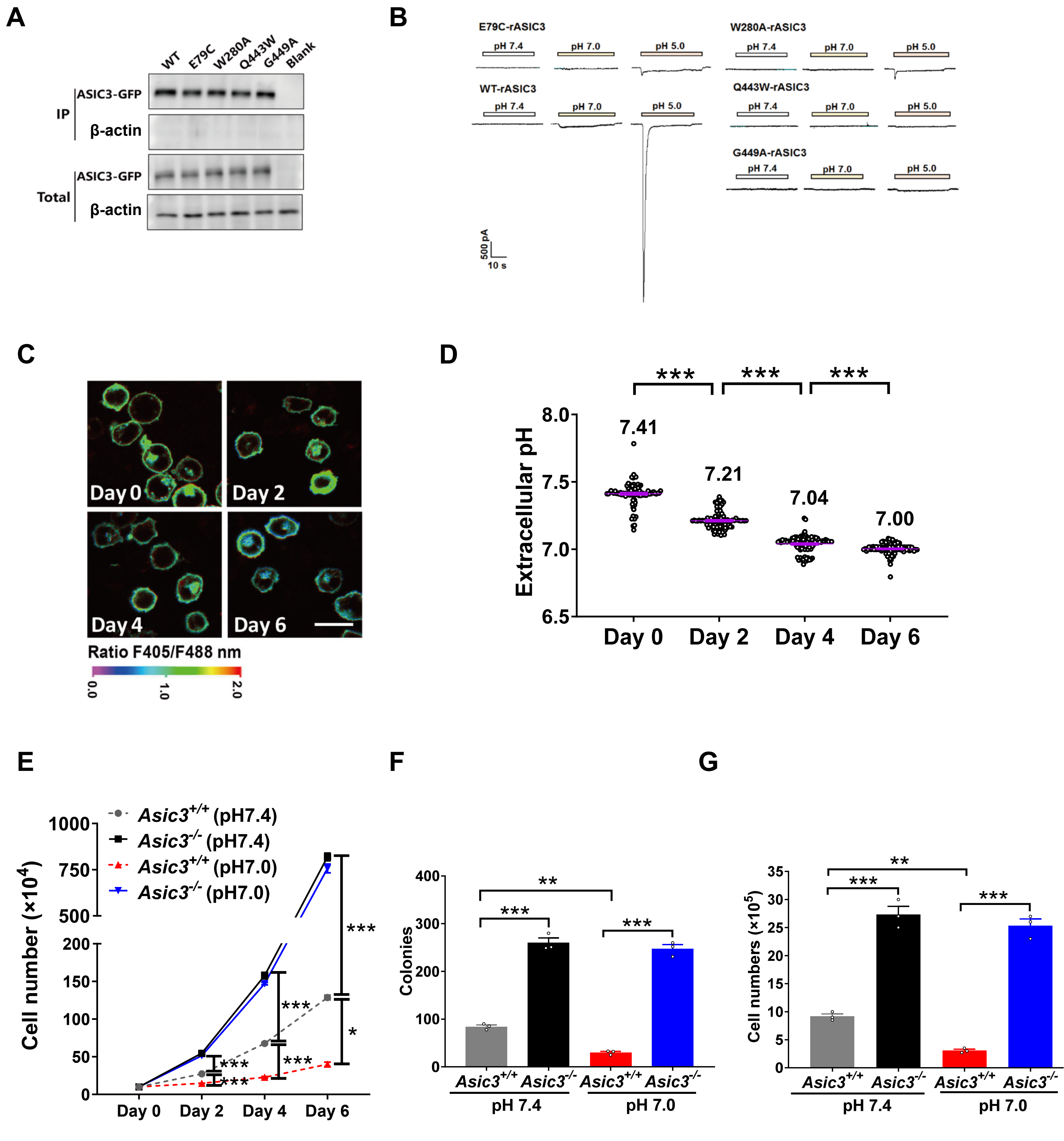

Supplemental Figure 5. (**A**) Immunoprecipitation showing membrane expression of ASIC3 mutants (E79C, W280A, Q443W, G449A) in CHO cells. (**B**) Whole-cell currents triggered by different pH in CHO cells expressing ASIC3 mutants. (**C** and **D**) Representative images of the ratios of m-pHluorin3 fluorescence with excitation at 405 nm and 488 nm (F405/F488 nm) in murine cell surface-pHluorin3-expressing AML cells at indicated time points after culture (C). Scale bar, 10 μm. Quantification of the ratios of cell surface-pHluorin3 fluorescence in panel C is shown (D, n = 100). (**E**-**G**) Murine Mac-1^+c-Kit^+ LICs were cultured in culture medium (pH = 7.0) for 6 days (E, n = 3), followed by the colony formation assay. Colony numbers (F, n = 3) and their derived cell accounts were determined (G, n = 3). Data are represented mean ± SEM. *P*-values determined by 1-way ANOVA with Tukey's multiple comparison test (D-G). *, $P < 0.05$; **, $P < 0.01$; ***, $P < 0.001$.

Supplemental Figure 6

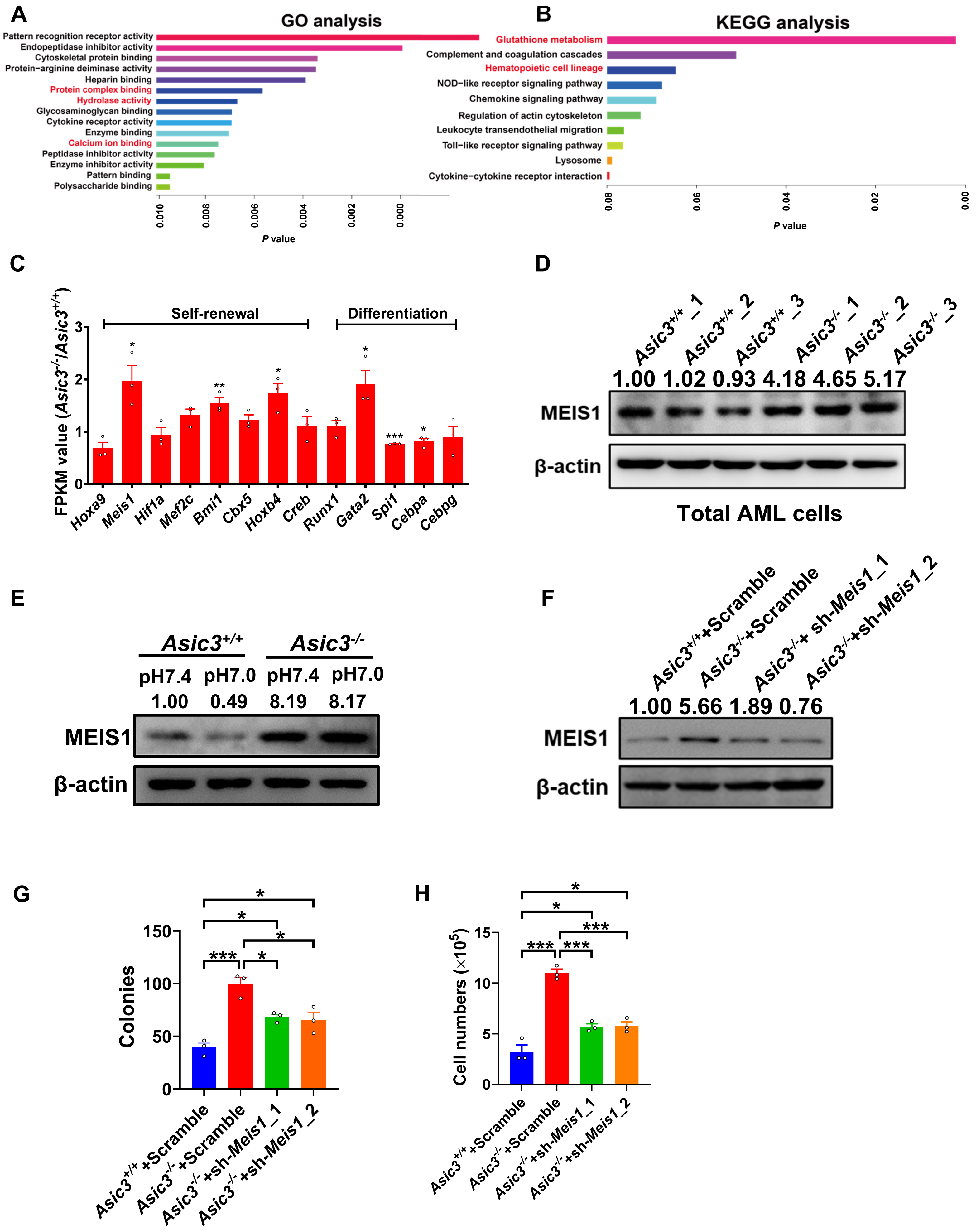

Supplemental Figure 6. (**A** and **B**) GO and KEGG analyses of the RNA-sequencing from WT or *Asic3*-null LICs (n = 3). (**C**) RNA-sequencing analyses were performed with WT and *Asic3*-null YFP$^+$Mac-1$^+$c-Kit$^+$ LICs and potential candidate genes related to self-renewal and differentiation are shown (n = 3). (**D**) Protein levels of MEIS1 in both WT and *Asic3*-null bulk of leukemia cells were evaluated by immunoblotting. Ratios of MEIS1/β-actin were quantified and normalized against *Asic3*$^{+/+}$_1. (**E**) MEIS1 protein levels in WT and *Asic3*-null LICs pretreated with pH 7.0 or pH 7.4 medium; MEIS1/β-actin ratios normalized to WT under pH 7.4. (**F**) Knockdown efficiency of MEIS1 was determined in WT+Scramble, *Asic3*-null+Scramble, and *Meis1*-knockdown *Asic3*-null AML cells by immunoblotting. Ratio of MEIS1/β-actin were quantified and normalized against *Asic3*$^{+/+}$+Scramble. (**G** and **H**) Colony forming abilities were measured in WT, *Asic3*-null, and *Meis1*-knockdown *Asic3*-null AML cells, colony numbers (G, n = 3) and their derived cell accounts were determined (H, n = 3). Data are represented mean ± SEM. *P*-values determined by Student 2-tailed unpaired *t* test (C) and 1-way ANOVA with Tukey's multiple comparison test (G and H). *, $P<0.05$; **, $P<0.01$; ***, $P<0.001$.

Supplemental Figure 7

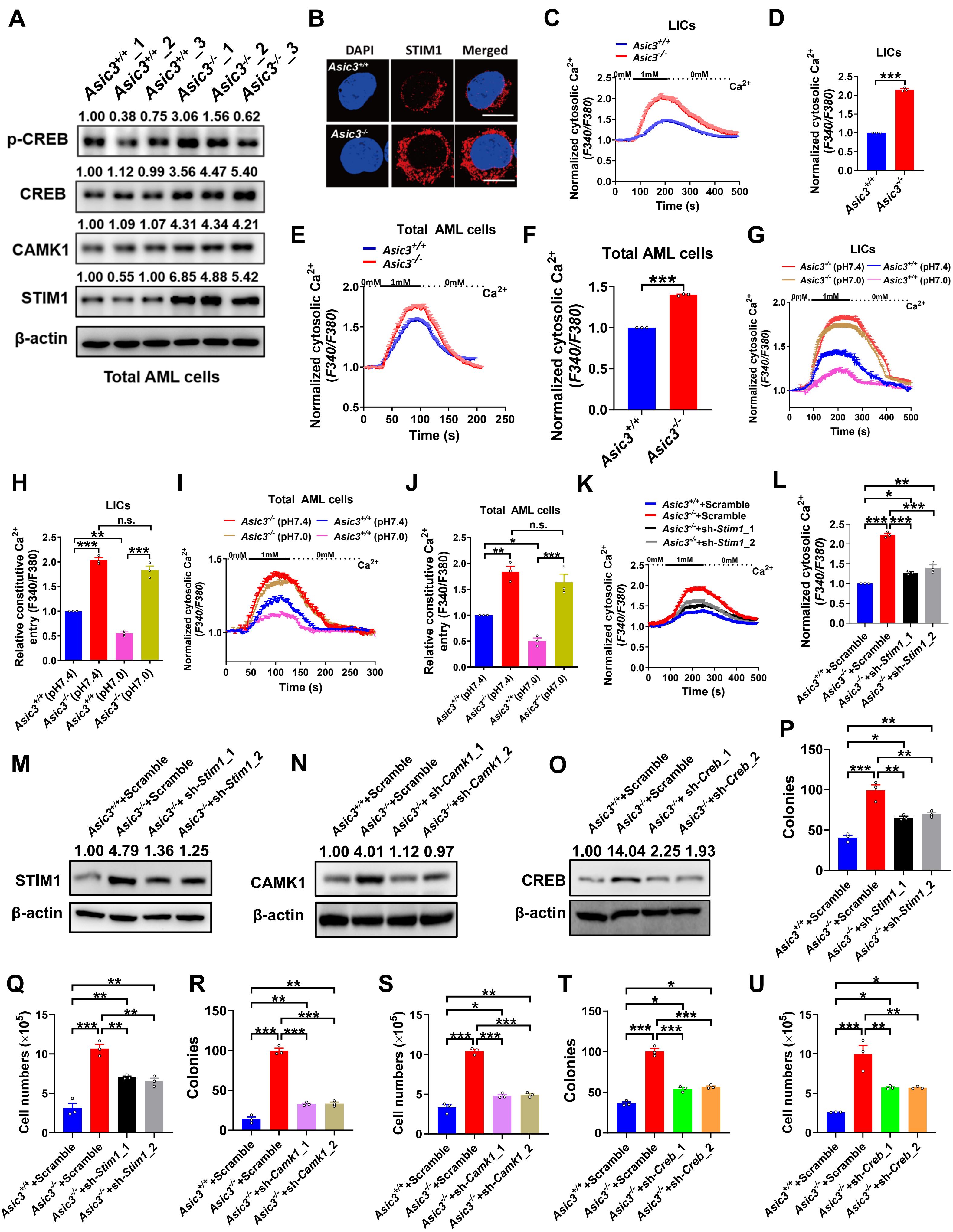

Supplemental Figure 7. (**A**) Protein levels of phospho-CREB, CREB, CAMK1 and STIM1 were determined in WT and *Asic3*-null bulk of leukemia cells using immunoblotting. Ratios of these proteins/β-actin were quantified and normalized against *Asic3*$^{+/+}$_1. (**B**) STIM1 expression/localization in WT and *Asic3*-null LICs by immunofluorescence. Scale bar, 10 μm. (**C** and **D**) Constitutive capacities of calcium influx by Fura-2 AM in WT and *Asic3*-null LICs (C, n = 59 for WT and n = 36 for *Asic3*-null). Quantitative data in panel D. (**E** and **F**) Constitutive capacities of calcium influx (B, n = 35 for WT and n = 38 for *Asic3*-null) were measured in WT and *Asic3*-null bulk of BM leukemia cells. Quantitative data are shown in panel F. (**G** and **H**) Constitutive capacities of calcium influx in WT and *Asic3*-null LICs with treatment of acid buffer, or in WT, *Asic3*-null LICs (G, n = 73-79). Quantitative data in panel H. (**I** and **J**) Constitutive capacities of calcium influx (I, n = 49-73) were measured in WT and *Asic3*-null bulk of BM leukemia cells with treatment of acid buffer. Quantitative data are shown in panel J. (**K** and **L**) Constitutive capacities of calcium influx (K, n = 23-26) were measured in WT, *Asic3*-null and *Stim1*-knockdown *Asic3*-null LICs. Quantitative data are shown in panel L. (**M**-**O**) Knockdown efficiency of STIM1, CAMK1 and CREB were determined in WT+Scramble, *Asic3*-null+Scramble, and *Stim1*, *Camk1* or *Creb*-knockdown *Asic3*-null AML cells using western blotting. Ratio of these proteins/β-actin were quantified and normalized against *Asic3*$^{+/+}$+Scramble. (**P**-**U**) Colony forming abilities were measured in *Stim1*, *Camk1* or *Creb*-knockdown *Asic3*-null AML cells, colony numbers (P, R, and T, n = 3) and their derived cell accounts were determined (R, S, and U, n = 3). Data are represented mean ± SEM. *P*-values determined by Student 2-tailed unpaired *t* test (D and F) and 1-way ANOVA with Tukey's multiple comparison test (H, J, L, and P-U). *, P <0.05; **, P <0.01; ***, P <0.001; n.s., not significant.

Supplemental Figure 8

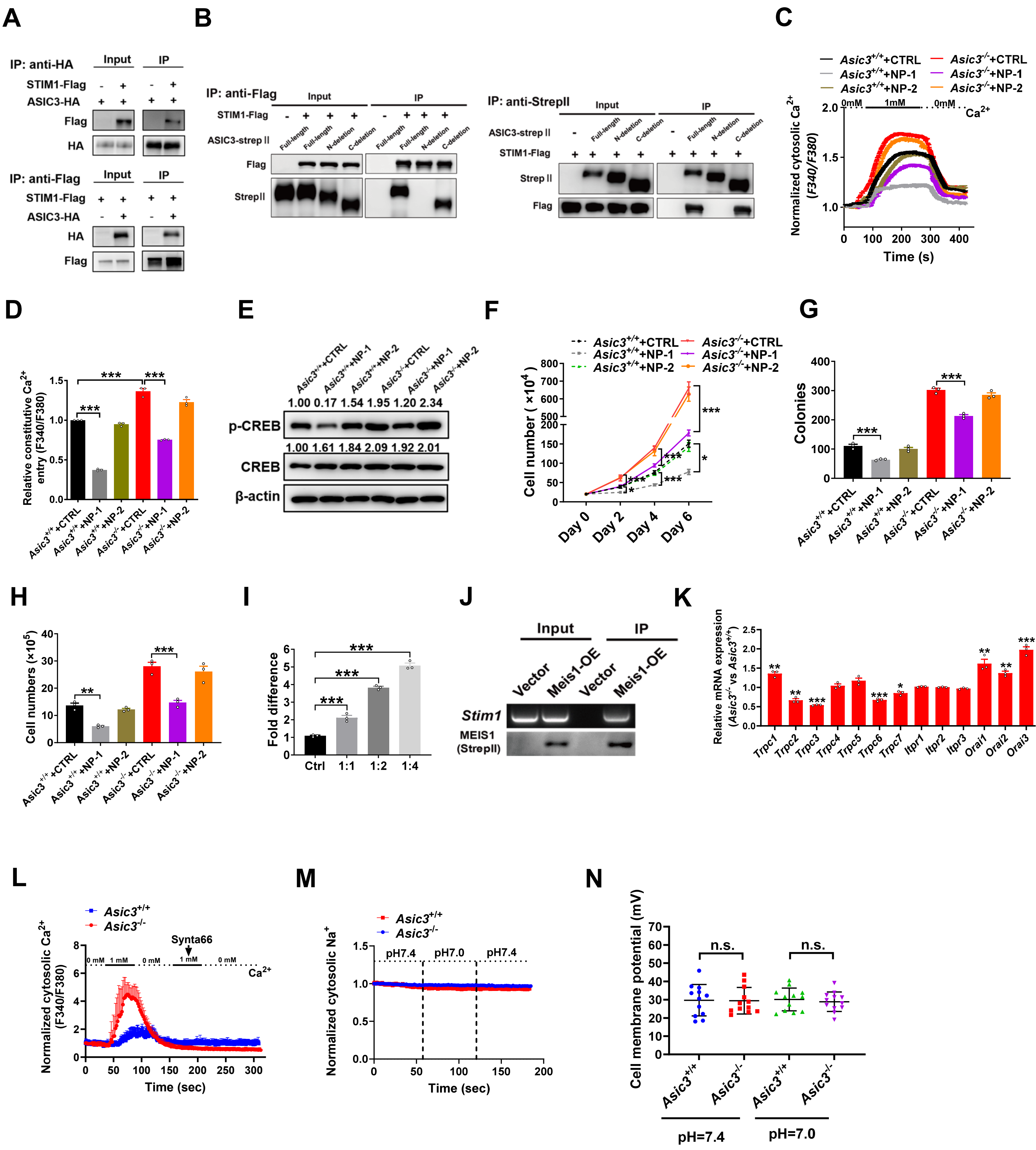

Supplemental Figure 8. (**A**) Co-IP of HA-ASIC3 and Flag-STIM1 in 293T cells using anti-HA or anti-Flag, followed by immunoblot. (**B**) Co-IP of Strep II-tagged ASIC3 (full-length or N-/C-terminal deletions) and Flag-STIM1 in 293T cells using anti-Strep II or anti-Flag, followed by immunoblot. (**C**-**E**) Constitutive capacities of calcium influx (C, n = 120), quantitative data in panel D and p-CREB/CREB levels (E) in WT and *Asic3*-null LICs with/without two ASIC3 N-terminal peptides; protein/β-actin ratios normalized to WT+CTRL. (**F**-**H**) Cell counts, colony numbers, and derived cell numbers in WT and *Asic3*-null LICs with/without peptides (n = 3). (**I**) Luciferase activity of *Stim1* reporter co-transfected with increasing *Meis1* doses in 293T cells (n = 3). (**J**) ChIP of *Meis1* binding to *Stim1* promoter in 293T cells transfected with *Meis1* or empty vector. (**K**) The expression levels of calcium influx-related genes were evaluated in WT and *Asic3*-null LICs as determined by quantitative RT-PCR analysis (n = 3). (**L**) Constitutive capacities of calcium influx were measured in WT and *Asic3*-null LICs with Fura-2 AM and Synta66 (n = 9-10). (**M**) Intracellular Na^+ signals in WT and *Asic3*-null LICs were monitored using CoroNa Green AM (n = 25-30). (**N**) Resting membrane potential measurements were performed on WT and *Asic3*-null LICs using whole-cell patch-clamp electrophysiology at room temperature (n = 12). Data are represented mean ± SEM. *P*-values determined by 1-way ANOVA with Tukey's multiple comparison test (D, F-I) and Student 2-tailed unpaired *t* test (K and N). *, *P* <0.05; **, *P* <0.01; ***, *P* <0.001; n.s., not significant.

Supplemental Figure 9

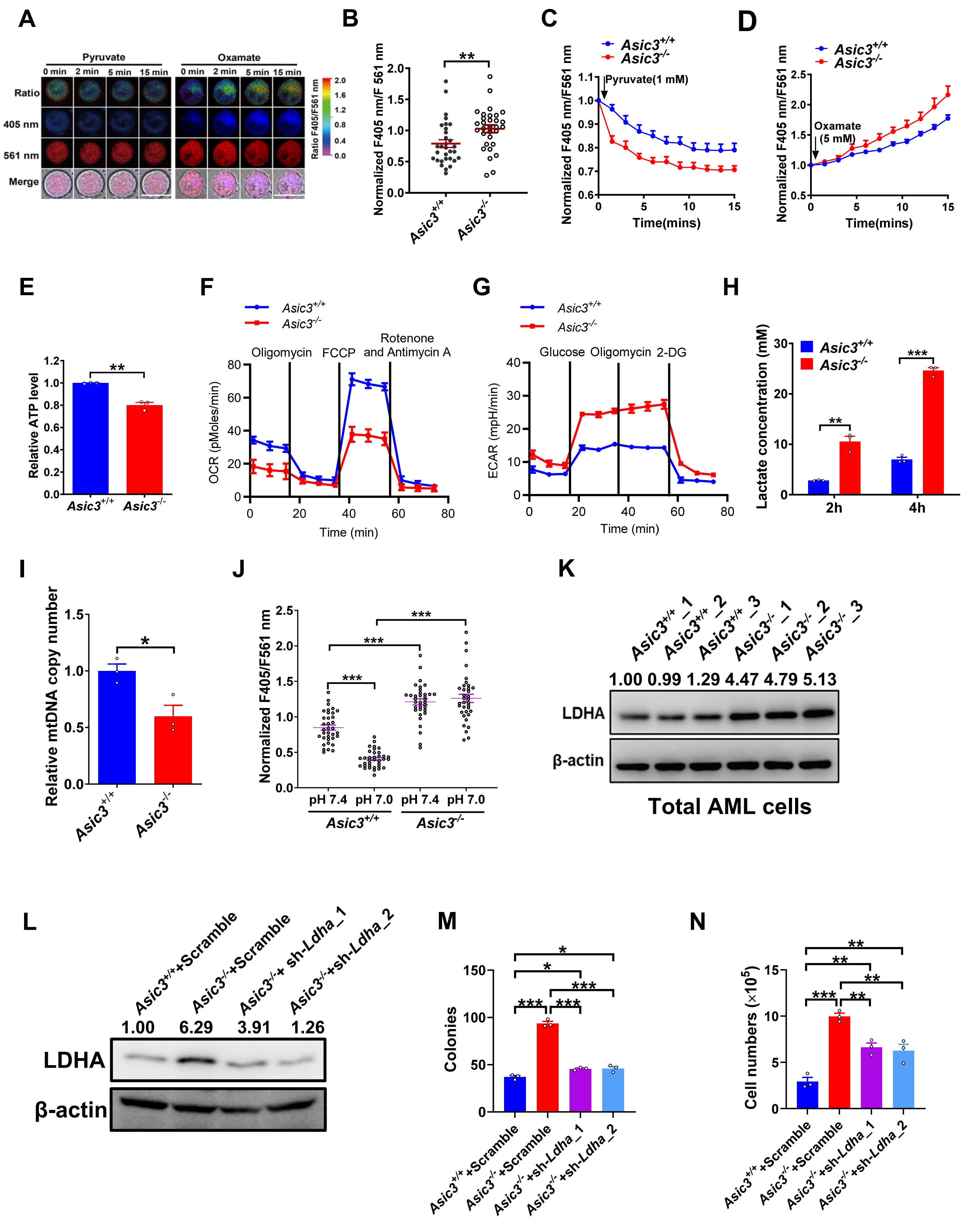

Supplemental Figure 9. (**A**) Representative images of WT SoNar-AML cells showing fluorescence ratios (Ex 405/561 nm). Scale bar, 10 μm. (**B**) Quantified SoNar ratios in WT and *Asic3*-null SoNar-AML cells (n=31). (**C**-**D**) SoNar ratios in WT and *Asic3*-null cells treated with pyruvate (C**,** n = 15-17) or oxamate (D**,** n = 13-15). (**E**) ATP levels in WT and *Asic3*-null LICs (n = 3). (**F**-**G**) Oxygen consumption rate (OCR) and extracellular acidification rate (ECAR) in WT and *Asic3*-null LICs measured by Seahorse XF96 (n = 3). (**H**) Lactate in culture supernatant of WT and *Asic3*-null LICs at indicated times (n = 3). (**I**) Mitochondrial DNA (mtDNA) copies were measured in WT and *Asic3*-null LICs by quantitative RT-PCR (n = 3). (**J**) SoNar ratios in WT and *Asic3*-null cells after 18 h in culture medium (pH 7.0, n = 35). (**K**) Protein levels of LDHA were measured in WT and *Asic3*-null bulk of BM leukemia cells using immunoblotting. Ratios of LDHA/β-actin were quantified and normalized against *Asic3*$^{+/+}$_1. (**L**) Knockdown efficiency of *Ldha* was determined in WT+Scramble, *Asic3*-null+Scramble, and *Ldha*-knockdown *Asic3*-null AML cells using western blotting. Ratio of LDHA/β-actin were quantified and normalized against *Asic3*$^{+/+}$+Scramble. (**M** and **N**) Colony forming abilities were measured in WT, *Asic3*-null, *Asic3*-null and *Ldha*-knockdown *Asic3*-null AML cells, colony numbers (M, n = 3) and their derived cell accounts were determined (N, n = 3). Data are represented mean ± SEM. *P*-values determined by Student 2-tailed unpaired *t* test (B, E, H, I, and J) and 1-way ANOVA with Tukey's multiple comparison test (M and N). *, $P<0.05$; **, $P<0.01$; ***, $P<0.001$.

Supplemental Figure 10

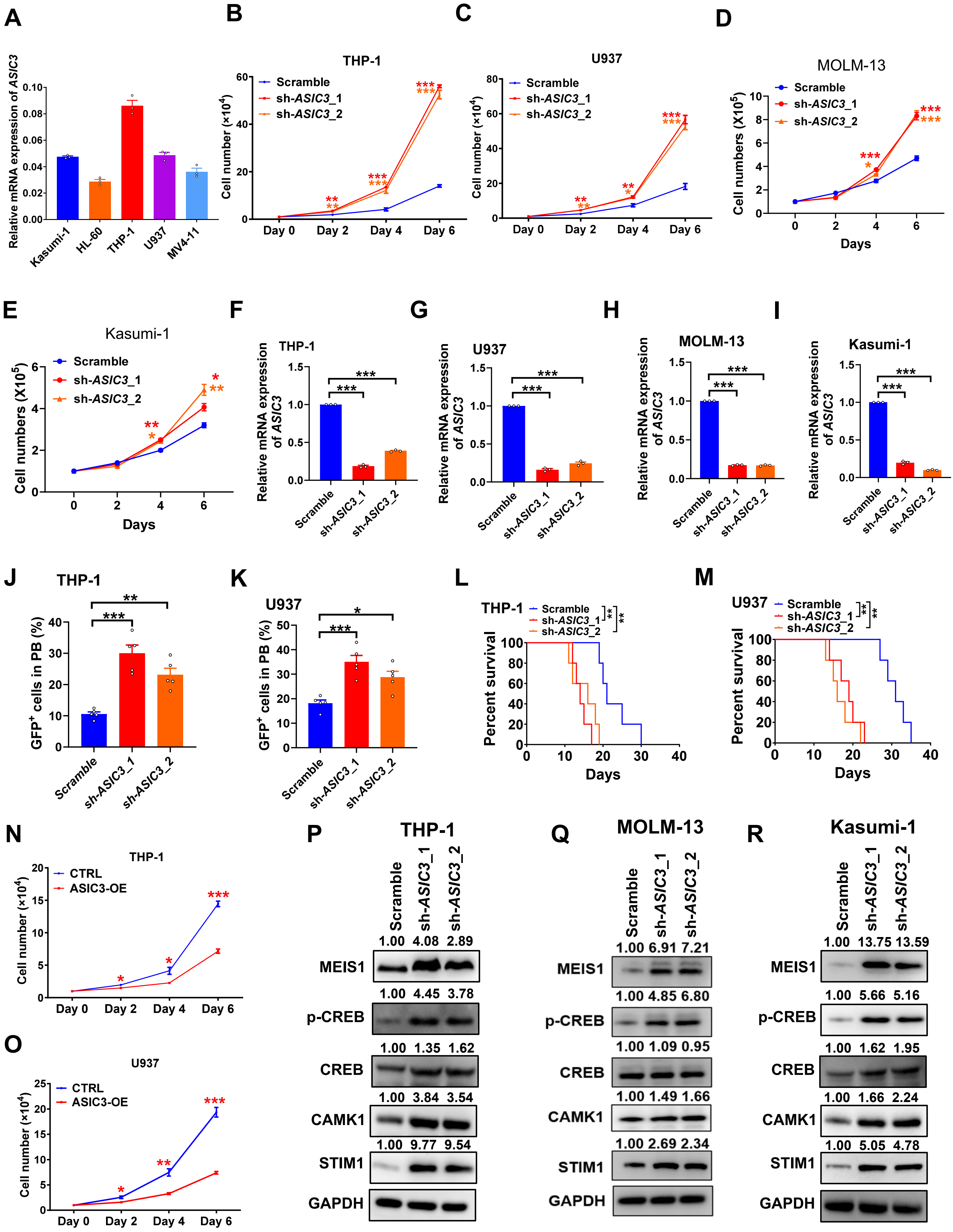

Supplemental Figure 10. (**A**) *ASIC3* mRNA level was determined in different types of human AML cell lines, including Kasumi-1, HL-60, THP-1, U937, and MV4-11 by quantitative RT-PCR (n = 3). (**B**-**E**) THP-1, U937, MOLM-13, or Kasumi-1 cells with sh-*ASIC3*_1-2 or scrambled shRNA; cell counts at indicated times (n = 3). (**F**-**I**) Knockdown efficiency of *ASIC3* was measured in *ASIC3*-knockdown THP-1, U937, MOLM-13, and Kasumi-1 cells by quantitative RT-PCR (n = 3). (**J** and **K**) Flow cytometric analysis for the frequencies of THP-1 or U937 cells in PB 2 weeks after transplantation (n = 5). (**L** and **M**) *ASIC3*-knockdown-THP-1 or U937 cells and control cells were transplanted into NOD-SCID mice. The overall survival was analyzed among the recipients receiving *ASIC3*-knockdown THP-1 or U937 cells and their control ones (n = 5). (**N** and **O**) *ASIC3* was overexpressed (OE) in THP-1 or U937 cells. Cell numbers of THP-1 and U937 cells were calculated at the indicated time points (n = 3). Ctrl: cells were infected with empty vector. (**P**-**R**) Protein levels of MEIS1, phospho-CREB, CREB, CAMK1 and STIM1 were determined in *ASIC3*-knockdown THP-1, MOLM-13, or Kasumi-1 cells by western blotting. Ratio of these proteins/GAPDH were quantified and normalized against Scramble. Data are represented mean ± SEM. *P*-values determined by 1-way ANOVA with Tukey's multiple comparison test (B-K), log-rank test (L and M), and Student 2-tailed unpaired *t* test (N and O). *, $P < 0.05$; **, $P < 0.01$; ***, $P < 0.001$.

Supplemental Figure 11

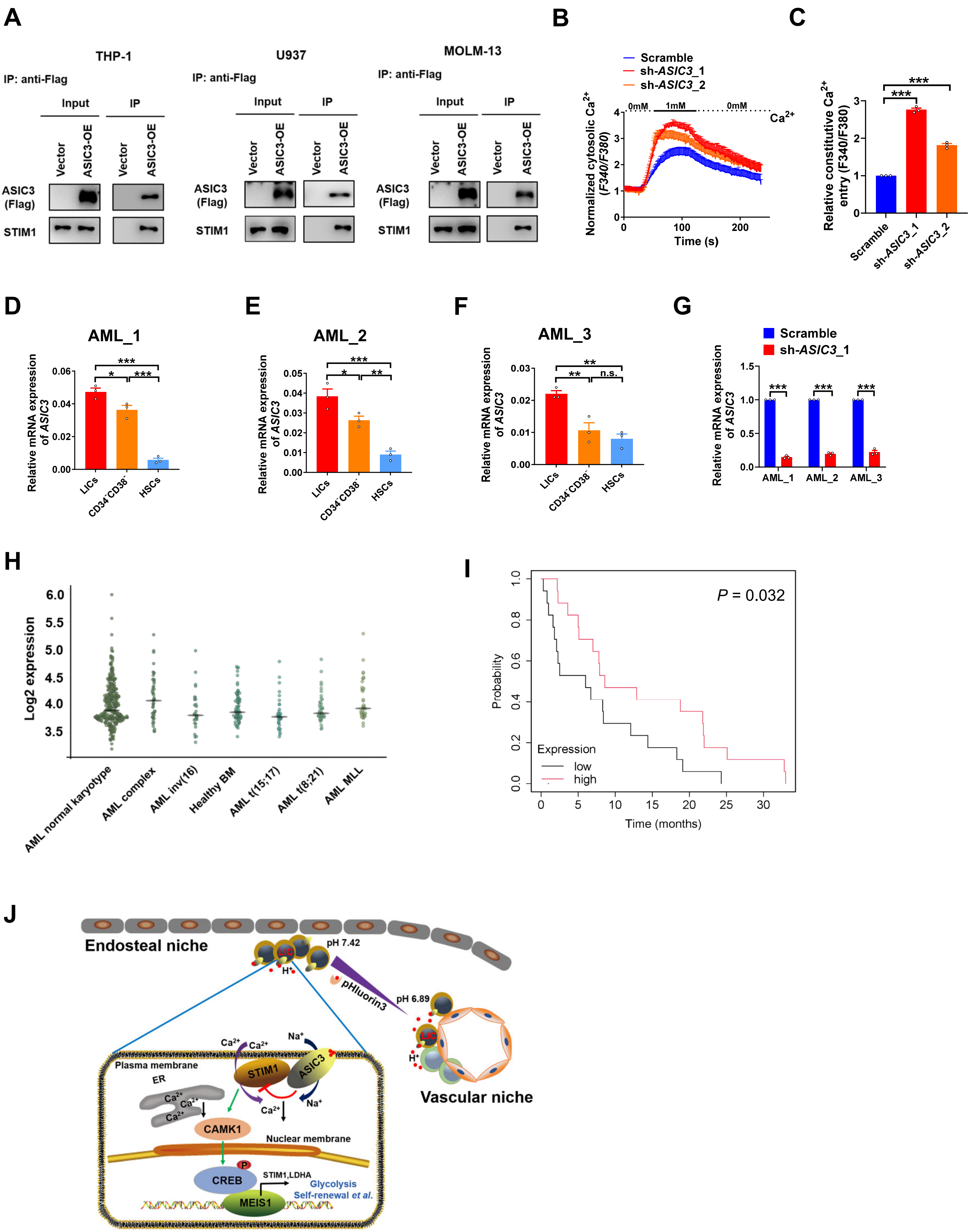

Supplemental Figure 11. (**A**) Flag-tagged full-length ASIC3 and STIM1 were over-expressed in THP-1, U937, or MOLM-13 cells, and their lysates were co-immunoprecipitated using anti-Flag antibody, followed by Western blotting analysis for ASIC3 (Flag) and STIM1. (**B** and **C**) Constitutive capacities of calcium influx (B, n = 10) were evaluated in *ASIC3*-knockdown THP-1 cells and scrambled ones using Fura-2 AM. Quantitative data in panel C. (**D**-**F**) Relative mRNA level of *Asic3* was determined in the immunophenotypic $Lin^-CD34^+CD38^-CD90^-CD45RA^+$ LICs, $CD34^-CD38^-$ differentiated human leukemia cells derived from 3 AML patients or $Lin^-CD34^+CD38^-CD90^+CD45RA^-$ cord blood HSCs (n = 3 technical replicates/group). (**G**) Knockdown efficiency of *ASIC3* was measured in *ASIC3*-knockdown patient-derived AML cells by quantitative RT-PCR (n = 3). (**H**) *ASIC3* mRNA in AML subtypes from BloodSpot databse. (**I**) Correlation of *ASIC3* expression with overall survival in AML patients from the GSE8970 dataset using the KM-plotter database (n = 17 per group, median cut-off). (**J**) Working model for the functions of ASIC3 in leukemogenesis. Data are represented mean ± SEM. *P*-values determined by 1-way ANOVA with Tukey's multiple comparison test (C-F), Student 2-tailed unpaired *t* test (G), and log-rank test (I). *, $P < 0.05$; **, $P < 0.01$; ***, $P < 0.001$; n.s., not significant.

Supplemental Table 1. Limiting dilution assay for WT and *Asic3*-null LICs.

Quantification of CRUs	Survival ratio	
Transplanted cells	*Asic3*$^{+/+}$	*Asic3*$^{-/-}$
20	5/6	1/6
50	2/5	0/5
200	1/5	0/5
Frequency of LICs	1:93	1:11
95% confidence interval	(42-204)	(4-26)

Supplemental Table 2. Effect of different ions, redox agents on pHluorin3

Ions/agents	Concentration	Fold change in excitation ratio (400 nm/485 nm) ± error (SEM)
H^+	pH 5-9	8.611±0.144
Na^+	300 mM	1.096±0.017
K^+	300 mM	1.031±0.013
Cl^-	300 mM	1.036±0.015
Ca^{2+}	10 mM	1.061±0.017
Mg^{2+}	10 mM	1.044±0.029
HCO_3^-	10 mM	1.115±0.041
SO_4^{2-}	10 mM	1.049±0.034
PO_4^{3-}	10 mM	1.056±0.022
NO_3^-	10 mM	1.058±0.028
GSH	10 mM	0.966±0.014
DTT	10 mM	1.077±0.014
H_2O_2	0.5 mM	0.979±0.012
Diamide	0.5 mM	1.000±0.010

GSH, reduced glutathione; DTT, dithiothreitol.

Supplemental Table 3. Properties of pHluorin3

pH	ε393	QY393	Brightness	ε472	QY472	Brightness
5.0	14.3	0.30	4.3	31.6	0.54	16.9
5.5	14.3	0.44	6.2	31.6	0.56	17.6
6.0	18.4	0.45	8.2	26.0	0.56	14.6
6.5	20.9	0.46	9.6	19.4	0.57	11.1
7.0	20.9	0.54	11.2	19.4	0.37	7.2
7.5	26.0	0.51	13.2	8.2	0.62	5.0
8.0	28.6	0.44	12.7	6.6	0.46	3.0
8.5	30.6	0.45	13.6	6.6	0.45	3.0
9.0	30.1	0.48	14.3	6.0	0.48	2.9

Note : Photophysical properties of pHluorin3 in different pH buffer were measured at room temperature. Extinction coefficients (ε, $mM^{-1} \cdot cm^{-1}$) were calculated from absorbance (abs) spectra. The quantum yields (QYs) of pHluorin3 were measured against EGFP at pH 7.5 (QY 0.60)[1]. Brightness is defined as the product of extinction coefficient and quantum yield.

Supplementary References

1. Patterson GH, et al. Use of the green fluorescent protein and its mutants in quantitative fluorescence microscopy. *Biophys J.* 1997; 73(5), 2782-2790.

Supplemental Table 4. Primer Sequences

qRT-PCR primers	Sequences
mouse *Asic1a*-F	CACCTTCCCTGCCGTCACTC
mouse *Asic1a*-R	GCCCTGCTCTGTCGTAGAACTCA
mouse *Asic1b*-F	GGCGAGCCCTTTAATCTCCA
mouse *Asic1b*-R	TGCTGAATGTCCAGCATGATC
mouse *Asic2a*-F	GCGCTCAATTACGAGACAATT
mouse *Asic2a*-R	ATGTTCTCATCATGGCTCCCT
mouse *Asic2b*-F	TGCCTTCATGGACCGTCTG
mouse *Asic2b*-R	CATCTTGCTGAATGTCCAGCA
mouse *Asic3*-F	CCTCAGACATCCGGGTGTTC
mouse *Asic3*-R	GGGAAGGTAAGCTGGTGGC
mouse *Asic4*-F	GCAGGAGGAATACCTACCCAT
mouse *Asic4*-R	CTGAGCTCGCTTTCCGCCCGG
mouse *Asic5*-F	CTGCCATCTCCAACTGACCG
mouse *Asic5*-R	CTTCTATAGATGTTGTCGTTG
mouse *Hoxa9*-F	AAAACACCAGACGCTGGAAC
mouse *Hoxa9*-R	TCTTTTGCTCGGTCCTTGTT
mouse *Meis1*-F	CAGAAAAAGCAGTTGGCACA
mouse *Meis1*-R	TGCTGACCGTCCATTACAAA
mouse *Mef2c*-F	GCCAGTTACCATCCCAGTGT
mouse *Mef2c*-R	ATCAGACCGCCTGTGTTACC
mouse *Bmi1*-F	ATCCCCACTTAATGTGTGTCCT
mouse *Bmi1*-R	CTTGCTGGTCTCCAAGTAACG
mouse *Cbx5*-F	GACAGGCGCATGGTTAAGG
mouse *Cbx5*-R	CCTGGGCTTATTGTTTTCACCC
mouse *Hoxb4*-F	CGTGAGCACGGTAAACCCC
mouse *Hoxb4*-R	GTGTTGGGCAACTTGTGGTC
mouse *Camk4*-F	GAGAACCTCGTCCCGGATTAC
mouse *Camk4*-R	ACACAATGGATGTAGCACCCC
mouse *Creb*-F	AGCAGCTCATGCAACATCATC
mouse *Creb*-R	AGTCCTTACAGGAAGACTGAACT
mouse *Runx1*-F	GATGGCACTCTGGTCACCG
mouse *Runx1*-R	GCCGCTCGGAAAAGGACAA
mouse *Gata2*-F	CGACGAGGTGGATGTCTTCT
mouse *Gata2*-R	GCTGTGCAACAAGTGTGGTC
mouse *Spi1*-F	ATGTTACAGGCGTGCAAAATGG
mouse *Spi1*-R	TGATCGCTATGGCTTTCTCCA
mouse *Cebpa*-F	CAAGAACAGCAACGAGTACCG
mouse *Cebpa*-R	GTCACTGGTCAACTCCAGCAC
mouse *Cebpg*-F	TCGGATCACATTGCTCTGATTTC
mouse *Cebpg*-R	TGTGCCTGAGTATGAATGACACT

mouse *Ldha*-F	GTGCCCAGTTCTGGGTTAAG
mouse *Ldha*-R	CTGGGTCCTGGGAGAACAT
mouse *Pkm2*-F	GTGGCTCGGCTGAATTTCTCT
mouse *Pkm2*-R	CACCGCAACAGGACGGTAG
mouse *Glut1*-F	CAGTTCGGCTATAACACTGGTG
mouse *Glut1*-R	GCCCCCGACAGAGAAGATG
mouse *Glut3*-F	ATGGGGACAACGAAGGTGAC
mouse *Glut3*-R	GTCTCAGGTGCATTGATGACTC
mouse *Pdk2*-F	TGGACCGCTTCTACCTCAG
mouse *Pdk2*-R	TCTTTCACCACATCAGACACG
mouse *Pdk4*-F	AGGGAGGTCGAGCTGTTCTC
mouse *Pdk4*-R	GGAGTGTTCACTAAGCGGTCA
mouse *Hk1*-F	CGGAATGGGGAGCCTTTGG
mouse *Hk1*-R	GCCTTCCTTATCCGTTTCAATGG
mouse *Fh1*-F	TACGGAACGTATGCCAATCCC
mouse *Fh1*-R	GGCCTTCATTATTGCACTAGCA
mouse *Idh1*-F	ATGCAAGGAGATGAAATGACACG
mouse *Idh1*-R	GCATCACGATTCTCTATGCCTAA
mouse *Idh2*-F	GGAGAAGCCGGTAGTGGAGAT
mouse *Idh2*-R	GGTCTGGTCACGGTTTGGAA
mouse *Aco2*-F	ATCGAGCGGGGAAAGACATAC
mouse *Aco2*-R	TGATGGTACAGCCACCTTAGG
mouse *Cpt1*-F	CTCCGCCTGAGCCATGAAG
mouse *Cpt1*-R	CACCAGTGATGATGCCATTCT
mouse *Acox1*-F	TAACTTCCTCACTCGAAGCCA
mouse *Acox1*-R	AGTTCCATGACCCATCTCTGTC
mouse *Actb*-F	GGCTGTATTCCCCTCCATCG
mouse *Actb*-R	CCAGTTGGTAACAATGCCATGT
mouse mt-*ND4*-F	GGAACCAAACTGAACGCCTA
mouse mt-*ND4*-R	ATGAGGGCAATTAGCAGTGG
mouse *B2m*-F	TCATTAGGGAGGAGCCAATG
mouse *B2m*-R	ATCCCCTTTCGTTTTTGCTT
mouse *Oria1*-F	GATCGGCCAGAGTTACTCCG
mouse *Oria1*-R	TGGGTAGTCATGGTCTGTGTC
mouse *Oria2*-F	GGCCACAAGGGCATGGATTA
mouse *Oria2*-R	TGAGGGTACTGGTACTTGGTC
mouse *Oria3*-F	GGCTACCTGGACCTTATGGG
mouse *Oria3*-R	GCAGGCACTAAATGCCACC
mouse *Trpc1*-F	TACGGTTGTCAGTCCGCAGA
mouse *Trpc1*-R	TCGTTTTGGCCGATGATTAAGTA
mouse *Trpc2*-F	CTCTCTGGCTGACCCTGTAGT
mouse *Trpc2*-R	GTTGTTTGGGCTTACCACACT
mouse *Trpc3*-F	TCGAGAGGCCACACGACTA
mouse *Trpc3*-R	CTGGACAGCGACAAGTATGC

mouse *Trpc4*-F	GTGTGCTACCTGATAGCTCCC
mouse *Trpc4*-R	GGCAGAGACACGTTCGTTATT
mouse *Trpc5*-F	GGGCTGAGACTGAGCTGTC
mouse *Trpc5*-R	TTGCGGATGGCGTAGAGTAAT
mouse *Trpc6*-F	AGCCAGGACTATTTGCTGATGG
mouse *Trpc6*-R	AACCTTCTTCCCTTCTCACGA
mouse *Trpc7*-F	CTTCCTGGACTCGGCTGAGTA
mouse *Trpc7*-R	GCGTTCTGCCCCATGTAGT
mouse *Itpr1*-F	TAGGATCTCCTGCCTCCTGT
mouse *Itpr1*-R	AGAGCACCTGGCACATTACT
mouse *Itpr2*-F	CAGATCGACAGCAACAAGGG
mouse *Itpr2*-R	GCTGGTGTTGATTACGGCAT
mouse *Itpr3*-F	GACTACCTCTCCGACCTGTG
mouse *Itpr3*-R	GTACTCGTGAGACTGAGCCA
human *ASIC3*-F	GCCTGAGAACTTCACCACGA
human *ASIC3*-R	ATGCCACCCCTAGTAGTGGT

**shRNA for mouse *Meis1***	**Target sequences**
sh-*Meis1*_1	TAGAGAAGGTACACGAATTAT
sh-*Meis1*_2	CCTCGGTCAATGACGCTTTAA

**shRNA for mouse *Creb***	**Target sequences**
sh-*Creb*_1	GCCTGAAAGCAACTACAGAAT
sh-*Creb*_2	CAGCAGCTCATGCAACATCAT

**shRNA for mouse *Camk1* target sequences**	
sh-*Camk1*_1	GAGAACGAGATTGCCGTCTTA
sh-*Camk1*_2	CCAAAGATTTCATACGACATT

**shRNA for mouse *Stim1* target sequences**	
sh-*Stim1*_1	CCCTTCCTTTCTTTGCAATAT
sh-*Stim1*_2	TGGTGGTGTCTATCGTTATTG

**shRNA for mouse *Ldha* target sequences**	
sh-*Ldha* _1	CGTGAACATCTTCAAGTTCAT
sh-*Ldha* _2	CCAGCAAAGACTACTGTGTAA

**shRNA for human *ASIC3* target sequences**

sh-*ASIC3* _1	TTGACATGGCGCAACTCTATG
sh-*ASIC3* _2	GACAAGGTCCTGGGATATTTC
**ChIP for *Stim1***	**Sequences**
ChIP-*Stim1*-F	GTCGAAAGGGCGGAGCCTGGG
ChIP-*Stim1*-R	TGGGCGGGGGCGTGCTCTACC
