# Supplementary material for: Nonionotropic action of an acid-sensing ion channel inhibits leukemogenesis in the acidic bone marrow niche
Source: J Clin Invest. 2025 Dec 15;135(24):e189051. doi: 10.1172/JCI189051 (PMC12700551; doi:10.1172/JCI189051)

Full unedited blot images for Figure 4

used in the figures

Full unedited blot images for Figure 4B

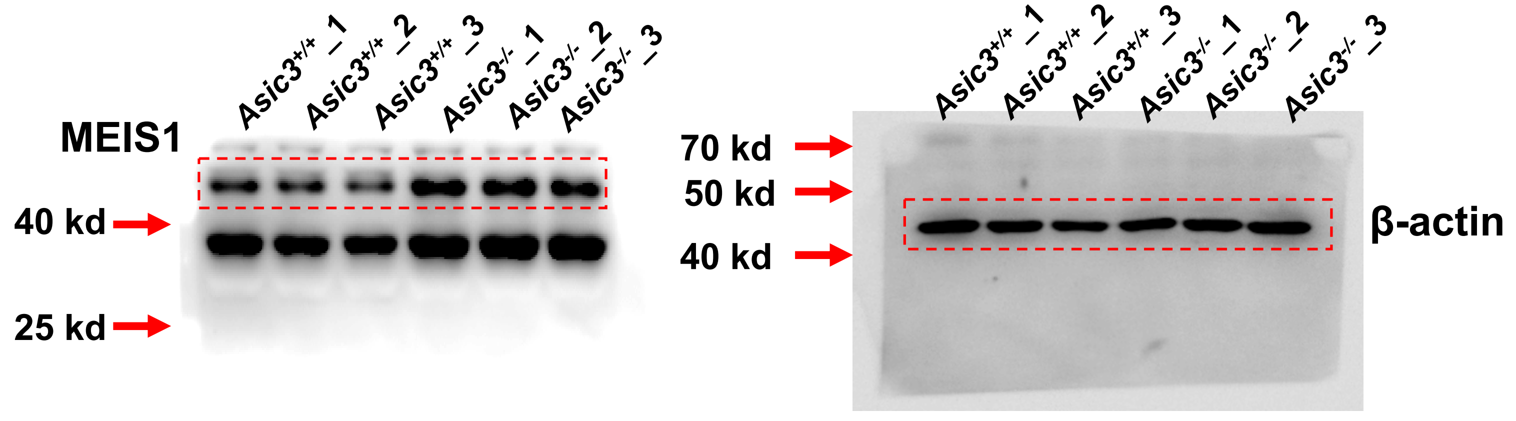

Full unedited blot images for Figure 5

used in the figures

Full unedited blot images for Figure 5A

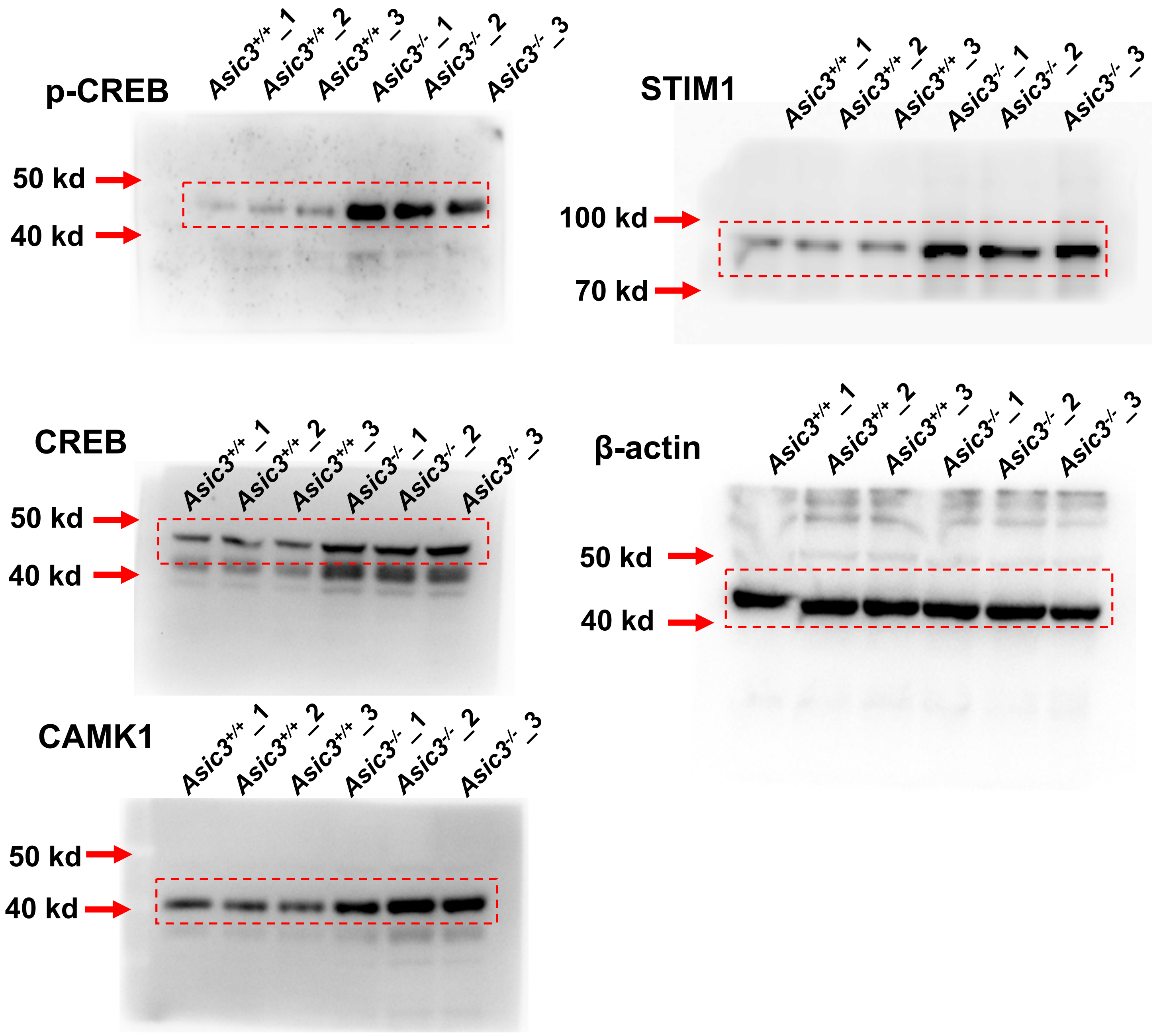

Full unedited blot images for Figure 6

used in the figures

Full unedited blot images for Figure 6B

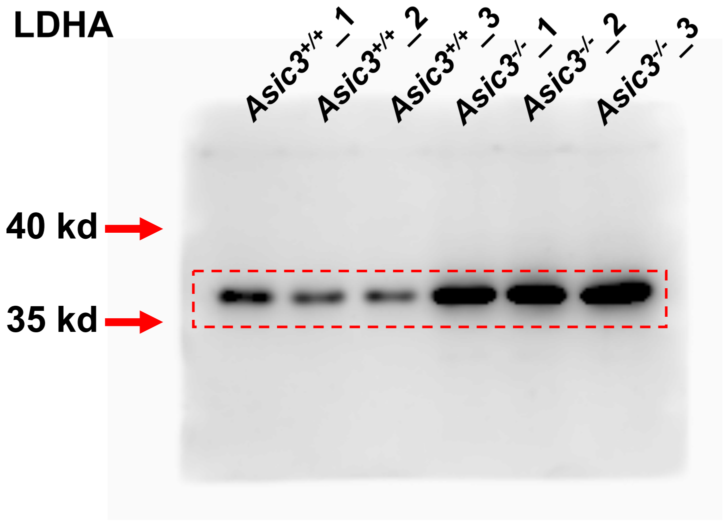

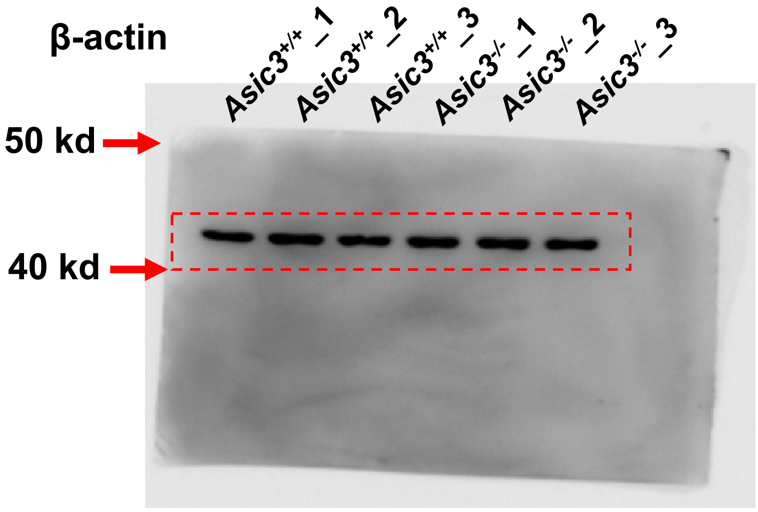

Full unedited blot images for Figure 7

used in the figures

Full unedited blot images for Figure 7G

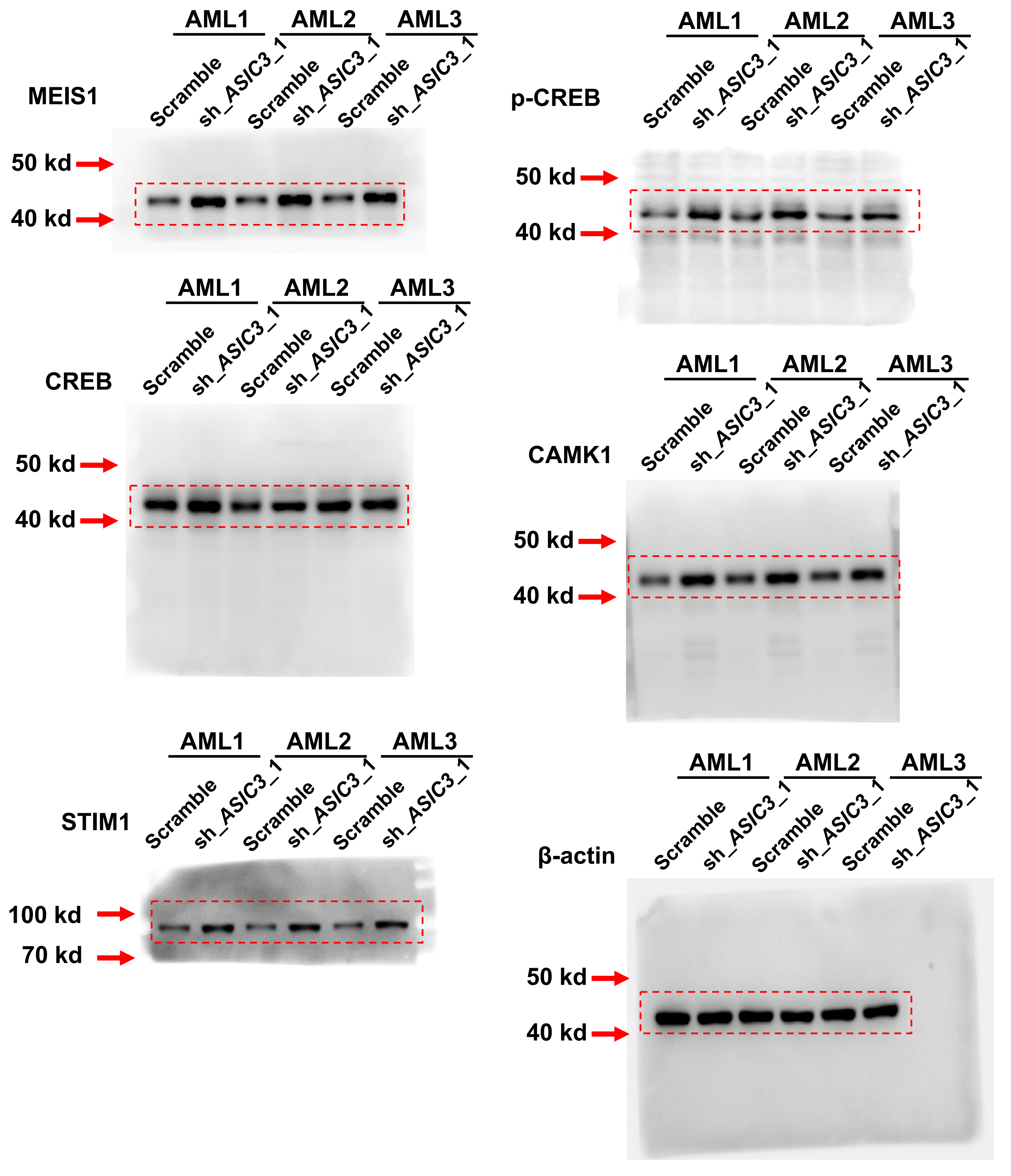

Full unedited gel image for Supplemental Figure 1

used in the figures

Full unedited gel image for Supplemental Figure 1A

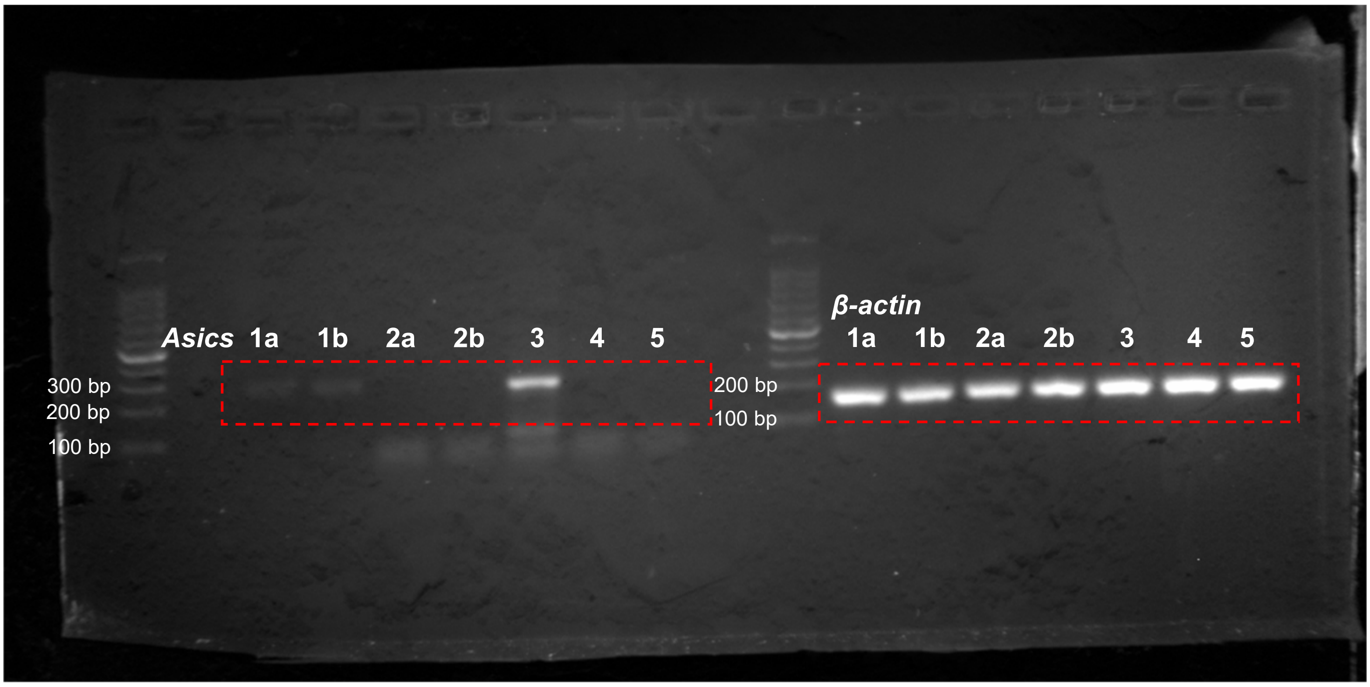

Full unedited gel image for Supplemental Figure 1

used in the figures

Full unedited gel image for Supplemental Figure 1B

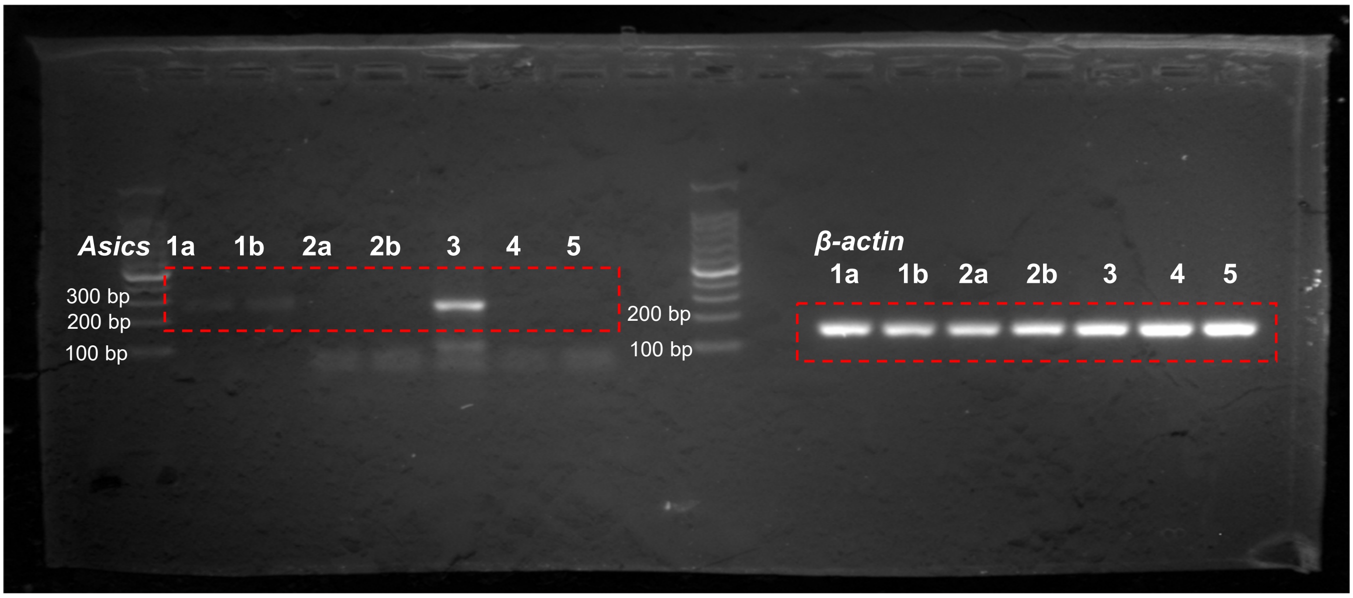

Full unedited blot images for Supplemental Figure 5

used in the figures

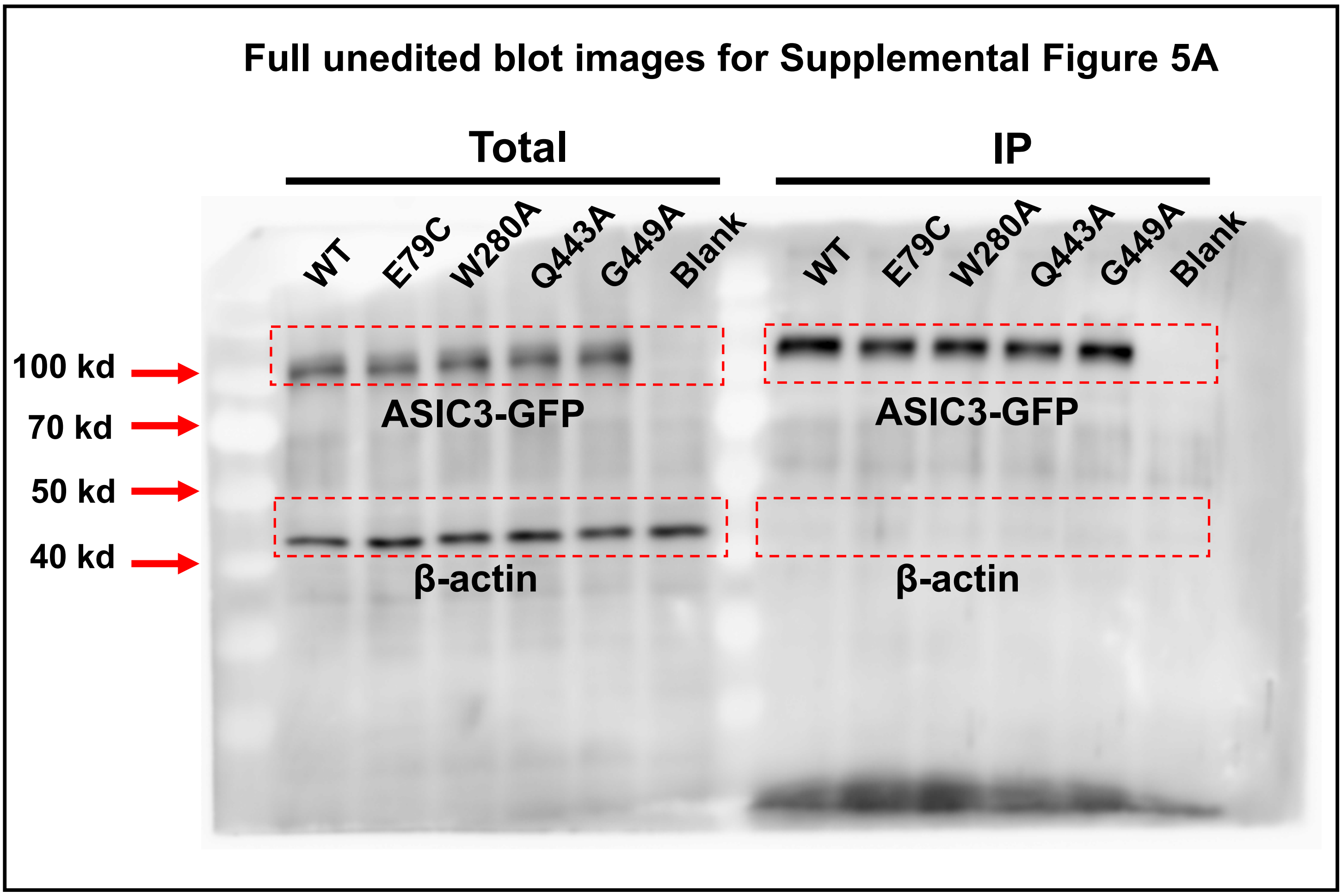

Full unedited blot images for Supplemental Figure 6

used in the figures

Full unedited blot images for Supplemental Figure 6D

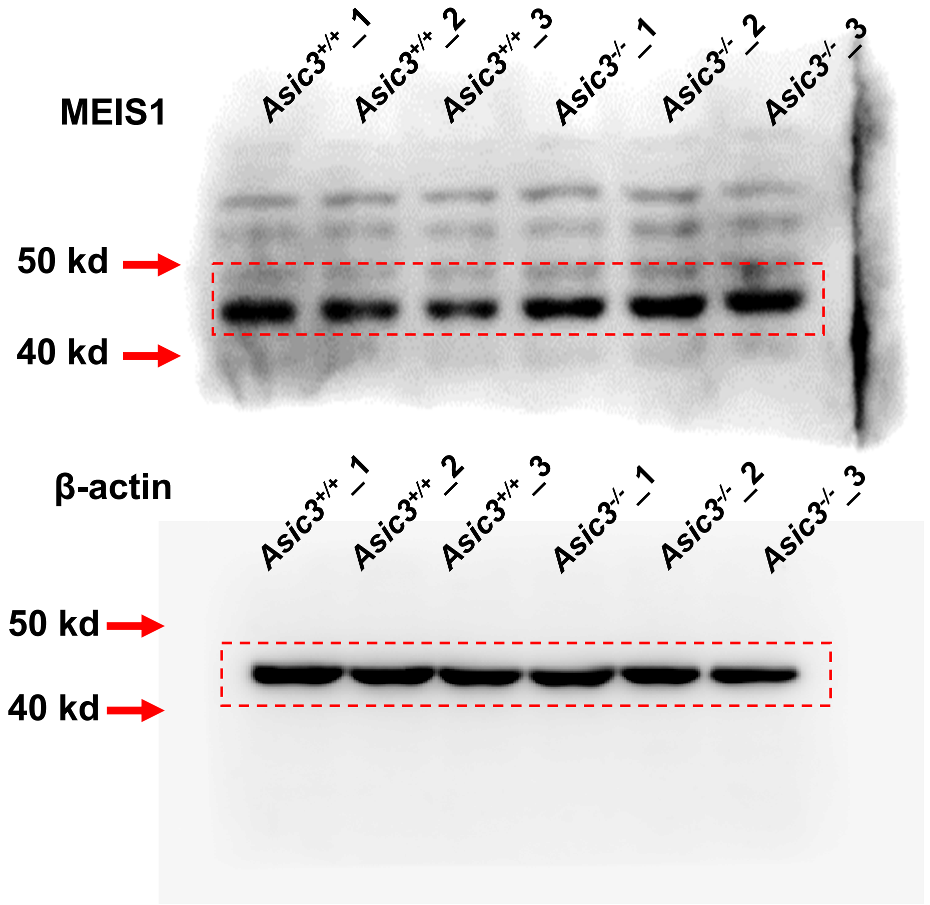

Full unedited blot images for Supplemental Figure 6E

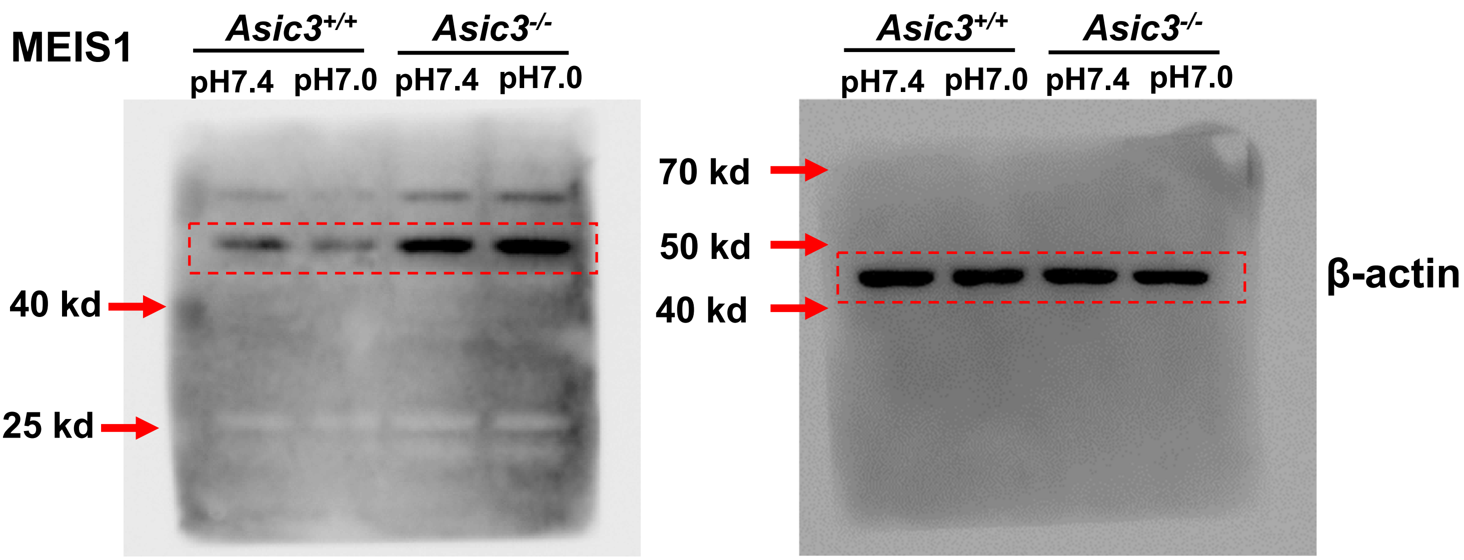

Full unedited blot images for Supplemental Figure 6

used in the figures

Full unedited blot images for Supplemental Figure 6F

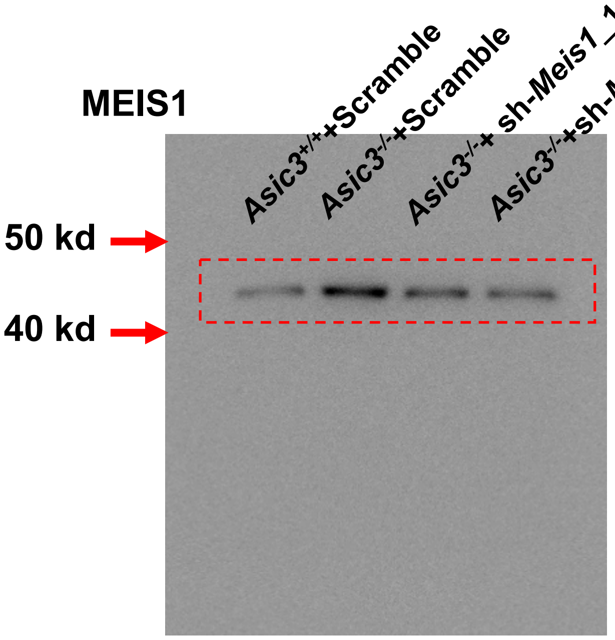

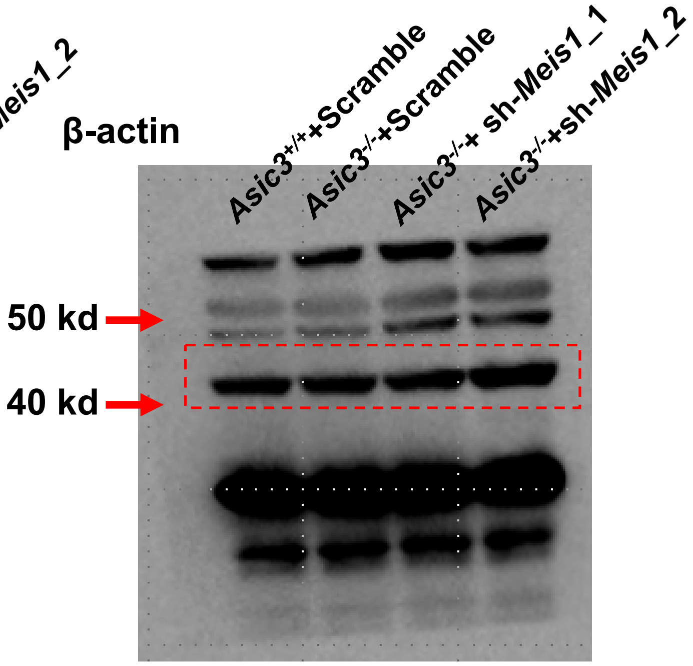

Full unedited blot images for Supplemental Figure 7

used in the figures

Full unedited blot images for Supplemental Figure 7A

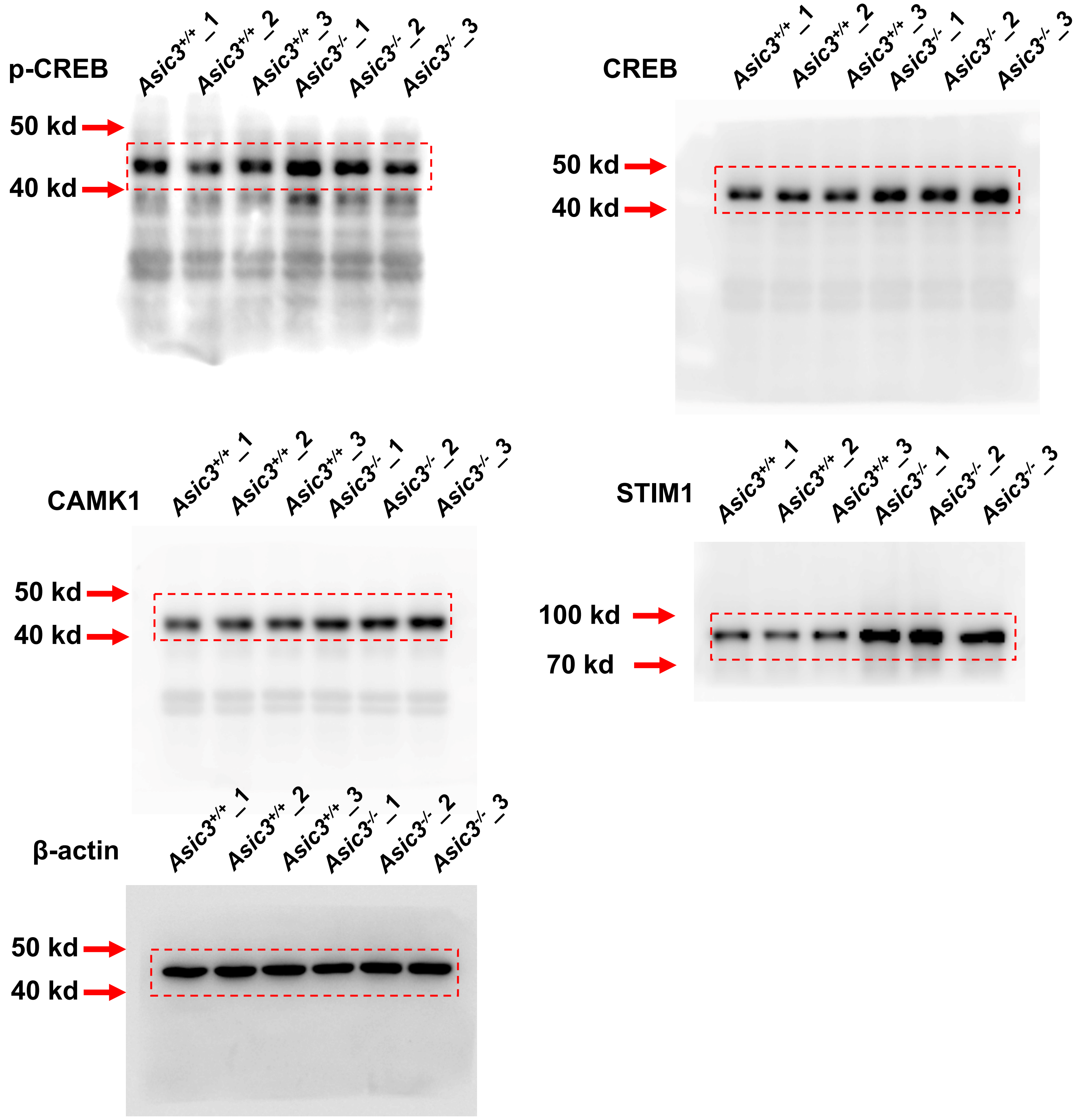

Full unedited blot images for Supplemental Figure 7

used in the figures

Full unedited blot images for Supplemental Figure 7M

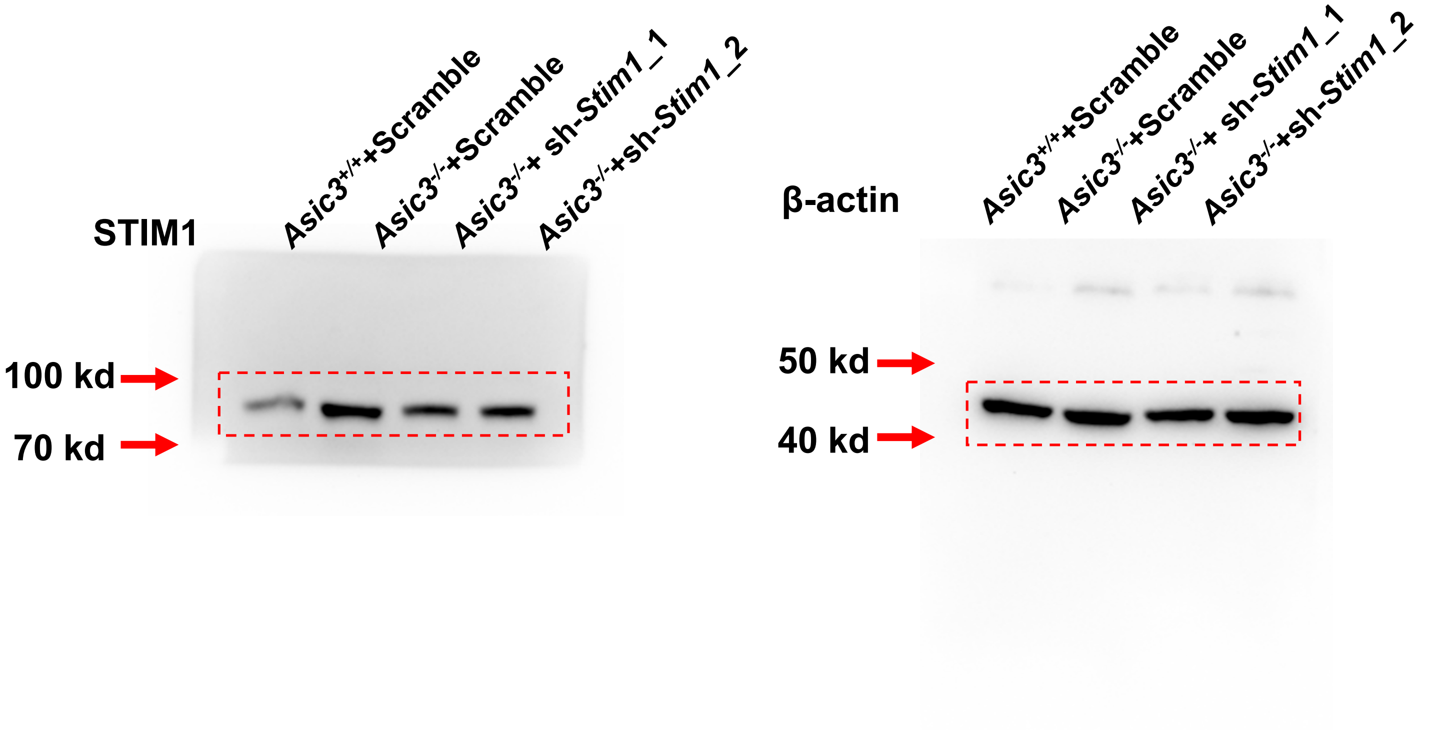

Full unedited blot images for Supplemental Figure 7N

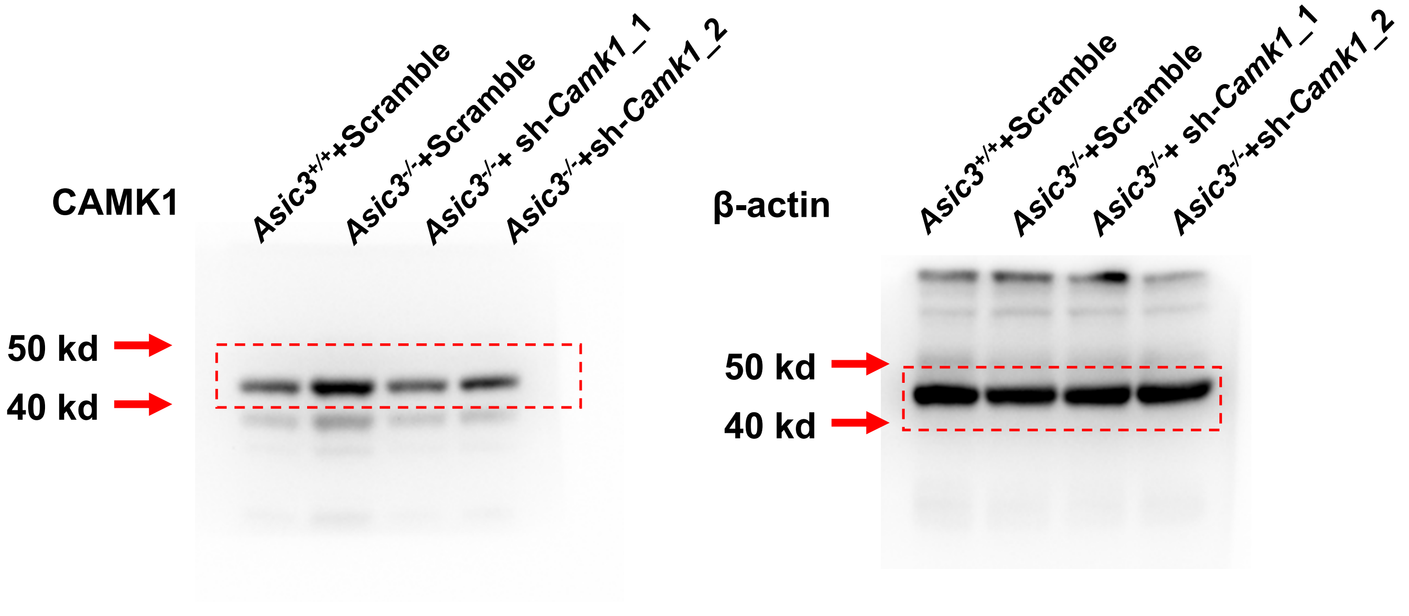

Full unedited blot images for Supplemental Figure 7

used in the figures

Full unedited blot images for Supplemental Figure 7O

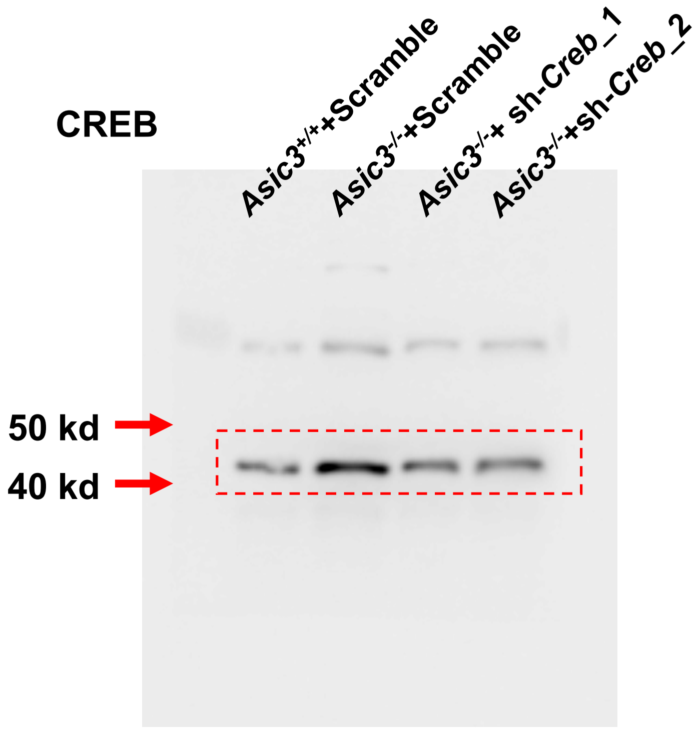

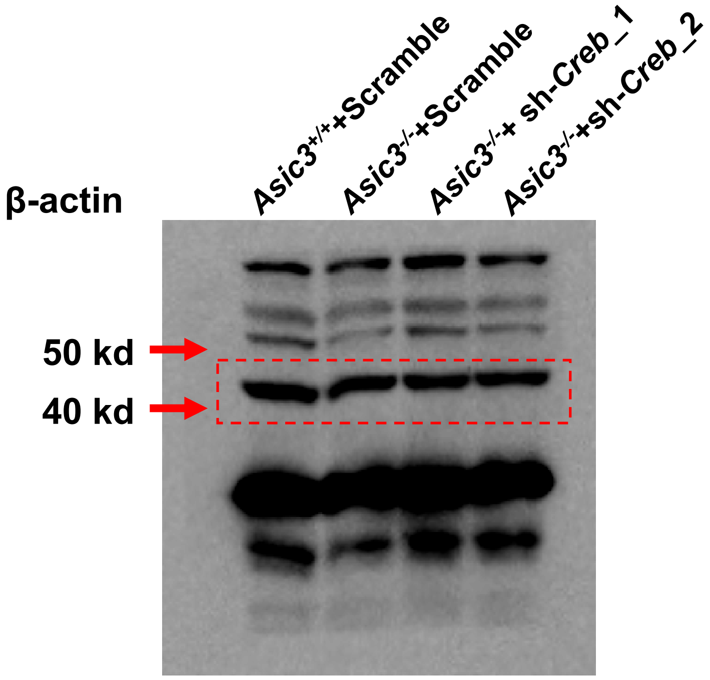

Full unedited blot images for Supplemental Figure 8

used in the figures

Full unedited blot images for Supplemental Figure 8A

IP: anti-HA

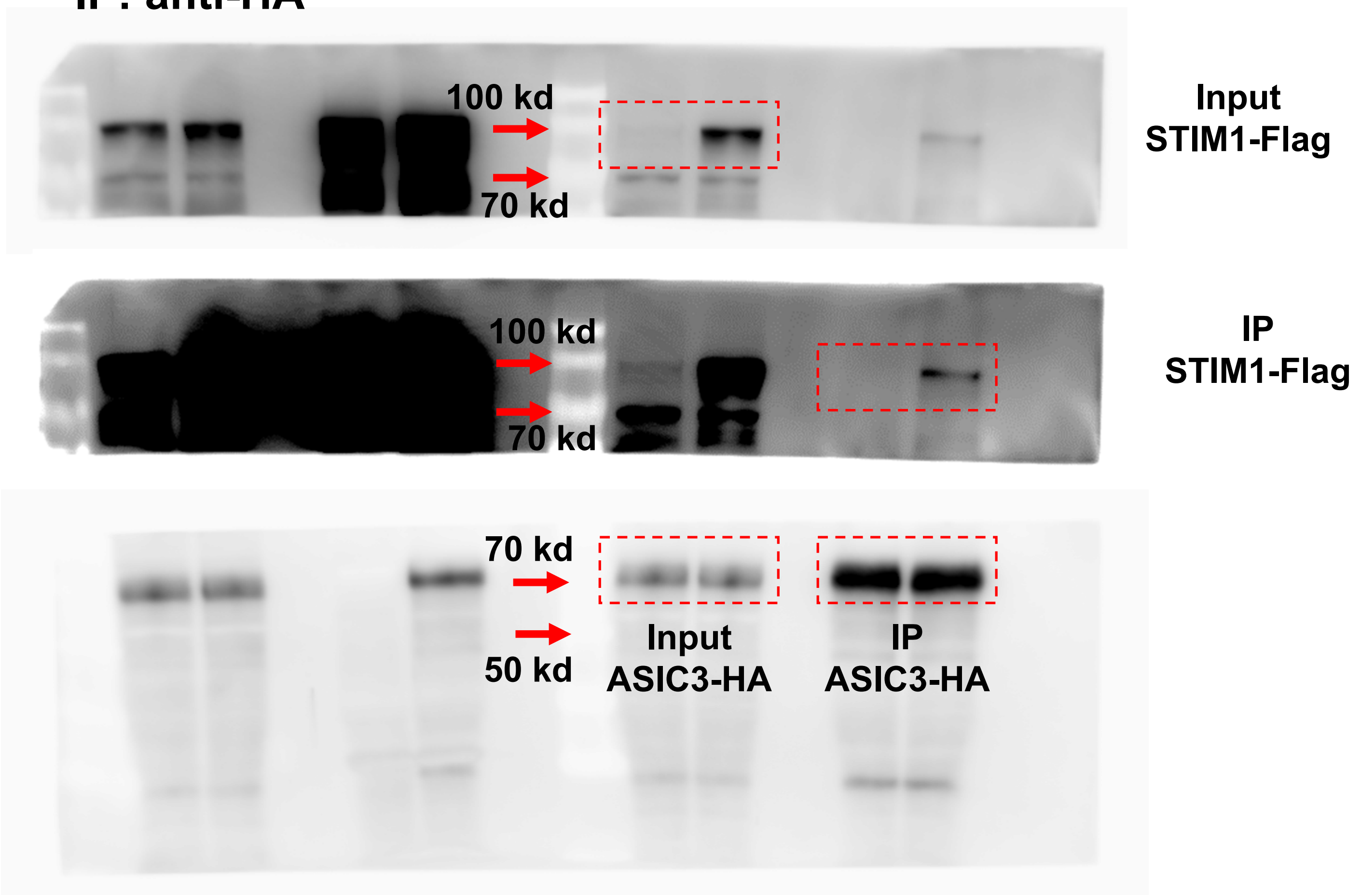

IP: anti-Flag

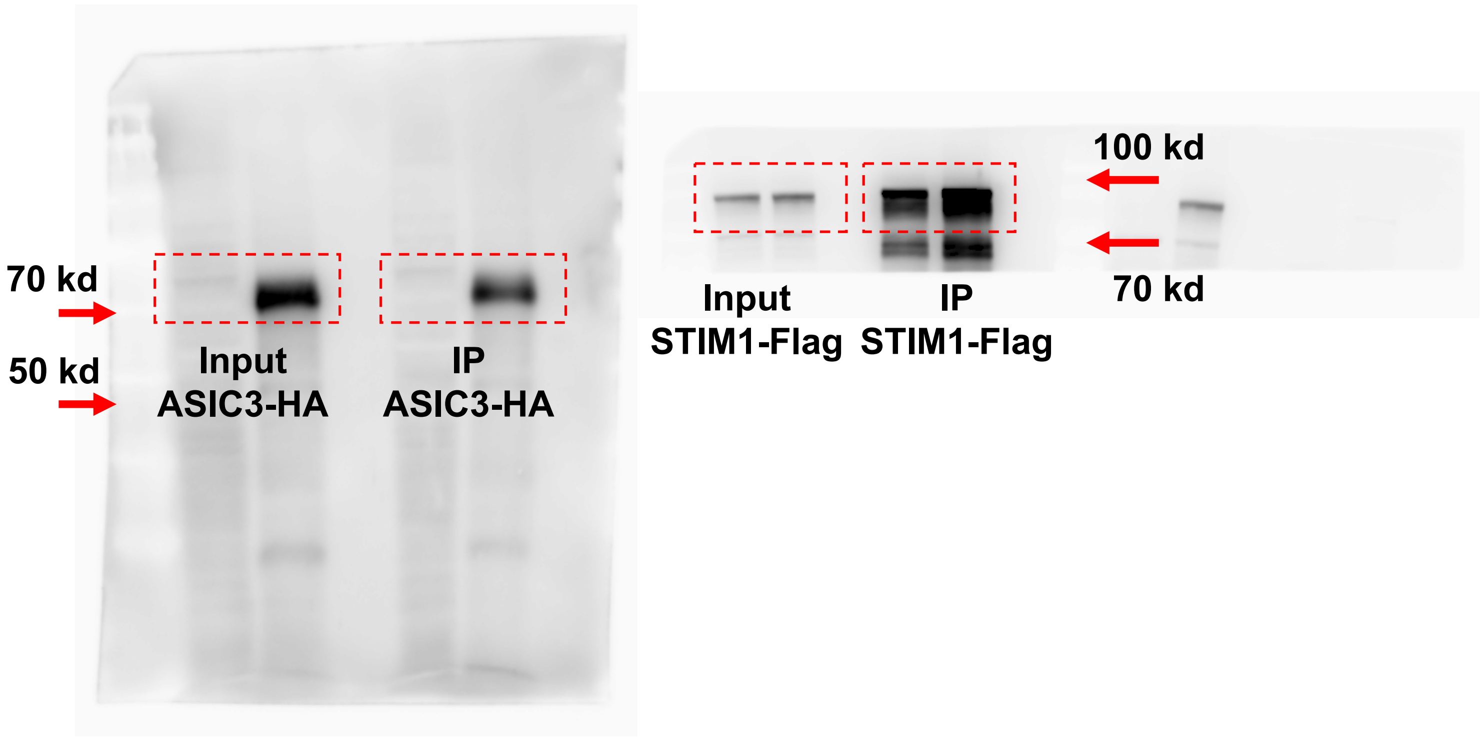

Full unedited blot images for Supplemental Figure 8

used in the figures

Full unedited blot images for Supplemental Figure 8B

IP: anti-Flag

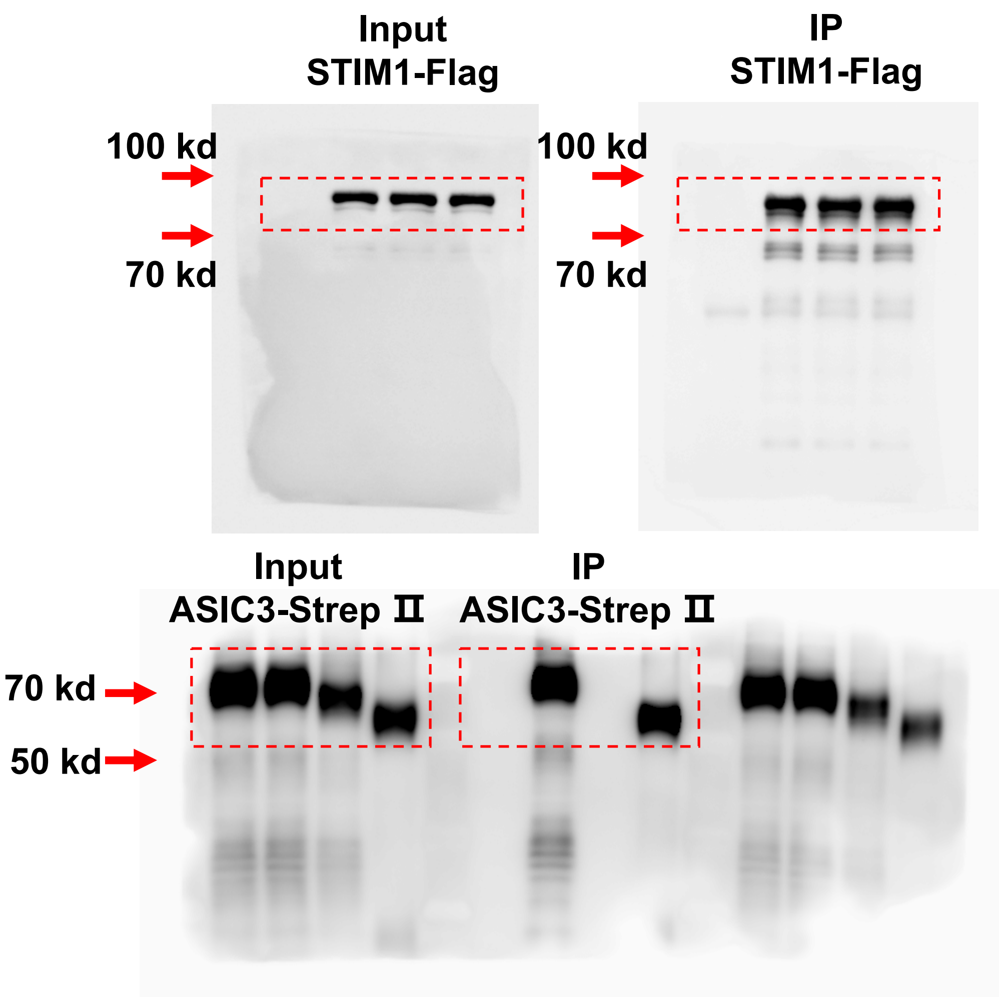

IP: anti-Strep II

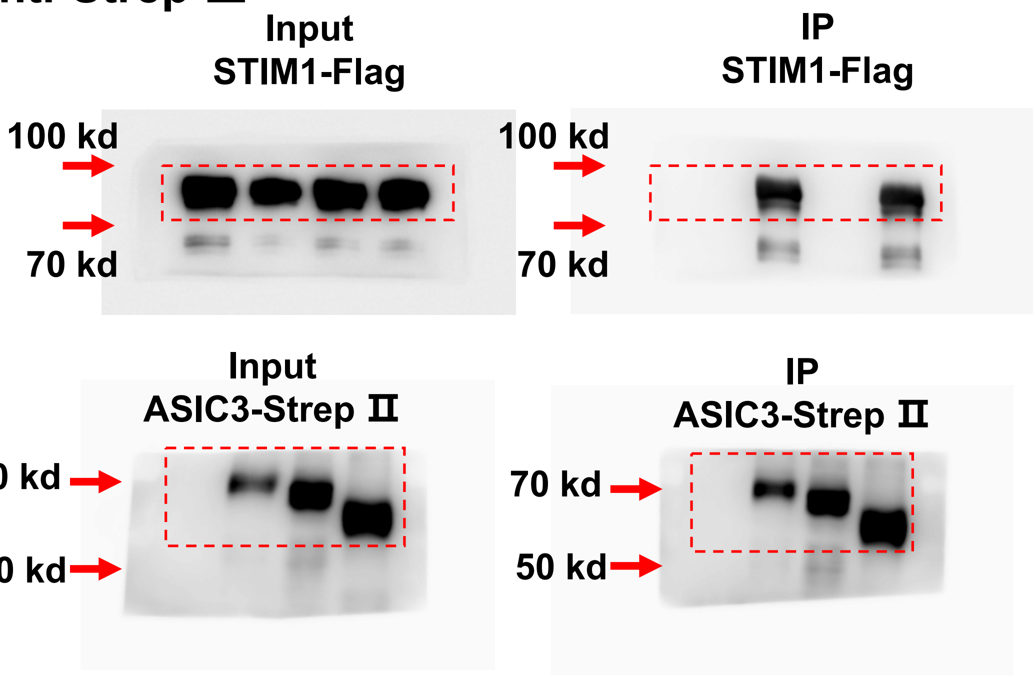

Full unedited gel and blot images for Supplemental Figure 8

used in the figures

Full unedited blot images for Supplemental Figure 8E

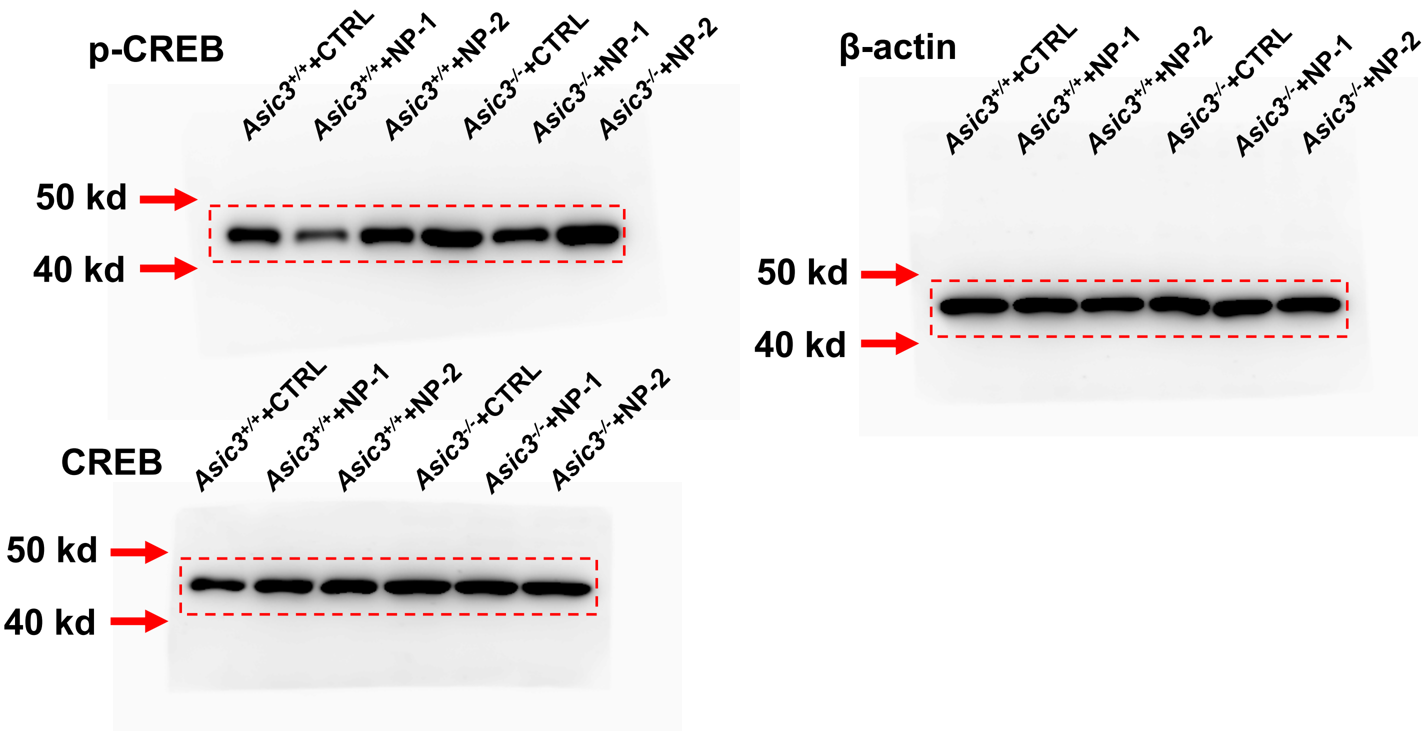

Full unedited gel and blot images for Supplemental Figure 8J

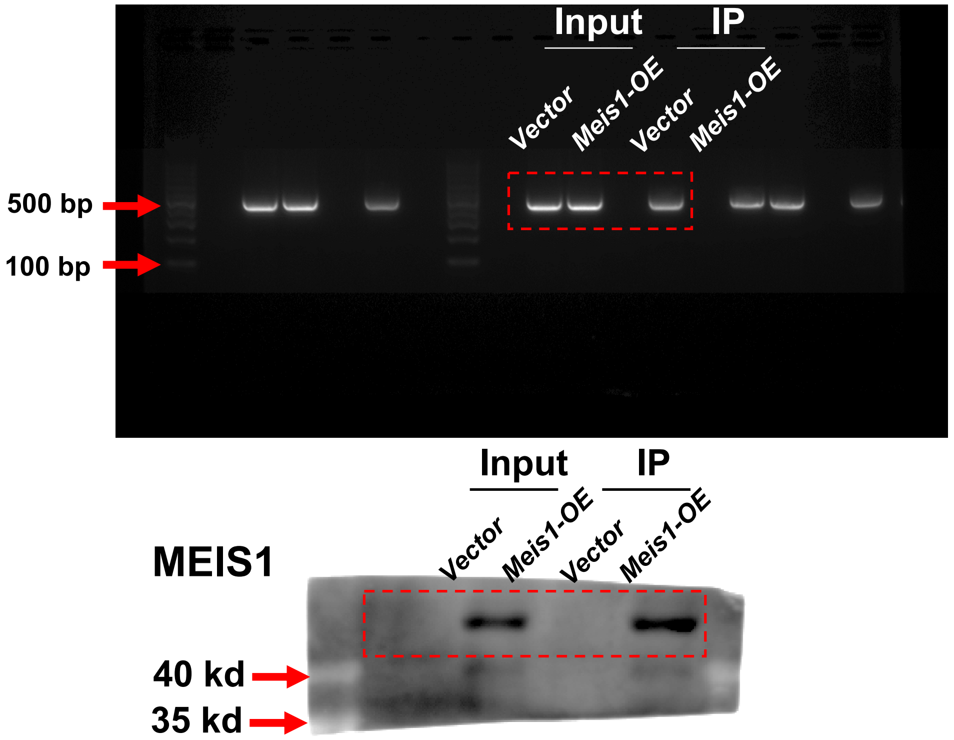

Full unedited blot images for Supplemental Figure 9

used in the figures

Full unedited blot images for Supplemental Figure 9K

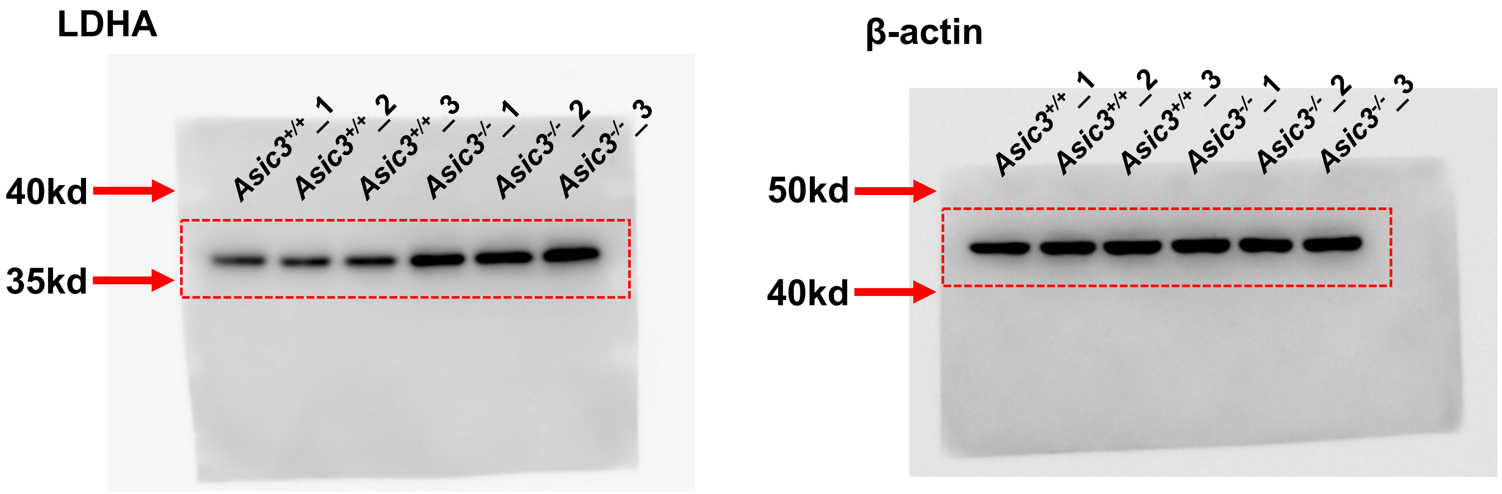

Full unedited blot images for Supplemental Figure 9L

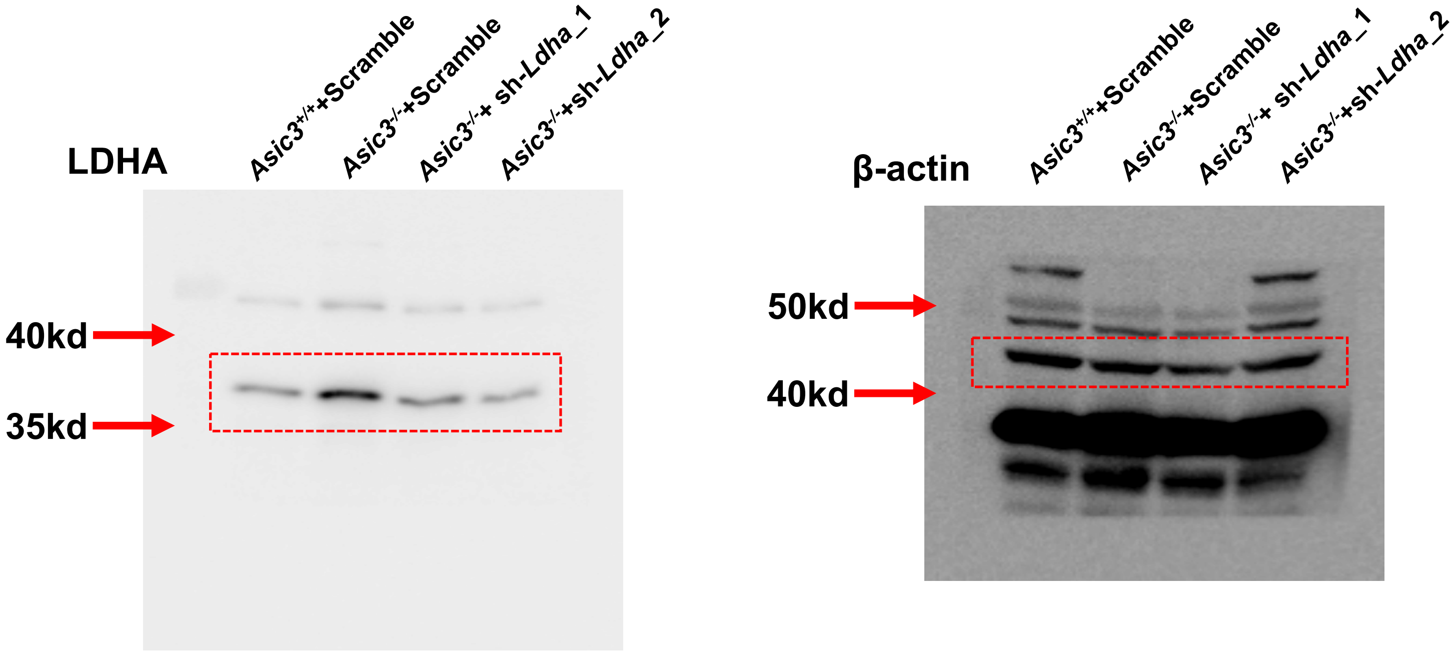

Full unedited blot images for Supplemental Figure 10

used in the figures

Full unedited blot images for Supplemental Figure 10P

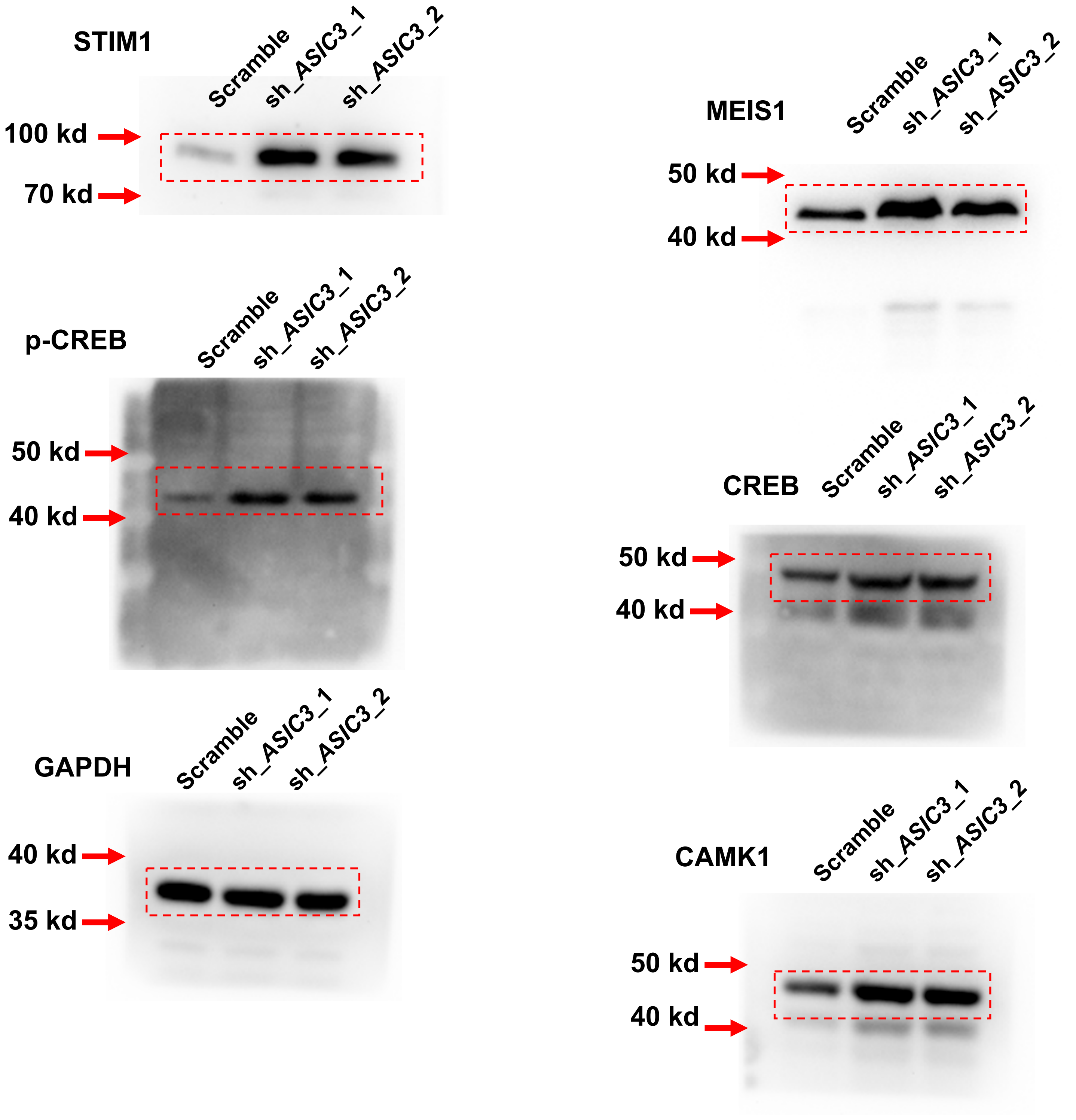

Full unedited blot images for Supplemental Figure 10

used in the figures

Full unedited blot images for Supplemental Figure 10Q

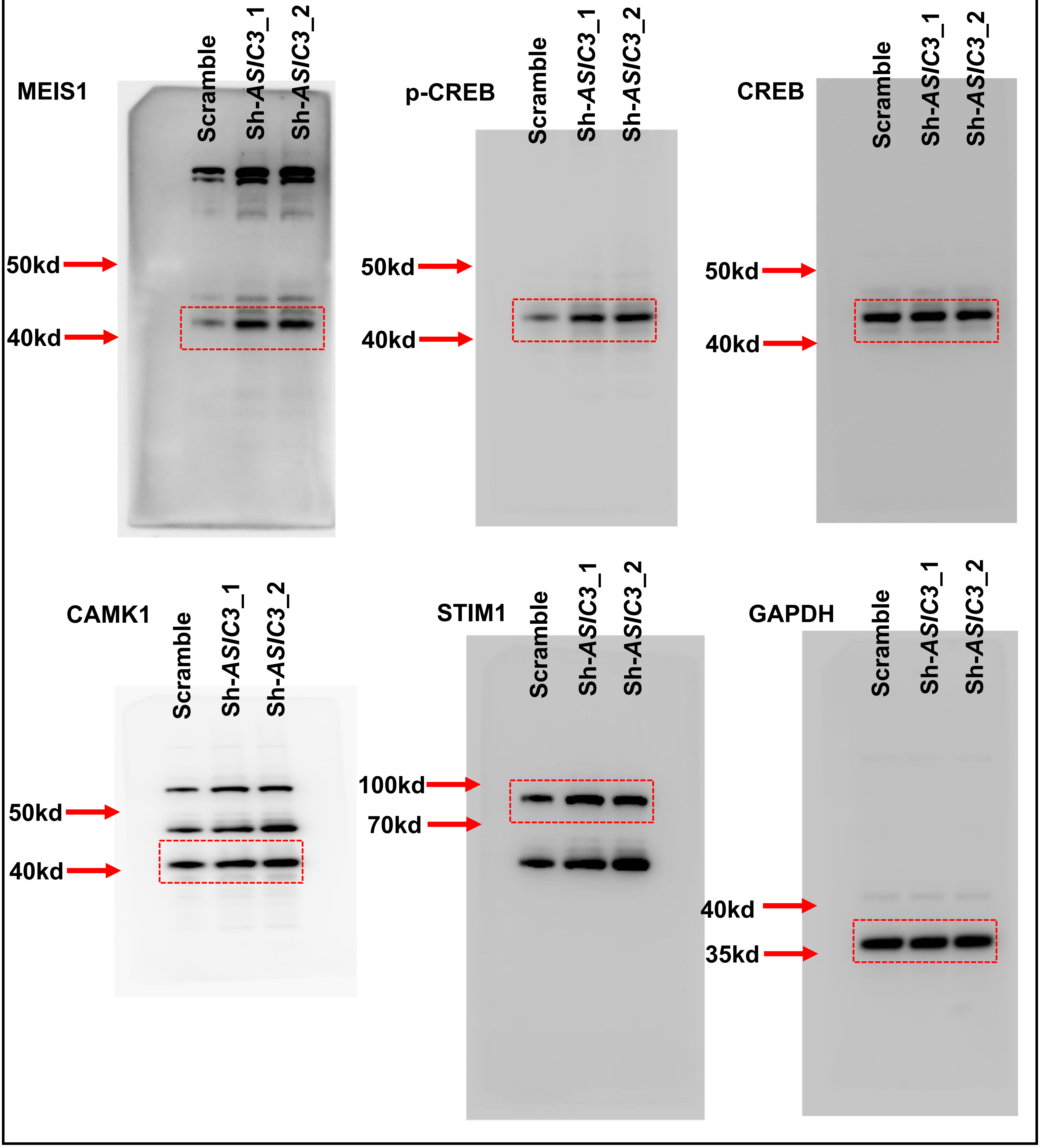

Full unedited blot images for Supplemental Figure 10

used in the figures

Full unedited blot images for Supplemental Figure 10R

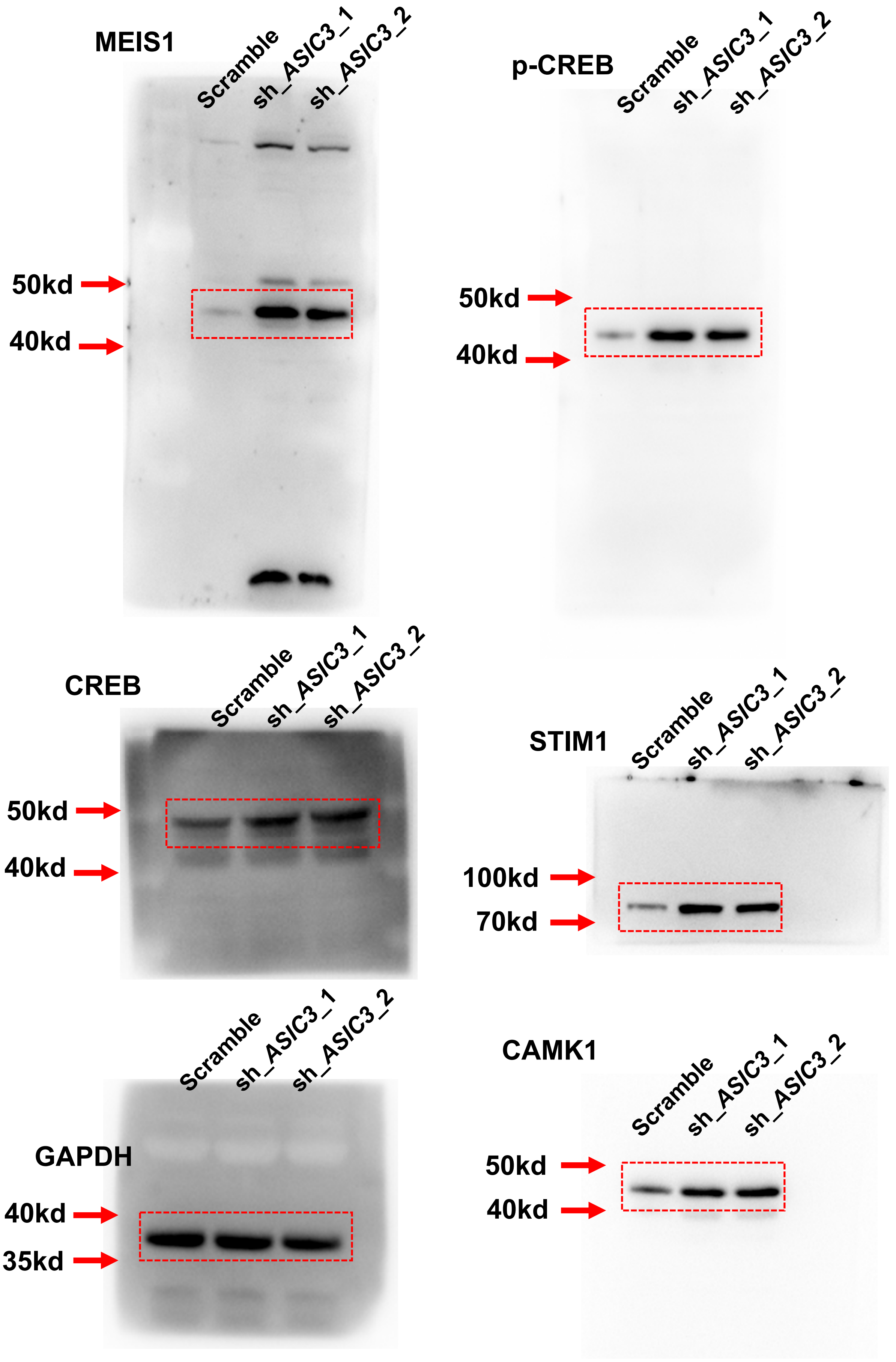

Full unedited blot images for Supplemental Figure 11

used in the figures

Full unedited blot images for Supplemental Figure 11A

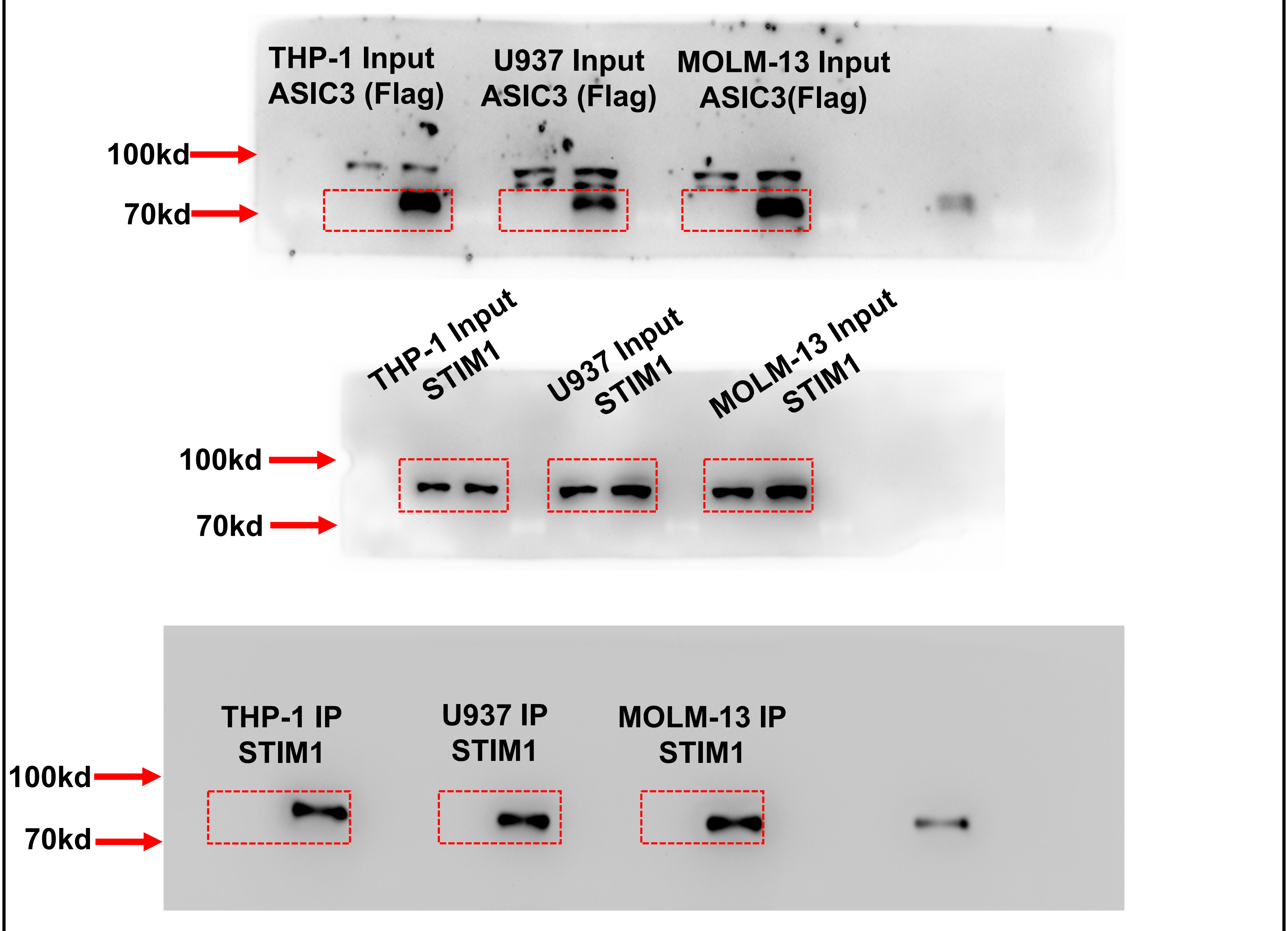

Full unedited blot images for Supplemental Figure 11

used in the figures

Full unedited blot images for Supplemental Figure 11A

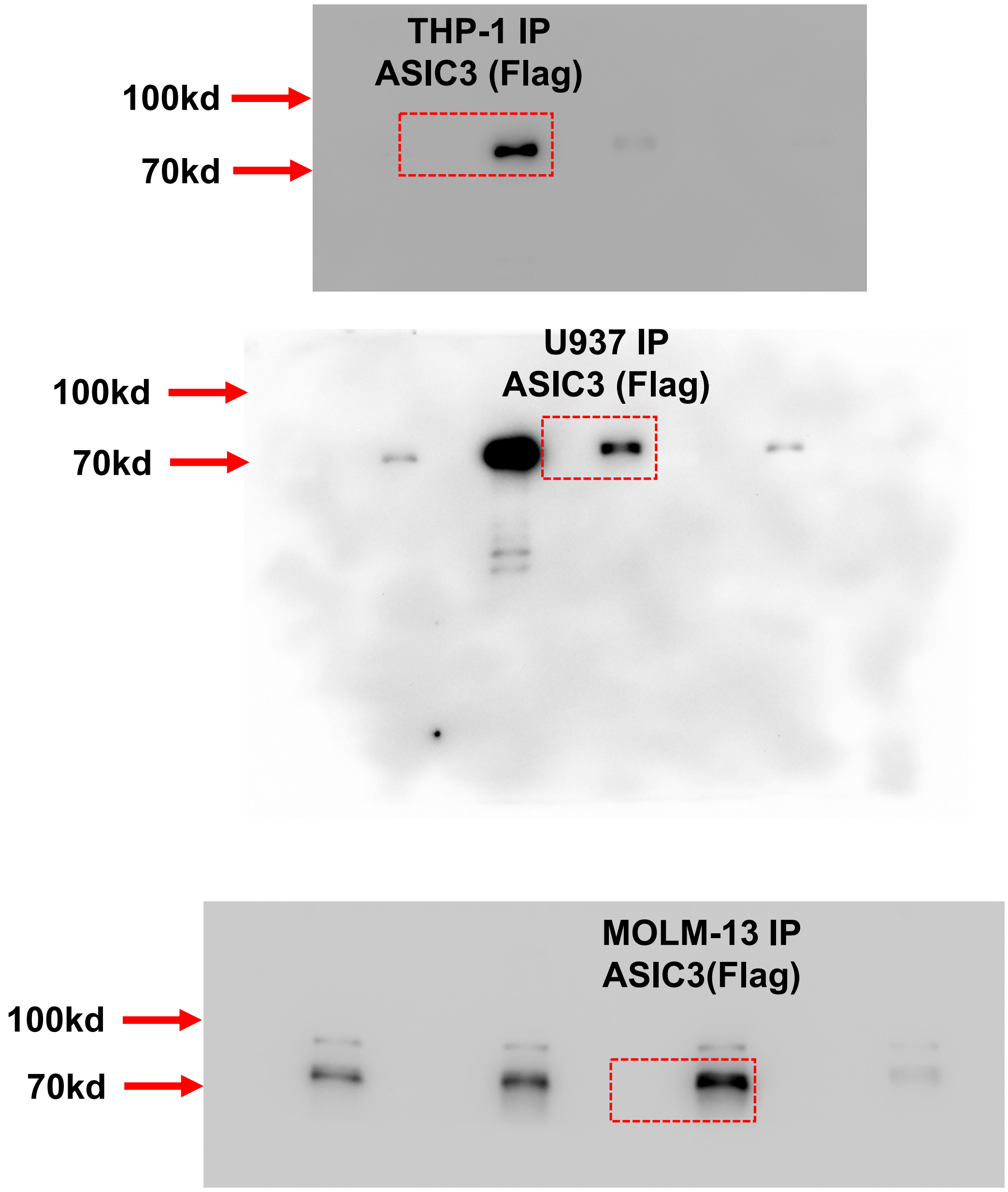

Supplement: Unedited blot and gel images [file jci-135-189051-s073.pdf]
